# Synthesis of 4-functionalized pyrazoles via oxidative thio- or selenocyanation mediated by PhICl_2_ and NH_4_SCN/KSeCN

**DOI:** 10.3762/bjoc.20.128

**Published:** 2024-06-28

**Authors:** Jialiang Wu, Haofeng Shi, Xuemin Li, Jiaxin He, Chen Zhang, Fengxia Sun, Yunfei Du

**Affiliations:** 1 Tianjin Key Laboratory for Modern Drug Delivery & High-Efficiency, School of Pharmaceutical Science and Technology, Tianjin University, Tianjin 300072, China; 2 Hebei Research Center of Pharmaceutical and Chemical Engineering, Hebei University of Science and Technology, Shijiazhuang 050018, China

**Keywords:** PhICl_2_, pyrazoles, selenocyanation, thiocyanation, thiocyanogen chloride

## Abstract

A series of 4-thio/seleno-cyanated pyrazoles was conveniently synthesized from 4-unsubstituted pyrazoles using NH_4_SCN/KSeCN as thio/selenocyanogen sources and PhICl_2_ as the hypervalent iodine oxidant. This metal-free approach was postulated to involve the in situ generation of reactive thio/selenocyanogen chloride (Cl–SCN/SeCN) from the reaction of PhICl_2_ and NH_4_SCN/KSeCN, followed by an electrophilic thio/selenocyanation of the pyrazole skeleton.

## Introduction

**Figure 1:**
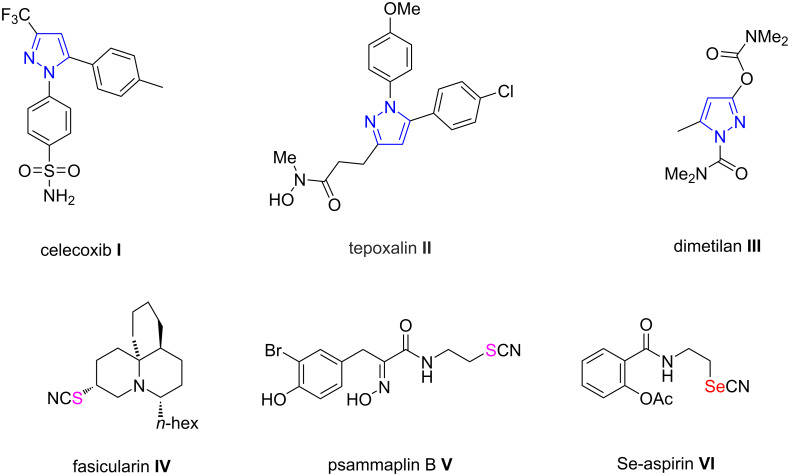
Representative pyrazoles with pharmacological activities and S/Se-containing pharmaceutical molecules.

Pyrazoles and their derivatives are an important class of five-membered heterocyclic compounds [1–5] that have drawn increasing attention from organic chemists, due to their potential biological and pharmaceutical properties including anti-inflammatory [6], antiviral [7], antibacterial [8], antifungal [9], cytotoxic [10], antioxidant [11], and analgesic [12] activities. For instance, celecoxib (**I**, Figure 1) (for treating rheumatoid arthritis and osteoarthritis), tepoxalin (**II**, Figure 1) (a veterinary painkiller used to relieve pain from muscle and bone diseases), dimetilan (**III**, Figure 1) (demonstrating excellent insecticidal effects) [13–15] all possess a pyrazole framework in their respective chemical structure. Considering the pharmaceutical significance of pyrazole compounds, there has been growing interest in the development of efficient strategies for accessing functionalized pyrazole derivatives.

Thio/selenocyano groups are widely existing in the core structural motifs of various natural products and pharmaceutical agents [16–20]. Many S/SeCN-containing bioactive small molecules have been proved to possess wide-ranging biological activities. Specifically, representative examples include fasicularin (**IV**, Figure 1), which possesses cytotoxic properties [21] and psammaplin B (**V**, Figure 1), which shows antimicrobial and mild tyrosine kinase inhibition activities [22]. In addition, Se-aspirin (**VI**, Figure 1) has been used as an effective anti-inflammatory pharmaceutical [23]. On the other hand, organic thiocyanates usually serve as useful synthetic intermediates that can be conveniently converted to sulfur-containing derivatives including sulfides [24], disulfides [25], thiocarbamates [26], and trifluoromethyl thioethers [27]. Likewise, selenocyanates can be used as versatile precursors for the synthesis of a variety of selenium-containing compounds [28–32].

**Scheme 1:**
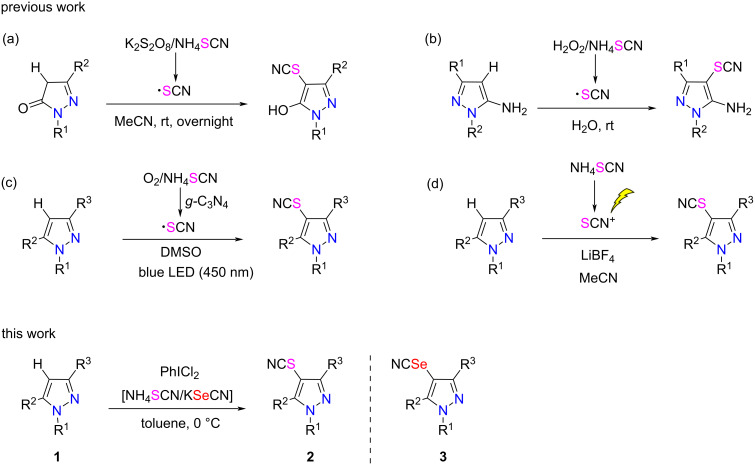
Approaches for thio/selenocyanation of the pyrazole skeleton.

As the S/SeCN-containing organic compounds play an important role in organic and medicinal chemistry, organic chemists have devoted a great deal of efforts to developing efficient thio/selenocyanation approaches [33–41]. Specifically, a plethora of synthetic strategies have been reported for the thiocyanation of heteroaromatic compounds including arenes, indoles, carbazoles, pyrroles, and imidazopyridines [42–45]. However, the electrophilic thiocyanation of biologically important pyrazoles has been less explored [46–48]. Among them, the majority of the reported methods proceed through a radical pathway, with the SCN radical generated by the reaction of the thiocyanate source with a corresponding oxidant (Scheme 1a–c) [49]. For example, Xu reported that a series of 4-thiocyanated 5-hydroxy-1*H*-pyrazoles was synthesized by a K_2_S_2_O_8_-promoted direct thiocyanation of pyrazolin-5-ones at room temperature, using NH_4_SCN as thiocyanogen source (Scheme 1a) [20]. Similarly, utilizing NH_4_SCN and K_2_S_2_O_8_, Yotphan and colleagues realized a direct thiocyanation of *N*-substituted pyrazolones under metal-free conditions [49]. Besides, Choudhury and co-workers developed an additive and metal-free methodology for the C–H thiocyanation of aminopyrazoles, using H_2_O_2_ as a benign oxidizing agent (Scheme 1b) [41]. Pan presented a method for the C–H thiocyanation of pyrazoles by using a sustainable catalyst of graphite-phase carbon nitride (*g-*C_3_N_4_) under visible light irradiation (Scheme 1c) [2]. Furthermore, Yao harnessed an electrochemical approach to form the electrophilic SCN^+^ intermediate, which reacted with pyrazoles to give the corresponding thiocyanated pyrazoles (Scheme 1d) [50]. However, to our knowledge, there are only few reports on the electrophilic selenocyanation of heterocycles [51–53] including the biologically important pyrazoles. In this regard, it should be highly desirable to develop an efficient method for a smooth selenocyanation of pyrazole compounds.

## Results and Discussion

In our previous work we reported that a regioselective C-5 thiocyanation of the 2-pyridone skeleton could be realized via a PhICl_2_-mediated electrophilic thiocyanation approach [54]. Inspired by this previous work, we were interested at investigating whether a direct C-4 selenocyanation as well as a thiocyanation of the pyrazole skeleton could be realized using the same protocol. At the outset of the study, 3,5-dimethyl-1-phenyl-1*H*-pyrazole (**1a**, 1 equiv) was chosen as the model substrate to react with NH_4_SCN (1 equiv) and PhICl_2_ (1 equiv) in THF at 0 °C under N_2_ atmosphere. To our delight, the desired thiocyanated product **2a** was obtained in 68% yield (Table 1, entry 1). Encouraged by this result, we proceeded to investigate the other parameters that would possibly affect the efficiency of the reaction. First, upon a comparison of different reaction temperatures, we found that the reaction operated at 0 °C gave the best result (Table 1, entries 1–3). Then, other SCN-containing inorganic salts including KSCN, AgSCN, and CuSCN were screened, and the results showed that none of them gave better results than NH_4_SCN (Table 1, entries 4–6). Next, other oxidants including phenyliodine(III) diacetate (PIDA), phenyliodine(III) bis(trifluoroacetate) (PIFA), iodosobenzene (PhIO), and NCS were applied, and the results indicated that PhICl_2_ was the most effective oxidant (Table 1, entries 7–10). Later on, when the dosage of PhICl_2_ and NH_4_SCN was increased to 2.0 equivalents, the yield of product **2a** significantly increased to 82% (Table 1, entry 11). However, when the loading of PhICl_2_ and NH_4_SCN were further increased to 3.0 equivalents, the reaction did not afford a better outcome (Table 1, entry 12). Furthermore, solvent screening showed that toluene was the most appropriate solvent, while the reaction led to a much lower yield when DMF, MeOH, MeCN, or DCM were used as solvents (Table 1, entries 13–17). On the basis of the above experimental results, the optimized conditions for the thiocyanation of the model substrate were concluded to be: 2.0 equivalents of PhICl_2_ and NH_4_SCN in toluene at 0 °C, under N_2_ atmosphere (Table 1, entry 17).

**Table 1:** Optimization of oxidative thiocyanation of pyrazole.^a^

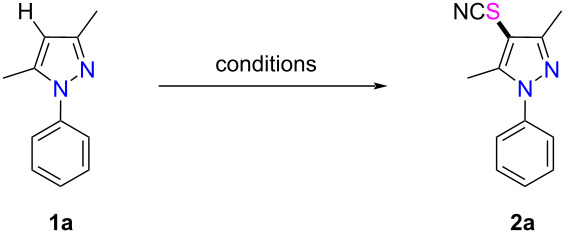					

Entry	Oxidant (equiv)	[SCN] (equiv)	Solvent	*T* (°C)	Yield (%)^b^

1	PhICl_2_ (1.0)	NH_4_SCN (1.0)	THF	0	68
2	PhICl_2_ (1.0)	NH_4_SCN (1.0)	THF	25	43
3	PhICl_2_ (1.0)	NH_4_SCN (1.0)	THF	40	40
4	PhICl_2_ (1.0)	KSCN (1.0)	THF	0	10
5	PhICl_2_ (1.0)	AgSCN (1.0)	THF	0	15
6	PhICl_2_ (1.0)	CuSCN (1.0)	THF	0	12
7	PIDA (1.0)	NH_4_SCN (1.0)	THF	0	NR^c^
8	PIFA (1.0)	NH_4_SCN (1.0)	THF	0	NR
9	PhIO (1.0)	NH_4_SCN (1.0)	THF	0	NR
10	NCS (1.0)	NH_4_SCN (1.0)	THF	0	ND^d^
11	PhICl_2_ (2.0)	NH_4_SCN (2.0)	THF	0	82
12	PhICl_2_ (3.0)	NH_4_SCN (3.0)	THF	0	80
13	PhICl_2_ (2.0)	NH_4_SCN (2.0)	DMF	0	NR
14	PhICl_2_ (2.0)	NH_4_SCN (2.0)	MeOH	0	10
15	PhICl_2_ (2.0)	NH_4_SCN (2.0)	MeCN	0	58
16	PhICl_2_ (2.0)	NH_4_SCN (2.0)	DCM	0	55
**17**	**PhICl** **_2 _** **(2.0)**	**NH** **_4_** **SCN (2.0)**	**toluene**	**0**	**91**

^a^Reaction conditions: under N_2_ atmosphere, a mixture of oxidant and [SCN] in solvent (2 mL) was stirred at 0 °C for 0.5 h, then **1a** (0.20 mmol) was added, and stirring continued at 0 °C for 8 h. ^b^Yield of the isolated product. ^c^NR = no reaction. ^d^ND = no desired product.

**Scheme 2:**
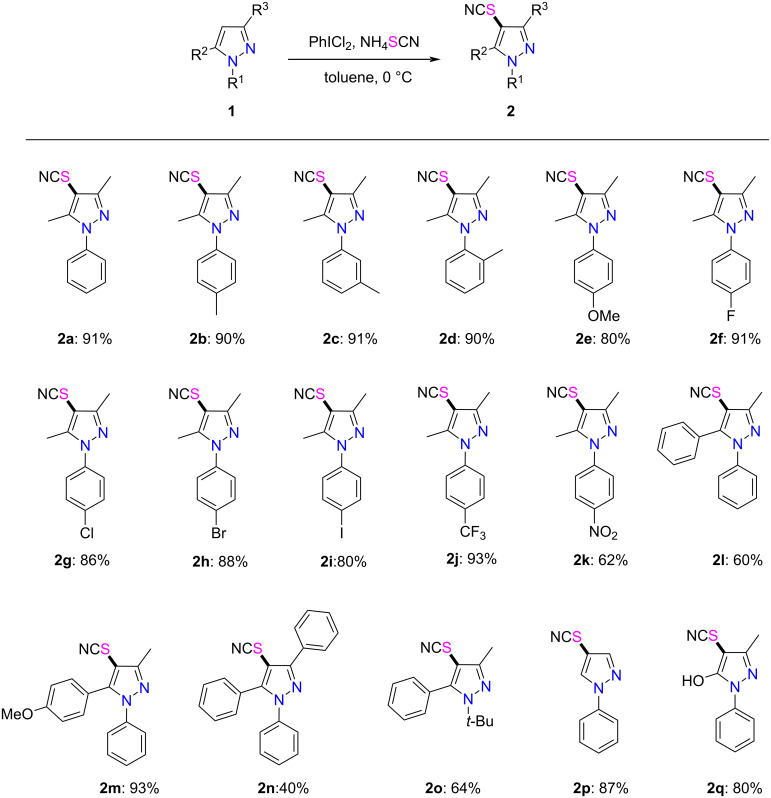
PhICl_2_/NH_4_SCN-mediated thiocyanation of pyrazoles. Reaction conditions: under N_2_ atmosphere, a mixture of PhICl_2_ (2.00 mmol) and NH_4_SCN (2.00 mmol) in toluene (5 mL) was stirred at 0 °C for 0.5 h, then **1a** (1.00 mmol) was added and stirring continued at 0 °C for 8 h. Isolated yields are given.

With the optimized reaction conditions in hand, the substrate scope of this thiocyanation approach was next investigated (Scheme 2). The results showed that the newly established PhICl_2_/NH_4_SCN protocol was suitable for a wide range of substrates. Specifically, when *N*-aryl substrates containing electron-donating groups (-Me, -OMe) were subjected to the standard reaction conditions, the corresponding products **2b**–**e** were obtained in good yields (80–91%). It was found that there was no significant influence on the outcome of the reactions of various *N*-aryl-substituted pyrazoles with a methyl group at the *ortho*-, *meta*- or *para*- positions of the phenyl group. Next, *N*-arylated substrates bearing electron-withdrawing groups (-F, -Cl, -Br, -I, -CF_3_, -NO_2_) were tested, and the desired products **2f**–**k** were conveniently obtained in moderate to good yields. Notably, the reaction of the substrate bearing a -CF_3_ group afforded the corresponding product **2j** in 93% yield. However, the substrate possessing a -NO_2_ substituent gave an inferior yield of the product **2k**. Then, we proceeded to investigate the effects of different substituents R^2^ and R^3^. When the methyl substituent (R^2^) was replaced with an aryl group, the corresponding thiocyanated products **2l**–**o** could be obtained in acceptable to moderate yields. On the other hand, the method was equally applicable to the substrate bearing two aryl substituents (R^2^ and R^3^), albeit the reaction afforded product **2n** in a much lower yield, possibly caused by steric congestion. In addition, when the aryl substituent of R^1^ was replaced with a *tert*-butyl group, this method also worked well to give product **2o** in moderate yield. Notably, when the C3 and C5-unsubstituted substrate **1p** was subjected to the standard conditions, the 4-thiocyanated product **2p** was obtained regioselectively in 87% yield. Strikingly, the thiocyanation of the pharmaceutically active compound edaravone could also be realized under the optimized conditions, affording the corresponding product **2q** in good yield.

**Scheme 3:**
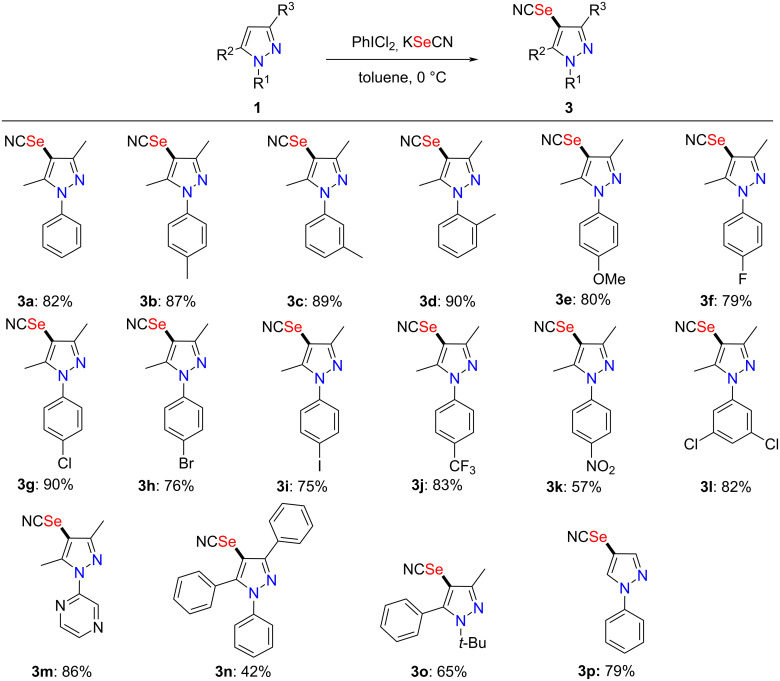
PhICl_2_/KSeCN-mediated selenocyanation of pyrazoles. Reaction conditions: under N_2_ atmosphere, a mixture of PhICl_2_ (2.00 mmol) and KSeCN (2.00 mmol) in toluene (5 mL) was stirred at 0 °C for 0.5 h, then **1a** (1.00 mmol) was added and stirring continued at 0 °C for 8 h. Isolated yields are given.

Furthermore, we turned our attention to the applicability of this protocol for the selenocyanation of the pyrazole skeleton (Scheme 3). Gratifyingly, the method was equally applicable to selenocyanation of pyrazoles bearing various substituents, with the corresponding selenocyanated products **3a**–**o** achieved in acceptable to good yields. Similarly, the selenocyanation of C3- and C5-unsubstituted substrate **1p** regioselectively furnished the 4-selenocyanated pyrazole **3p** in good yield.

**Scheme 4:**
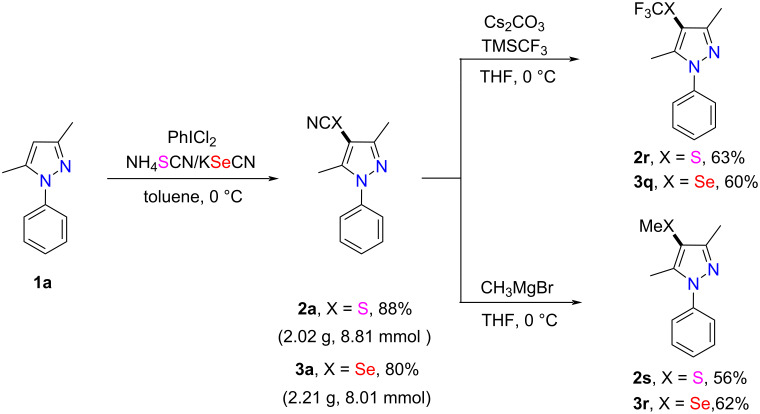
Gram-scale synthesis of compounds **2a** and **3a** and their derivatization.

The utility of this approach was further demonstrated by a scale-up experiment. When 10.0 mmol of compound **1a** were treated with 20.0 mmol of NH_4_SCN/KSeCN and PhICl_2_ under the standard reaction conditions, the desired products **2a** and **3a** were obtained in 88% and 80% yield, respectively (Scheme 4).

The obtained 4-thio/selenocyanated pyrazoles could be further derivatized by known approaches. Specifically, products **2a** and **3a** could react with TMSCF_3_ in the presence of Cs_2_CO_3_ [55] to give the corresponding SCF_3_- and SeCF_3_-containing compounds **2r** and **3q** in moderate yields. Moreover, products **2a** and **3a** could be conveniently transformed into thiomethyl and selenomethyl-substituted pyrazole derivatives **2s** and **3r** by treatment with CH_3_MgBr in THF [56] (Scheme 4).

**Scheme 5:**
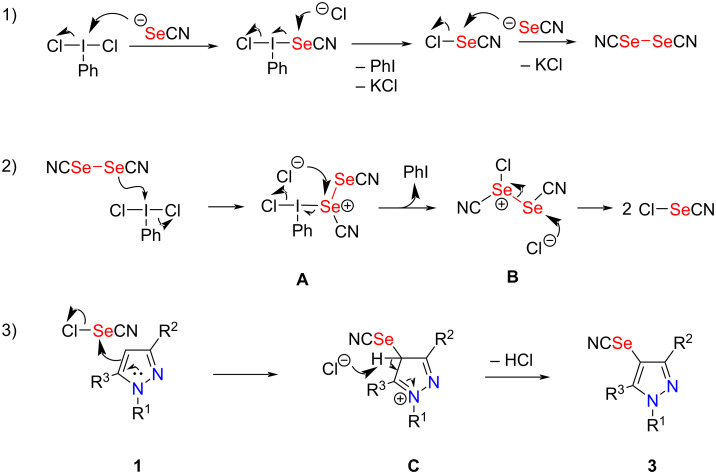
Plausible reaction mechanism.

Based on the previous reports [54,57–59], a possible mechanism of this selenocyanation reaction was proposed (Scheme 5). First, the reaction of PhICl_2_ with KSeCN produces selenocyanogen chloride (Cl–SeCN), which further reacts with selenocyanate to give (SeCN)_2_ [60]. Then, one selenium atom of (SeCN)_2_ nucleophilically attacks the iodine center in PhICl_2_ to generate intermediate **A**, which was further transformed into intermediate **B** by release of one molecule of iodobenzene. Next, the nucleophilic attack of chloride anion to the bivalent selenium center of intermediate **B** resulted in the formation of two molecules of Cl–SeCN. Subsequently, Cl–SeCN undergoes an electrophilic addition reaction with pyrazole **1** to give intermediate **C**, which, after deprotonative rearomatization affords the 4-selenocyanated pyrazole **3**.

## Conclusion

In conclusion, we have accomplished the synthesis of a series of C-4 thio/selenocyanated pyrazoles via a hypervalent iodine-mediated electrophilic thio/selenocyanation approach under mild reaction conditions. Furthermore, the obtained S/SeCN-containing pyrazoles can be converted to S/SeCF_3_- and S/SeMe-containing pyrazole derivatives. Further investigations of the synthetic utility of this approach are currently ongoing in our lab.

## Supporting Information

File 1 Synthetic details and compound characterization data.

## Data Availability

All data that supports the findings of this study is available in the published article and/or the supporting information to this article.
