# Supplementary material for: Synthesis of 4-functionalized pyrazoles via oxidative thio- or selenocyanation mediated by PhICl2 and NH4SCN/KSeCN
Source: Beilstein J Org Chem. 2024 Jun 28;20:1453–61. doi: 10.3762/bjoc.20.128 (PMC11216092; doi:10.3762/bjoc.20.128)
Supplement: File 1 — Synthetic details and compound characterization data. [file Beilstein_J_Org_Chem-20-1453-s001.pdf]

BEILSTEIN JOURNAL OF ORGANIC CHEMISTRY

Supporting Information

for

Synthesis of 4-functionalized pyrazoles via oxidative thio- or selenocyanation mediated by $PhICl_2$ and NH_4SCN/KSeCN

Jialiang Wu, Haofeng Shi, Xuemin Li, Jiaxin He, Chen Zhang, Fengxia Sun and Yunfei Du

Beilstein J. Org. Chem. **2024,** *20,* 1453–1461. doi:10.3762/bjoc.20.128

Synthetic details and compound characterization data

License and Terms: This is a supporting information file under the terms of the Creative Commons Attribution License (https://creativecommons.org/licenses/by/4.0). Please note that the reuse, redistribution and reproduction in particular requires that the author(s) and source are credited and that individual graphics may be subject to special legal provisions.
The license is subject to the *Beilstein Journal of Organic Chemistry* terms and conditions: (https://www.beilstein-journals.org/bjoc/terms)

Table of contents

**I**	General information	S2
**II**	Experimental procedures	S3–S4
**III**	Spectroscopic data	S5–S21
**IV**	References	S21
**V**	NMR spectra of products	S22–S58

I. General information

^{1}H and ^{13}C NMR spectra were recorded on a 400 MHz or 600 MHz spectrometer at 25 °C. Chemical shift values are given in ppm and referred to TMS (0.00 ppm) as the internal standard. Chemical shifts were expressed in parts per million (δ) downfield from the internal standard tetramethylsilane, and were reported as s (singlet), d (doublet), t (triplet), q (quadruple), dd (doublet of doublet), m (multiplet), etc. The coupling constants *J*, are reported in hertz (Hz). High resolution mass spectrometry (HRMS) was obtained on a Q-TOF micro spectrometer. Melting points were determined with a Micromelting point apparatus. TLC plates were visualized by exposure to ultraviolet light.

Reagents and solvents were purchased as reagent grade and were used without further purification. All reactions were performed in standard glassware, dried by heating at 70 °C for 3 h before use. The starting materials **1** [1] were prepared according to literature methods. Flash column chromatography was performed over silica gel (200–300 mesh) using a mixture of ethyl acetate (EtOAc) and petroleum ether (PE) as eluent.

II. Experimental procedures

1. General procedure for the preparation of 4-thio/selenocyanated pyrazoles

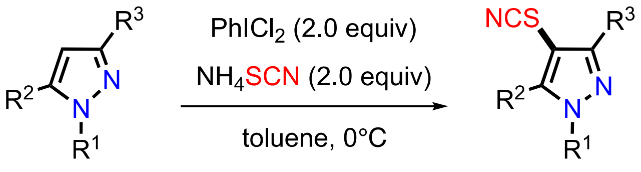

1 **2**

General procedure A: Under N_2 atmosphere, to a solution of NH_4SCN (2.0 mmol) in toluene (5 mL) was added $PhICl_2$ (2.0 mmol) at 0 °C. The mixture was stirred for 30 min. Then, substrate **1** (1.0 mmol) was added to this solution in one portion and the reaction mixture was stirred for about 8 h until completion consumption of the starting material (monitored by TLC). Then, the reaction mixture was washed with water (10 mL) and extracted with DCM (3 × 10 mL). The combined organic layers were dried with $MgSO_4$ and concentrated in vacuo and the residue was purified by flash column chromatography to afford the corresponding 4-thiocyanated pyrazoles **2**.

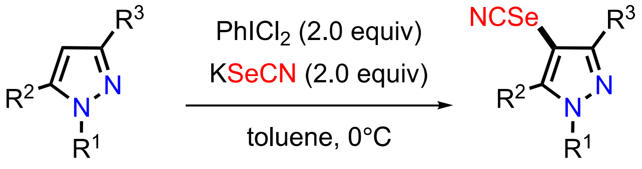

1 **3**

General procedure B: Under N_2 atmosphere, to a solution of KSeCN (2.0 mmol) in toluene (5 mL) was added $PhICl_2$ (2.0 mmol) at 0 °C. The mixture was stirred for 30 min. Then, substrate **1** (1.0 mmol) was added to this solution in one portion and the reaction mixture was stirred for about 8 h until complete consumption of the starting material (monitored by TLC). Then the reaction mixture was washed with water (10 mL) and extracted with DCM (3 × 10 mL). The combined organic layers were dried with $MgSO_4$ and concentrated in vacuo and the residue was purified by flash column chromatography to afford the corresponding 4-selenocyanated pyrazoles **3**.

2. General procedure for the gram-scale synthesis of compounds 2a and 3a.

General procedure for the gram-scale synthesis of **2a**: Under N_2 atmosphere, to a solution of NH_4SCN (20.0 mmol) in toluene (20 mL) was added $PhICl_2$ (20.0 mmol) at 0 °C. The mixture was stirred for 1 h, and then substrate **1a** (10.0 mmol) was added to this solution in one portion and the reaction mixture was stirred for about 10 h until complete consumption of the starting material (monitored by TLC). Then the reaction mixture was washed with water (30 mL) and extracted with DCM (5 × 15 mL). The combined organic layers were dried with $MgSO_4$ and concentrated in vacuo. The residue was purified by flash column chromatography to afford the corresponding desired product **2a** (2.02 g, 88%).

General procedure for the gram-scale synthesis of **3a**: Under N_2 atmosphere, to a solution of KSeCN (20.0 mmol) in toluene (20 mL) was added $PhICl_2$ (20.0 mmol) at 0 °C. The mixture was stirred for 1 h. Then substrate **1a** (10.0 mmol) was added to this solution in one portion and the reaction mixture was stirred for about 10 h until complete consumption of the starting material (monitored by TLC). Then the reaction mixture was washed with water (30 mL) and extracted with DCM (5 × 15 mL). The combined organic layers were dried with $MgSO_4$ and concentrated in vacuo. The residue was purified by flash column chromatography to afford the corresponding desired product **3a** (2.21 g, 80%).

III. Spectroscopic data

3,5-Dimethyl-1-phenyl-4-thiocyanato-1*H*-pyrazole (2a)

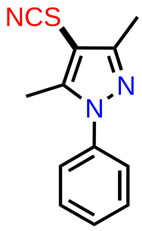

According to the procedure A, **2a** was purified by silica gel chromatography (10% EtOAc/PE). A white solid (208.6 mg, yield: 91%). mp: 99-100 °C. ^{1}H NMR (400 MHz, $CDCl_3$) δ 7.54 – 7.45 (m, 2H), 7.43 (d, *J* = 7.3 Hz, 1H), 7.41 – 7.37 (m, 2H), 2.43 (s, 3H), 2.42 (s, 3H). ^{13}C NMR (101 MHz, $CDCl_3$) δ 152.0, 144.3, 139.1, 129.4, 128.6, 125.0, 110.9, 96.6, 12.0, 11.5. HRMS (ESI) calcd for $C_{12}H_{11}N_3NaS^+$ [M + Na^+] 252.0566, found 252.0570.

The characterization data of **2a** agree with the literature values. [2]

3,5-Dimethyl-4-thiocyanato-1-(*p*-tolyl)-1*H*-pyrazole (2b)

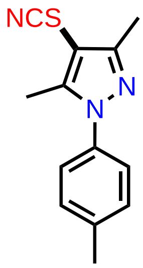

According to the procedure A, **2b** was purified by silica gel chromatography (10% EtOAc/petroleum ether). A white solid (218.9 mg, yield: 90%). mp: 87-88 °C. ^{1}H NMR (400 MHz, $CDCl_3$) δ 7.28 – 7.26 (m, 4H), 2.42 (s, 3H), 2.41 (s, 3H), 2.40 (s, 3H). ^{13}C NMR (101 MHz, $CDCl_3$) δ 151.8, 144.3, 138.7, 136.6, 129.9, 124.8, 111.0, 96.2, 21.2, 12.0, 11.5. HRMS (ESI) calcd for $C_{13}H_{13}N_3NaS^+$ [M + Na^+] 266.0722, found 266.0727.

The characterization data of **2b** agree with the literature values. [2]

3,5-Dimethyl-4-thiocyanato-1-(*m*-tolyl)-1*H*-pyrazole (2c)

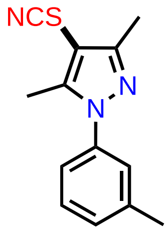

According to the procedure A, **2c** was purified by silica gel chromatography (10% EtOAc/PE). Yellow oil (221.4 mg, yield: 91%). ^{1}H NMR (400 MHz, $CDCl_3$) δ 7.30 (t, *J* = 7.8 Hz, 1H), 7.18 (d, *J* = 9.5 Hz, 2H), 7.10 (d, *J* = 8.1 Hz, 1H), 2.36 (d, *J* = 3.7 Hz, 9H). ^{13}C NMR (101 MHz, $CDCl_3$) δ 151.9, 144.3, 139.7, 139.0, 129.4, 129.1, 125.7, 121.9, 110.9, 96.4, 21.3, 12.0, 11.5. HRMS (ESI) calcd for $C_{13}H_{13}N_3NaS^+$ [M + Na^+] 266.0722, found 266.0727.

The characterization data of **2c** agree with the literature values. [2]

3,5-Dimethyl-4-thiocyanato-1-(*o*-tolyl)-1*H*-pyrazole (2d)

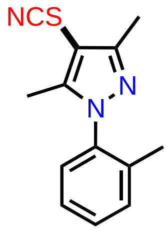

According to the procedure A, **2d** was purified by silica gel chromatography (10% EtOAc/PE). Yellow oil (218.9 mg, yield: 90%). ^{1}H NMR (400 MHz, $CDCl_3$) δ 7.43 – 7.28 (m, 3H), 7.19 (dd, *J* = 7.7, 1.1 Hz, 1H), 2.43 (s, 3H), 2.22 (s, 3H), 2.05 (s, 3H). ^{13}C NMR (101 MHz, $CDCl_3$) δ 151.7, 145.2, 137.8, 135.8, 131.2, 129.9, 127.5, 126.9, 111.0, 95.2, 17.2, 12.1, 10.6. HRMS (ESI) calcd for $C_{13}H_{13}N_3NaS^+$ [M + Na^+] 266.0722, found 266.0725.

The characterization data of **2d** agree with the literature values. [2]

1-(4-Methoxyphenyl)-3,5-dimethyl-4-thiocyanato-1*H*-pyrazole (2e)

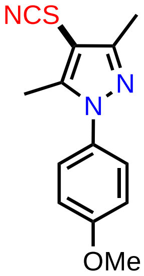

According to the procedure A, **2e** was purified by silica gel chromatography (10% EtOAc/PE). A yellow solid (207.4 mg, yield: 80%). mp: 104-105 °C. ^{1}H NMR (400 MHz, $CDCl_3$) δ 7.33 – 7.27 (m, 2H), 7.01 – 6.96 (m, 2H), 3.86 (s, 3H), 2.42 (s, 3H), 2.38 (s, 3H). ^{13}C NMR (101 MHz, $CDCl_3$) δ 159.7, 151.7, 144.5, 132.0, 126.5, 114.5, 111.0, 95.9, 55.6, 12.0, 11.4. HRMS (ESI) calcd for $C_{13}H_{13}N_3NaOS^+$ [M + Na^+] 282.0672, found 282.0676.

The characterization data of **2e** agree with the literature values. [2]

1-(4-Fluorophenyl)-3,5-dimethyl-4-thiocyanato-1*H*-pyrazole (2f)

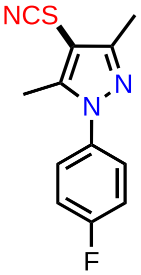

According to the procedure A, **2f** was purified by silica gel chromatography (10% EtOAc/PE). A white solid (225 .0 mg, yield: 91%). mp: 81-82 °C. ^{1}H NMR (400 MHz, $CDCl_3$) δ 7.41 – 7.35 (m, 2H), 7.24 – 7.15 (m, 2H), 2.42 (s, 3H), 2.41 (s, 3H). ^{13}C NMR (101 MHz, $CDCl_3$) δ 162.4 (d, J_{C-F} = 250.0 Hz), 152.1, 144.5, 135.2 (d, J_{C-F} = 3.0 Hz), 127.0 (d, J_{C-F} = 8.1 Hz), 116.4 (d, J_{C-F} = 23.2 Hz), 110.7, 96.8, 12.0, 11.4. HRMS (ESI) calcd for $C_{12}H_{10}FN_3NaS^+$ [M + Na^+] 270.0472, found 270.0476.

The characterization data of **2f** agree with the literature values. [2]

1-(4-Chlorophenyl)-3,5-dimethyl-4-thiocyanato-1*H*-pyrazol (2g)

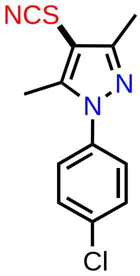

According to the procedure A, **2g** was purified by silica gel chromatography (10% EtOAc/PE). A white solid (226.8 mg, yield: 86%). mp: 95-98 °C. ^{1}H NMR (400 MHz, $CDCl_3$) δ 7.48 (d, *J* = 8.6 Hz, 2H), 7.36 (d, *J* = 8.7 Hz, 2H), 2.44 (s, 3H), 2.42 (s, 3H). ^{13}C NMR (101 MHz, $CDCl_3$) δ 152.3, 144.4, 137.5, 134.5, 129.6, 126.1, 110.7, 97.2, 12.0, 11.6. HRMS (ESI) calcd for $C_{12}H_{10}ClN_3NaS^+$ [M + Na^+] 252.0566, found 252.0570.

The characterization data of **2g** agree with the literature values. [2]

1-(4-Bromophenyl)-3,5-dimethyl-4-thiocyanato-1*H*-pyrazole (2h)

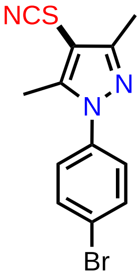

According to the procedure A, **2h** was purified by silica gel chromatography (10% EtOAc/PE). A white solid (271.2 mg, yield: 88%). mp: 111-113 °C. ^{1}H NMR (400 MHz, $CDCl_3$) δ 7.66 – 7.61 (m, 2H), 7.32 – 7.28 (m, 2H), 2.44 (s, 3H), 2.42 (s, 3H). ^{13}C NMR (101 MHz, $CDCl_3$) δ 152.4, 144.4, 138.0, 132.6, 126.4, 122.4, 110.6, 97.3, 12.0, 11.6. HRMS (ESI) calcd for $C_{12}H_{10}BrN_3NaS^+$ [M + Na^+] 329.9671, found 329.9676.

The characterization data of **2h** agree with the literature values. [2]

1-(4-Iodophenyl)-3,5-dimethyl-4-thiocyanato-1*H*-pyrazole (2i)

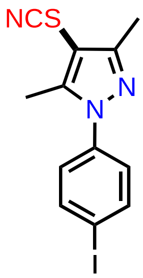

According to the procedure A, **2i** was purified by silica gel chromatography (10% EtOAc/PE). A white solid (284.1 mg, yield: 80%). mp: 103-105 °C. ^{1}H NMR (400 MHz, $CDCl_3$) δ 7.86 – 7.80 (m, 2H), 7.19 – 7.15 (m, 2H), 2.44 (s, 3H), 2.42 (s, 3H). ^{13}C NMR (101 MHz, $CDCl_3$) δ 152.4, 144.3, 138.7, 138.5, 126.5, 110.6, 97.3, 93.8, 12.0, 11.6. HRMS (ESI) calcd for $C_{12}H_{10}IN_3NaS^+$ [M + Na$^+$] 377.9532, found 377.9537.

The characterization data of **2i** agree with the literature values. [2]

3,5-Dimethyl-4-thiocyanato-1-(4-(trifluoromethyl)phenyl)-1*H*-pyrazole (2j)

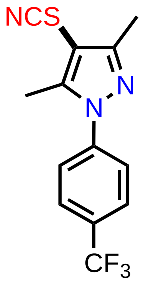

According to the procedure A, **2j** was purified by silica gel chromatography (10% EtOAc/PE). A yellow solid (276.4 mg, yield: 93%). mp: 67-70 °C. ^{1}H NMR (400 MHz, $CDCl_3$) δ 7.77 (d, *J* = 8.4 Hz, 2H), 7.58 (d, *J* = 8.3 Hz, 2H), 2.50 (s, 3H), 2.44 (s, 3H). ^{13}C NMR (101 MHz, $CDCl_3$) δ 152.7, 144.5, 141.8, 130.4 (q, $J_{C\text{-}F}$ = 33.3 Hz), 126.6 (q, $J_{C\text{-}F}$ = 3.7 Hz), 124.5, 123.6 (q, $J_{C\text{-}F}$ = 273.3 Hz), 110.5, 98.1, 12.0, 11.8. HRMS (ESI) calcd for $C_{13}H_{10}F_3N_3NaS^+$ [M + Na$^+$] 320.0440, found 320.0445.

The characterization data of **2j** agree with the literature values. [2]

3,5-Dimethyl-1-(4-nitrophenyl)-4-thiocyanato-1*H*-pyrazole (2k)

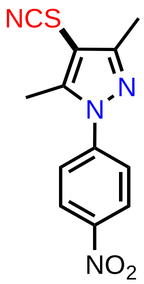

According to the procedure A, **2k** was purified by silica gel chromatography (10% EtOAc/PE). A white solid (170.0 mg, yield: 62%). mp: 80-82 °C. ^{1}H NMR (400 MHz, $CDCl_3$) δ 8.41 – 8.35 (m, 2H), 7.69 – 7.64 (m, 2H), 2.56 (s, 3H), 2.45 (s, 3H). ^{13}C NMR (101 MHz, $CDCl_3$) δ 153.3, 146.8, 144.6, 143.9, 125.0, 124.6, 110.2, 99.3, 12.1, 12.1. HRMS (ESI) calcd for $C_{12}H_{10}N_4NaO_2S^+$ [M + Na^+] 297.0417, found 297.0420.

3-Methyl-1,5-diphenyl-4-thiocyanato-1*H*-pyrazole (2l)

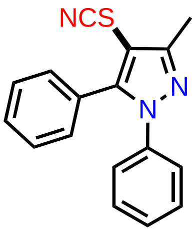

According to the procedure A, **2l** was purified by silica gel chromatography (10% EtOAc/PE). A yellow solid (174.8 mg, yield: 60%). mp: 50-54 °C. ^{1}H NMR (400 MHz, $CDCl_3$) δ 7.46 – 7.38 (m, 3H), 7.34 – 7.25 (m, 5H), 7.24 – 7.18 (m, 2H), 2.54 (s, 3H). ^{13}C NMR (101 MHz, $CDCl_3$) δ 152.8, 147.0, 139.2, 130.0, 129.7, 129.1, 128.8, 128.1, 127.9, 124.9, 111.2, 97.7, 12.2. HRMS (ESI) calcd for $C_{17}H_{13}N_3NaS^+$ [M + Na^+] 314.0722, found 314.0726.

5-(4-Methoxyphenyl)-3-methyl-1-phenyl-4-thiocyanato-1*H*-pyrazol (2m)

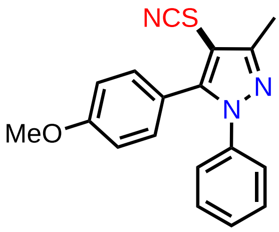

According to the procedure A, **2m** was purified by silica gel chromatography (10%

EtOAc/PE). A white solid (298.9 mg, yield: 93%). mp: 95-98 °C. ^{1}H NMR (400 MHz, $CDCl_3$) δ 7.32 (q, J = 5.5 Hz, 3H), 7.25 – 7.17 (m, 4H), 6.95 – 6.90 (m, 2H), 3.84 (s, 3H), 2.53 (s, 3H). ^{13}C NMR (101 MHz, $CDCl_3$) δ 160.5, 152.7, 147.0, 139.4, 131.3, 129.1, 128.0, 125.0, 119.9, 114.3, 111.4, 97.3, 55.3, 12.2. HRMS (ESI) calcd for $C_{18}H_{15}N_3NaOS^+$ [M + Na$^+$] 344.0828, found 344.0820.

1,3,5-Triphenyl-4-thiocyanato-1*H*-pyrazole (2n)

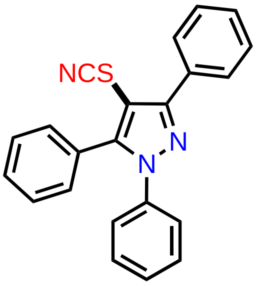

According to the procedure A, **2n** was purified by silica gel chromatography (10% EtOAc/PE). A yellow solid (141.37 mg, yield: 40%). mp: 110-112 °C. ^{1}H NMR (400 MHz, $CDCl_3$) δ 8.04 – 7.96 (m, 2H), 7.58 – 7.45 (m, 6H), 7.40 – 7.31 (m, 7H). ^{13}C NMR (101 MHz, $CDCl_3$) δ 154.3, 148.6, 139.2, 131.0, 130.2, 129.9, 129.2, 129.1, 128.9, 128.7, 128.5, 128.3, 127.8, 125.0, 111.8, 96.7. HRMS (ESI) calcd for $C_{22}H_{15}N_3NaS^+$ [M + Na$^+$] 376.0879, found 376.0875.

The characterization data of **2n** agree with the literature values. [3]

1-(*tert*-Butyl)-3-methyl-5-phenyl-4-thiocyanato-1*H*-pyrazole (2o)

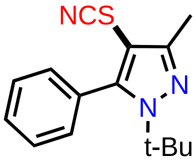

According to the procedure A, **2o** was purified by silica gel chromatography (10% EtOAc/PE). A yellow solid (173.68 mg, yield: 64%). mp: 99-100 °C. ^{1}H NMR (400 MHz, $CDCl_3$) δ 7.52 – 7.46 (m, 3H), 7.33 – 7.29 (m, 2H), 2.42 (s, 3H), 1.43 (s, 9H). ^{13}C NMR (101 MHz, $CDCl_3$) δ 149.1, 147.5, 131.2, 130.4, 129.6, 128.4, 111.6, 97.9, 62.7, 30.9, 12.1. HRMS (ESI) calcd for $C_{15}H_{17}N_3NaS^+$ [M + Na$^+$] 294.1035, found 294.1039.

1-Phenyl-4-thiocyanato-1*H*-pyrazole (2p)

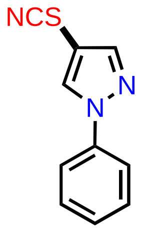

According to the procedure A, **2p** was purified by silica gel chromatography (5% EtOAc/PE). A white solid (175.0 mg, yield: 87%). mp: 61-62 °C. ^{1}H NMR (400 MHz, $CDCl_3$) δ 8.20 (s, 1H), 7.90 (s, 1H), 7.67 (d, *J* = 7.9 Hz, 2H), 7.50 (t, *J* = 7.9 Hz, 2H), 7.39 (t, *J* = 7.4 Hz, 1H). ^{13}C NMR (101 MHz, $CDCl_3$) δ 144.3, 139.2, 131.8, 129.7, 128.0, 119.6, 110.8, 100.0. HRMS (ESI) calcd for $C_{10}H_7N_3NaS^+$ [M + Na$^+$] 224.0253, found 224.0249.

3-Methyl-1-phenyl-4-thiocyanato-1*H*-pyrazol-5-ol (2q)

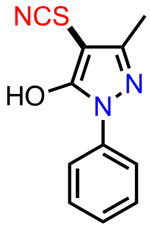

According to the procedure A, **2q** was purified by silica gel chromatography (1% MeOH/CH_2Cl_2). A white solid (170.46 mg, yield: 80%). mp: 110-112 °C. ^{1}H NMR (400 MHz, $CDCl_3$) δ 7.37 (d, J = 7.6 Hz, 2H), 7.28 (s, 1H), 7.22 (dd, J = 13.6, 6.6 Hz, 2H), 2.33 (s, 3H). ^{13}C NMR (101 MHz, $CDCl_3$) δ 160.3, 151.5, 135.0, 129.2, 127.5, 122.1, 110.6, 82.5, 11.6. HRMS (ESI) calcd for $C_{11}H_9N_3NaOS$+ [M + Na$^+$] 254.0359, found 254.0355.

The characterization data of **2q** agree with the literature values. [4]

3,5-Dimethyl-1-phenyl-4-((trifluoromethyl)thio)-1*H*-pyrazole (2r)

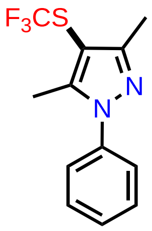

Following the reported procedure, [5] **2r** was purified by silica gel chromatography (10% EtOAc/PE). Colorless oil (171.54 mg, yield: 63%). ^{1}H NMR (400 MHz, $CDCl_3$) δ 7.51 – 7.38 (m, 5H), 2.40 (s, 3H), 2.39 (s, 3H). ^{13}C NMR (101 MHz, $CDCl_3$) δ 153.8, 145.9, 139.4, 129.5 (q, $J_{C\text{-}F}$ = 311.08 Hz), 129.3, 128.3, 124.9, 99.2, 11.9, 11.4. HRMS (ESI) calcd for $C_{12}H_{11}F_3N_2NaS^+$ [M + Na$^+$] 295.0487, found 295.0485.

The characterization data of **2r** agree with the literature values. [2]

3,5-Dimethyl-4-(methylthio)-1-phenyl-1*H*-pyrazole (2s)

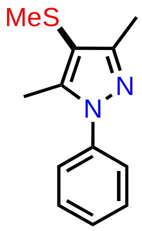

Following the reported procedure, [6] **2s** was purified by silica gel chromatography (10% EtOAc/PE). A white solid (122.25 mg, yield: 56%). mp: 95-96 °C. ^{1}H NMR (400 MHz, $CDCl_3$) δ 7.47 – 7.43 (m, 2H), 7.43 – 7.39 (m, 2H), 7.38 – 7.32 (m, 1H), 2.38 (s, 6H), 2.20 (s, 3H). ^{13}C NMR (101 MHz, $CDCl_3$) δ 152.2, 142.6, 139.9, 129.1, 127.6, 124.7, 110.9, 19.9, 12.2, 11.6. HRMS (ESI) calcd for $C_{12}H_{14}N_2NaS^+$ [M + Na$^+$] 241.0770, found 241.0773.

The characterization data of **2s** agree with the literature values. [2]

3,5-Dimethyl-1-phenyl -4-selenocyanato-1*H*-pyrazole (3a)

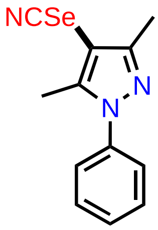

According to the procedure B, **3a** was purified by silica gel chromatography (15% EtOAc/PE). A white solid (226.5 mg, yield: 82%). mp: 82-84 °C. ^{1}H NMR (400 MHz, $CDCl_3$) δ 7.50 (t, *J* = 7.4 Hz, 2H), 7.46 – 7.37 (m, 3H), 2.45 (d, *J* = 6.1 Hz, 6H). ^{13}C NMR (101 MHz, $CDCl_3$) δ 152.4, 144.6, 139.2, 129.4, 128.6, 125.1, 100.9, 94.7, 12.9, 12.4. HRMS (ESI) calcd for $C_{12}H_{11}N_3NaSe^+$ [M + Na$^+$] 300.0010, found 300.0018. The characterization data of **3a** agree with the literature values. [7]

3,5-Dimethyl-4- selenocyanato-1-(*p*-tolyl)-1*H*-pyrazole (3b)

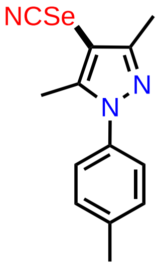

According to the procedure B, **3b** was purified by silica gel chromatography (15% EtOAc/PE). A white solid (252.5 mg, yield: 87%). mp: 77-79 °C. ^{1}H NMR (400 MHz, $CDCl_3$) δ 7.28 (s, 4H), 2.47 – 2.40 (m, 9H). ^{13}C NMR (101 MHz, $CDCl_3$) δ 152.1, 144.6, 138.7, 136.7, 129.9, 124.9, 101.0, 94.4, 21.2, 12.8, 12.3. HRMS (ESI) calcd for $C_{13}H_{13}N_3NaSe^+$ [M + Na$^+$] 314.0167, found 314.0170.

3,5-Dimethyl-4-selenocyanato-1-(*m*-tolyl)-1*H*-pyrazole (3c)

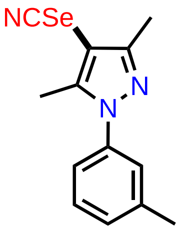

According to the procedure B, **3c** was purified by silica gel chromatography (15%

EtOAc/PE). Yellow oil (258.3 mg, yield: 89%). ^{1}H NMR (400 MHz, $CDCl_3$) δ 7.38 – 7.33 (m, 1H), 7.25 – 7.21 (m, 2H), 7.16 (d, *J* = 8.0 Hz, 1H), 2.44 (s, 3H), 2.43 (s, 3H), 2.41 (s, 3H). ^{13}C NMR (101 MHz, $CDCl_3$) δ 152.2, 144.5, 139.6, 139.1, 129.4, 129.0, 125.8, 122.0, 101.0, 94.5, 21.4, 12.9, 12.4. HRMS (ESI) calcd for $C_{13}H_{13}N_3NaSe^+$ [M + Na$^+$] 314.0167, found 314.0163.

3,5-Dimethyl-4-selenocyanato-1-(*o*-tolyl)-1*H*-pyrazole (3d)

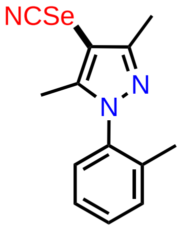

According to the procedure B, **3d** was purified by silica gel chromatography (15% EtOAc/PE). Yellow oil (261.2 mg, yield: 90%). ^{1}H NMR (400 MHz, $CDCl_3$) δ 7.41 – 7.27 (m, 3H), 7.19 (dd, *J* = 7.7, 1.0 Hz, 1H), 2.43 (s, 3H), 2.23 (s, 3H), 2.04 (s, 3H). ^{13}C NMR (101 MHz, $CDCl_3$) δ 152.1, 145.5, 138.0, 135.8, 131.2, 129.8, 127.5, 126.9, 101.1, 93.1, 17.2, 12.9, 11.4. HRMS (ESI) calcd for $C_{13}H_{13}N_3NaSe^+$ [M + Na$^+$] 314.0167, found 314.0162.

1-(4-Methoxyphenyl)-3,5-dimethyl-4-selenocyanato-1*H*-pyrazole (3e)

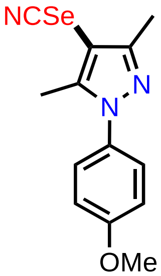

According to the procedure B, **3e** was purified by silica gel chromatography (15% EtOAc/PE). A white solid (225.0 mg, yield: 80%). mp: 82-85 °C. ^{1}H NMR (400 MHz, $CDCl_3$) δ 7.33 – 7.28 (m, 2H), 7.01 – 6.96 (m, 2H), 3.86 (s, 3H), 2.42 (s, 3H), 2.40 (s, 3H). ^{13}C NMR (101 MHz, $CDCl_3$) δ 159.6, 152.0, 144.7, 132.3, 126.6, 114.4, 101.0, 94.0, 55.6, 12.8, 12.2. HRMS (ESI) calcd for $C_{13}H_{13}N_3NaOSe^+$ [M + Na$^+$] 330.0116, found 330.0110.

1-(4-Fluorophenyl)-3,5-dimethyl-4-selenocyanato-1*H*-pyrazol (3f)

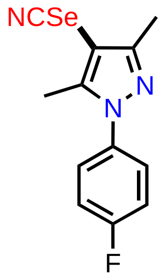

According to the procedure B, **3f** was purified by silica gel chromatography (15% EtOAc/PE). A white solid (232.4 mg, yield: 79%). mp: 75-77 °C. ^{1}H NMR (400 MHz, $CDCl_3$) δ 7.38 (ddt, J = 7.9, 5.3, 2.6 Hz, 2H), 7.22 – 7.15 (m, 2H), 2.43 (s, 6H). ^{13}C NMR (101 MHz, $CDCl_3$) δ 162.3 (d, $J_{C\text{-}F}$ = 250.2 Hz), 152.4, 144.7, 135.3 (d, $J_{C\text{-}F}$ = 3.2 Hz), 127.1 (d, $J_{C\text{-}F}$ = 8.8 Hz), 116.4 (d, $J_{C\text{-}F}$ = 23.1 Hz), 100.8, 94.8, 12.8, 12.3. HRMS (ESI) calcd for $C_{12}H_{10}FN_3NaSe^+$ [M + Na^+] 317.9916, found 317.9910.

1-(4-Chlorophenyl)-3,5-dimethyl-4-selenocyanato-1*H*-pyrazole (3g)

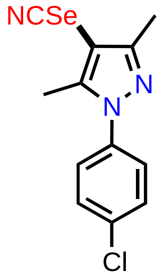

According to the procedure B, **3g** was purified by silica gel chromatography (15% EtOAc/PE). A white solid (279.6 mg, yield: 90%). mp: 95-98 °C. ^{1}H NMR (400 MHz, $CDCl_3$) δ 7.51 – 7.45 (m, 2H), 7.41 – 7.33 (m, 2H), 2.46 (s, 3H), 2.43 (s, 3H). ^{13}C NMR (101 MHz, $CDCl_3$) δ 152.7, 144.6, 137.7, 134.4, 129.6, 126.2, 100.7, 95.2, 12.9, 12.4. HRMS (ESI) calcd for $C_{12}H_{10}ClN_3NaSe^+$ [M + Na^+] 333.9621, found 323.9626.

1-(4-Bromophenyl)-3,5-dimethyl-4-selenocyanato-1*H*-pyrazole (3h)

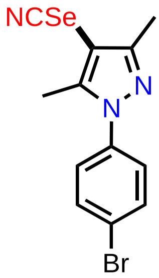

According to the procedure B, **3h** was purified by silica gel chromatography (15% EtOAc/PE). A white solid (269.9 mg, yield: 76%). mp: 96-98 °C. ^{1}H NMR (400 MHz, $CDCl_3$) δ 7.65 – 7.61 (m, 2H), 7.34 – 7.28 (m, 2H), 2.46 (s, 3H), 2.43 (s, 3H). ^{13}C NMR (101 MHz, $CDCl_3$) δ 152.7, 144.6, 138.2, 132.5, 126.4, 122.4, 100.7, 95.3, 12.9, 12.5. HRMS (ESI) calcd for $C_{12}H_{10}BrN_3NaSe^+$ [M + Na$^+$] 377.9116, found 377.9110.

1-(4-Iodophenyl)-3,5-dimethyl-4-selenocyanato-1*H*-pyrazole (3i)

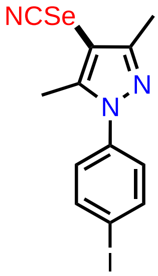

According to the procedure B, **3i** was purified by silica gel chromatography (15% EtOAc/PE). A white solid (301.6 mg, yield: 75%). mp: 85-87 °C. ^{1}H NMR (400 MHz, $CDCl_3$) δ 7.86 – 7.80 (m, 2H), 7.20 – 7.13 (m, 2H), 2.46 (s, 3H), 2.43 (s, 3H). ^{13}C NMR (101 MHz, $CDCl_3$) δ 152.7, 144.6, 138.9, 138.5, 126.6, 100.7, 95.4, 93.7, 12.9, 12.5. HRMS (ESI) calcd for $C_{12}H_{10}IN_3NaSe^+$ [M + Na$^+$] 425.8977, found 425.8974.

3,5-Dimethyl-4-selenocyanato-1-(4-(trifluoromethyl)phenyl)-1*H*-pyrazole (3j)

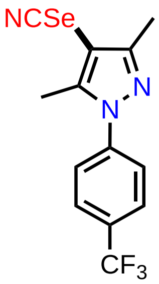

According to the procedure B, **3j** was purified by silica gel chromatography (15%

EtOAc/PE). A yellow solid (285.7 mg, yield: 83%). mp: 92-95 °C. ^{1}H NMR (400 MHz, $CDCl_3$) δ 7.77 (d, J = 8.3 Hz, 2H), 7.58 (d, J = 8.2 Hz, 2H), 2.52 (s, 3H), 2.44 (s, 3H). ^{13}C NMR (101 MHz, $CDCl_3$) δ 153.1, 144.7, 142.0, 130.4 (q, $J_{C\text{-}F}$ = 33.1 Hz), 126.6 (q, $J_{C\text{-}F}$ = 3.7 Hz), 124.94 (q, $J_{C\text{-}F}$ = 273.3 Hz), 124.9, 100.6, 96.1, 12.9, 12.7. HRMS (ESI) calcd for $C_{13}H_{10}F_3N_3NaSe^+$ [M + Na$^+$] 367.9884, found 367.9880.

3,5-Dimethyl-1-(4-nitrophenyl)-4-selenocyanato-1*H*-pyrazole (3k)

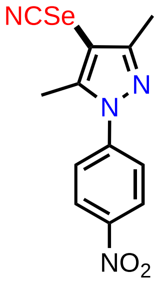

According to the procedure B, **3k** was purified by silica gel chromatography (15% EtOAc/PE). A white solid (183.1 mg, yield: 57%). mp: 140-142 °C. ^{1}H NMR (400 MHz, $CDCl_3$) δ 8.41 – 8.36 (m, 2H), 7.69 – 7.65 (m, 2H), 2.58 (s, 3H), 2.46 (s, 3H). ^{13}C NMR (101 MHz, $CDCl_3$) δ 153.7, 146.8, 144.9, 144.0, 124.9, 124.7, 100.3, 97.3, 13.0, 12.9. HRMS (ESI) calcd for $C_{12}H_{10}N_4NaO_2Se^+$ [M + Na$^+$] 344.9861, found 344.9868.

1-(3,5-Dichlorophenyl)-3,5-dimethyl-4-selenocyanato-1*H*-pyrazole (3l)

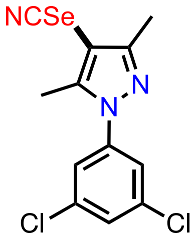

According to the procedure B, **3l** was purified by silica gel chromatography (15% EtOAc/PE). A yellow solid (296.8 mg, yield: 82%). mp: 104-106 °C. ^{1}H NMR (400 MHz, $CDCl_3$) δ 7.42 (t, J = 1.8 Hz, 1H), 7.37 (d, J = 1.8 Hz, 2H), 2.51 (s, 3H), 2.42 (s, 3H). ^{13}C NMR (101 MHz, $CDCl_3$) δ 153.2, 144.7, 140.7, 135.7, 128.5, 123.3, 100.4, 96.2, 12.9, 12.7. HRMS (ESI) calcd for $C_{12}H_9Cl_2N_3NaSe^+$ [M + Na$^+$] 367.9231, found 367.9235.

2-(3,5-Dimethyl-4-selenocyanato-1*H*-pyrazol-1-yl)pyrazine (3m)

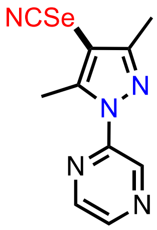

According to the procedure B, **3m** was purified by silica gel chromatography (15% EtOAc/PE). A white solid (239.2 mg, yield: 86%). mp: 95-98 °C. ^{1}H NMR (400 MHz, $CDCl_3$) δ 9.25 (d, *J* = 1.2 Hz, 1H), 8.52 (d, *J* = 2.5 Hz, 1H), 8.40 (dd, *J* = 2.4, 1.5 Hz, 1H), 2.83 (s, 3H), 2.47 (s, 3H). ^{13}C NMR (101 MHz, $CDCl_3$) δ 153.7, 149.0, 147.0, 142.1, 141.2, 138.6, 100.3, 98.4, 14.3, 13.1. HRMS (ESI) calcd for $C_{10}H_9N_5NaSe^+$ [M + Na^+] 301.9915, found 301.9918.

1,3,5-Triphenyl-4-selenocyanato-1*H*-pyrazole (3n)

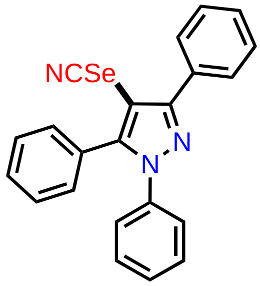

According to the procedure B, **3n** was purified by silica gel chromatography (15% EtOAc/PE). A white solid (168.1 mg, yield: 42%). mp: 99-100 °C. ^{1}H NMR (400 MHz, $CDCl_3$) δ 7.97 – 7.92 (m, 2H), 7.56 – 7.41 (m, 6H), 7.40 – 7.28 (m, 7H). ^{13}C NMR (101 MHz, $CDCl_3$) δ 154.9, 149.0, 139.3, 131.5, 130.4, 129.7, 129.1, 129.0, 128.8, 128.8, 128.6, 128.2, 125.0, 102.0, 94.3. HRMS (ESI) calcd for $C_{22}H_{15}N_3NaSe^+$ [M + Na^+] 424.0323, found 424.0326.

1-(*tert*-Butyl)-3-methyl-5-phenyl-4-selenocyanato-1*H*-pyrazole (3o)

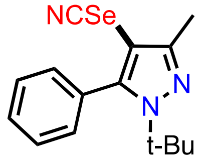

According to the procedure B, **3o** was purified by silica gel chromatography (15%

EtOAc/PE). A white solid (206.9 mg, yield: 65%). mp: 97-99 °C. ^{1}H NMR (400 MHz, $CDCl_3$) δ 7.50 – 7.45 (m, 3H), 7.32 – 7.26 (m, 2H), 2.42 (s, 3H), 1.43 (s, 9H). ^{13}C NMR (101 MHz, $CDCl_3$) δ 149.3, 147.8, 132.1, 130.5, 129.4, 128.3, 101.8, 96.2, 62.5, 31.0, 12.9. HRMS (ESI) calcd for $C_{15}H_{17}N_3NaSe^+$ [M + Na$^+$] 342.0480, found 342.0485.

1-Phenyl-4-selenocyanato-1*H*-pyrazole (3p)

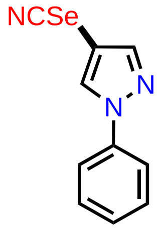

According to the procedure B, **3p** was purified by silica gel chromatography (10% EtOAc/PE). A white solid (196.0 mg, yield: 79%). mp: 62-63 °C. ^{1}H NMR (400 MHz, $CDCl_3$) δ 8.20 (s, 1H), 7.90 (s, 1H), 7.68 (d, J = 7.7 Hz, 2H), 7.50 (t, J = 7.9 Hz, 2H), 7.38 (t, J = 7.4 Hz, 1H). ^{13}C NMR (101 MHz, $CDCl_3$) δ 145.5, 139.2, 132.9, 129.7, 127.9, 119.7, 100.9, 94.7. HRMS (ESI) calcd for $C_{10}H_7N_3NaSe^+$ [M + Na$^+$] 271.9697, found 271.9700.

3,5-Dimethyl-1-phenyl-4-((trifluoromethyl)selanyl)-1*H*-pyrazole (3q)

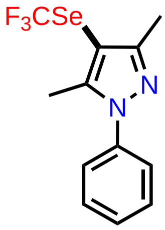

Following the reported procedure, [5] **3q** was purified by silica gel chromatography (20% EtOAc/PE). A white solid (191.5 mg, yield: 60%). mp: 89-91 °C. ^{1}H NMR (400 MHz, $CDCl_3$) δ 7.53 – 7.37 (m, 5H), 2.43 (s, 3H), 2.41 (s, 3H). ^{13}C NMR (151 MHz, $CDCl_3$) δ 153.6, 145.5, 139.6, 129.2, 128.2, 123.5, 112.4 (q, J = 335.2 Hz), 97.8, 12.7, 12.3. HRMS (ESI) calcd for $C_{12}H_{11}F_3N_2NaSe^+$ [M + Na$^+$] 342.9932, found 342.9936.

3,5-Dimethyl-4-(methylselanyl)-1-phenyl-1*H*-pyrazole (3r)

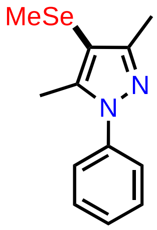

Following the reported procedure, [6] **3r** was purified by silica gel chromatography (20% EtOAc/PE). A white solid (164.4 mg, yield: 62%). mp: 100-102 °C. ^{1}H NMR (400 MHz, $CDCl_3$) δ 7.46 (dd, J = 8.2, 6.8 Hz, 2H), 7.42 (dd, J = 5.6, 3.0 Hz, 2H), 7.39 – 7.33 (m, 1H), 2.41 (s, 3H), 2.40 (s, 3H), 2.06 (s, 3H). ^{13}C NMR (101 MHz, $CDCl_3$) δ 152.5, 142.9, 139.9, 129.1, 127.7, 124.8, 104.3, 13.0, 12.5, 8.8. HRMS (ESI) calcd for $C_{12}H_{14}N_2NaSe^+$ [M + Na$^+$] 289.0214, found 289.0218.

The characterization data of **3r** agree with the literature values. [8]

IV. References

[1] Reddy, C. S., Devi, M. V., Sunitha, M., Nagaraj, A. *Chem. Pharm. Bull.* **2010**, *58*, 1622-1626.

[2] He, W. B., Zhao, S. J., Chen, J. Y., Jiang, J., Chen, X., Xu, X., He, W. M. *Chin. Chem. Lett.* **2023**, *34*, 107640.

[3] Songsichan, T., Katrun, P., Khaikate, O., Soorukram, D., Pohmakotr, M., Reutrakul, V., Kuhakarn, C. *SynOpen* **2018**, *02*, 0006-0016.

[4] Pan, J., Liu, C., Wang, J., Dai, Y., Wang, S., Guo, C. *Tetrahedron Lett.* **2021**, *77*, 153253.

[5] Jouvin, K., Matheis, C., Goossen, L. J. *Chem. Eur. J.* **2015**, *21*, 14324-14327.

[6] Adams, R., Bramlet, H. B., Tendick, F. H. *J. Am. Chem. Soc.* **1920**, *42*, 2369-2374.

[7] Ali, D., Mondal, N., Panday, A. K., Choudhury, L. H. *ACS Omega* **2023**, *8*, 25349-25357.

[8] Wu, Y., Chen, J. Y., Ning, J., Jiang, X., Deng, J., Deng, Y., Xu, R., He, W. M. *Green Chem.* **2021**, *23*, 3950-3954.

V. NMR Spectra of products

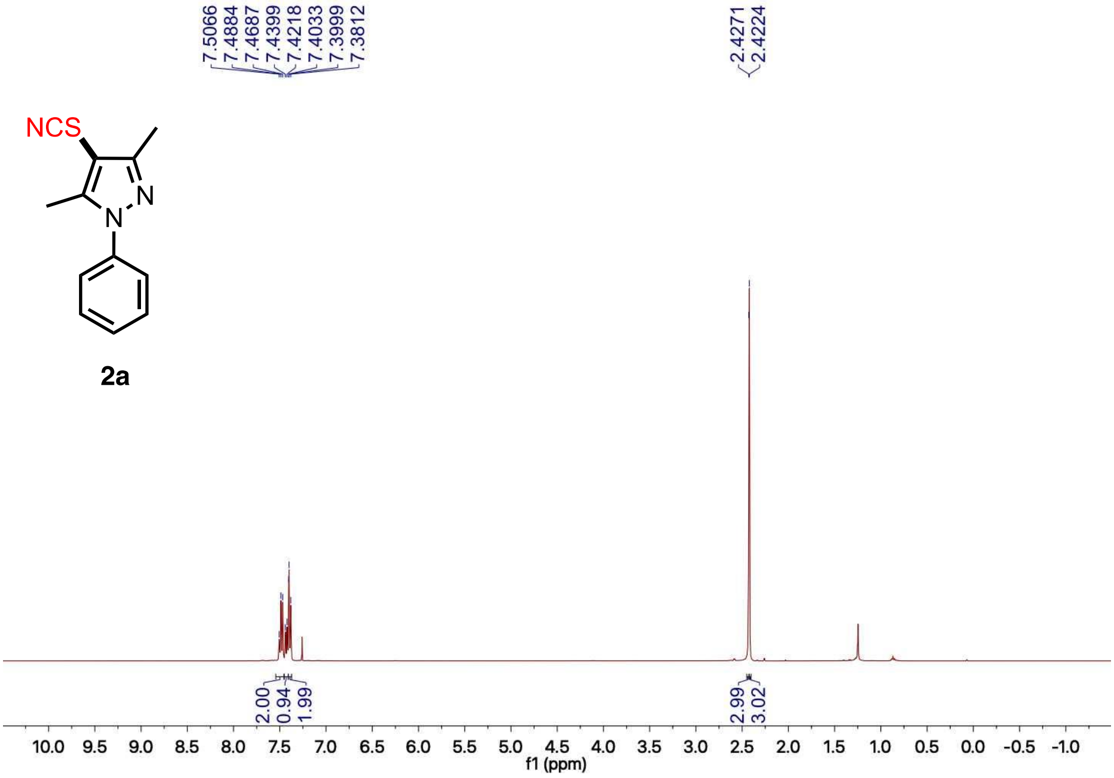

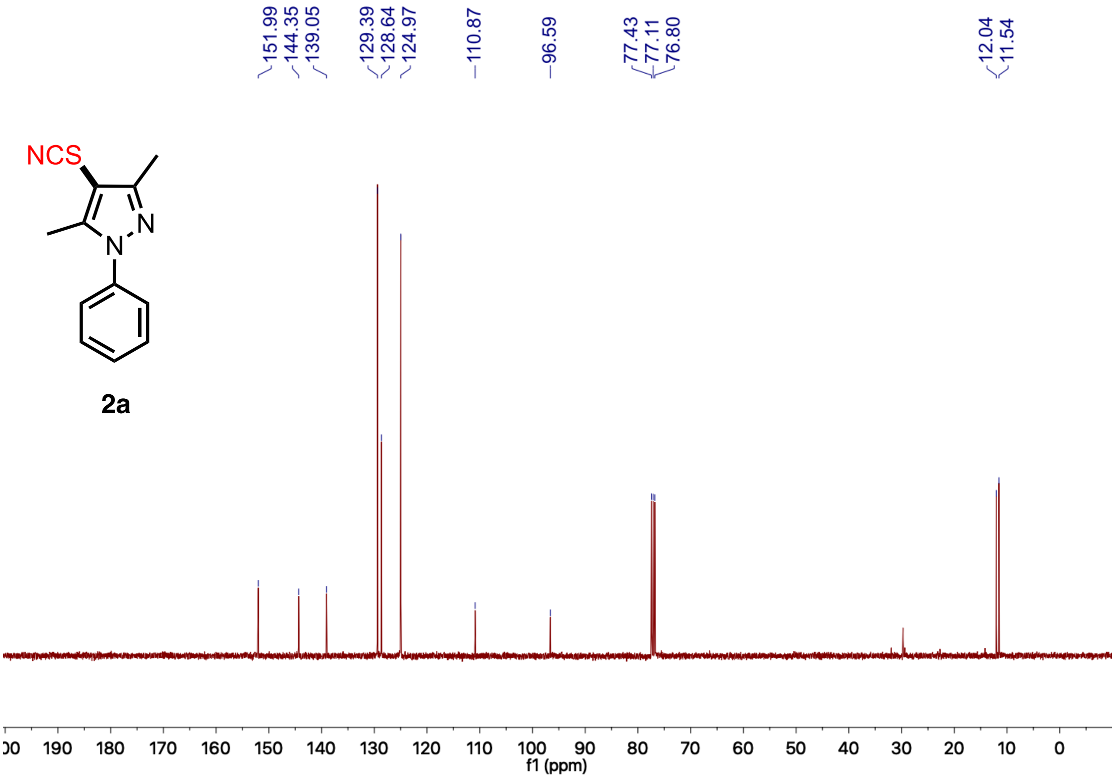

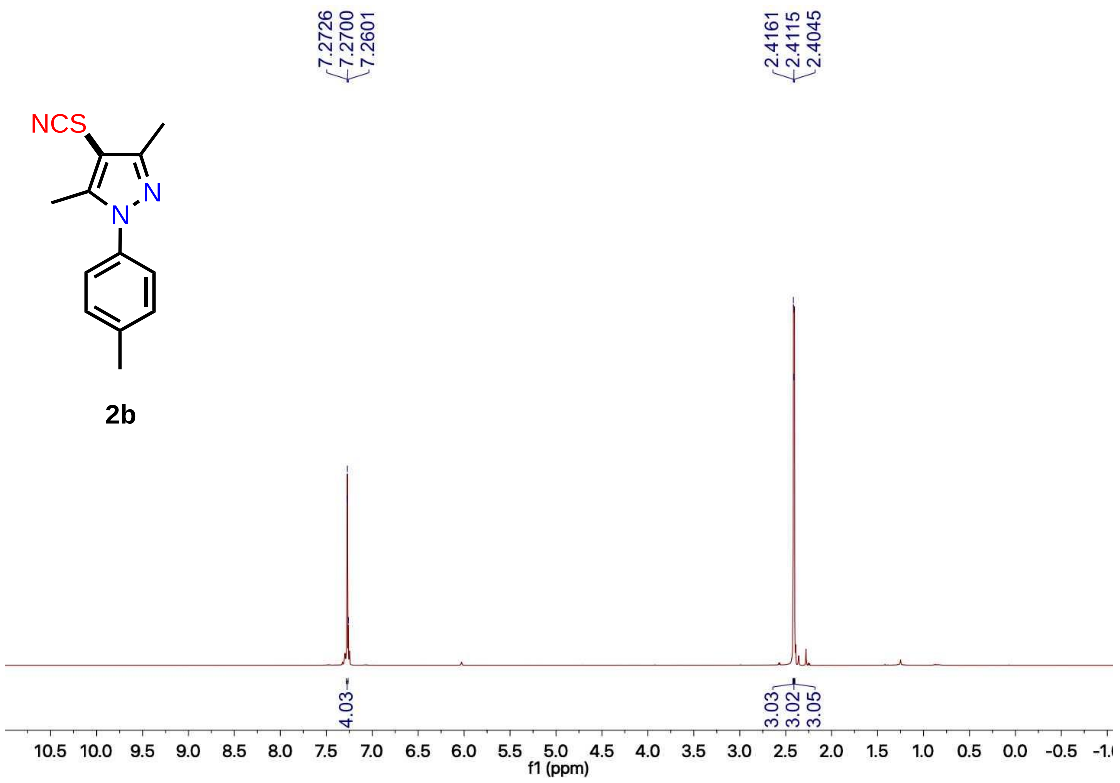
7.2726
7.2700
7.2601
2.4161
2.4115
2.4045
NCS
N
N
2b
4.03
3.03
3.02
3.05
10.5 10.0 9.5 9.0 8.5 8.0 7.5 7.0 6.5 6.0 5.5 5.0 4.5 4.0 3.5 3.0 2.5 2.0 1.5 1.0 0.5 0.0 -0.5
f1 (ppm)

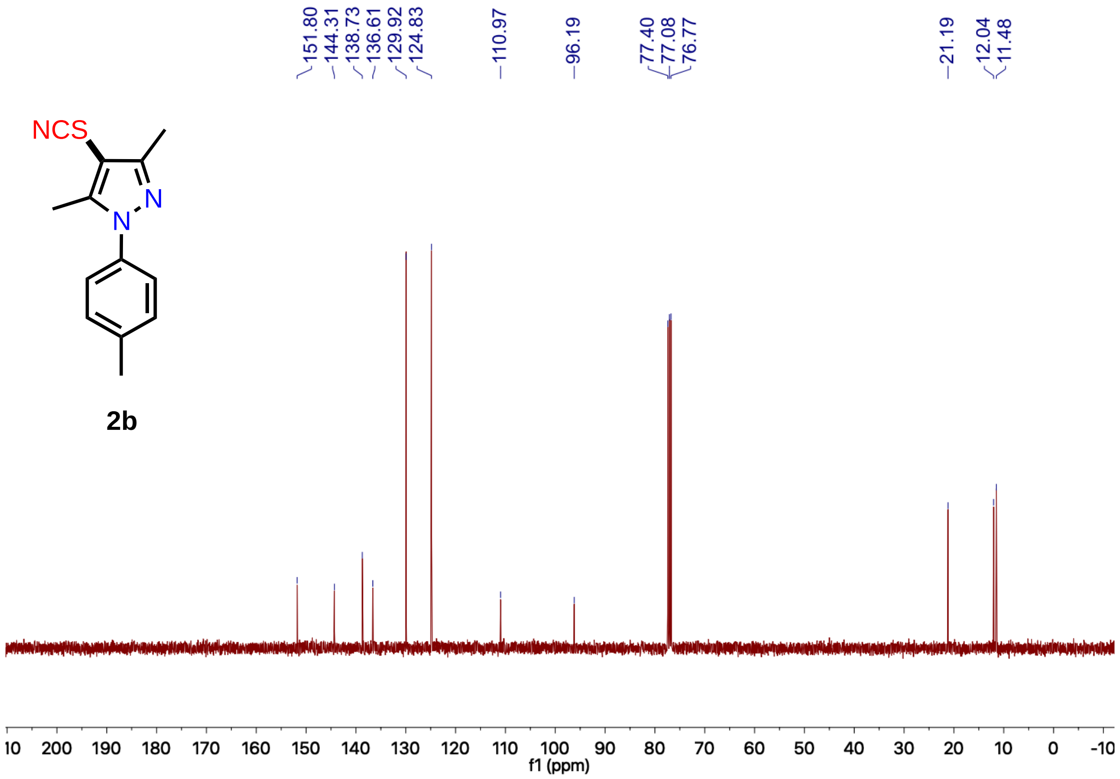
151.80
144.31
138.73
136.61
129.92
124.83
110.97
96.19
77.40
77.08
76.77
21.19
12.04
11.48
NCS
N
N
2b
200 190 180 170 160 150 140 130 120 110 100 90 80 70 60 50 40 30 20 10 0 -10
f1 (ppm)

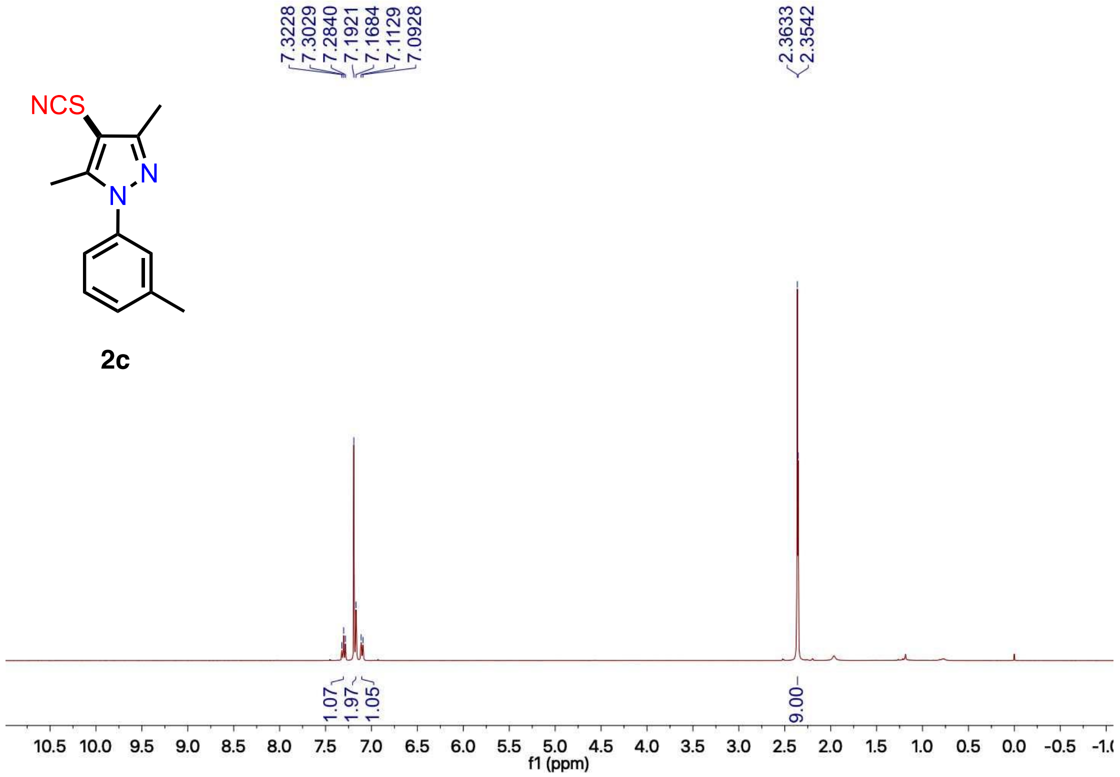
7.3228
7.3029
7.2840
7.1921
7.1684
7.1129
7.0928
2.3633
2.3542
NCS
N
N
2c
1.07
1.97
1.05
9.00
10.5 10.0 9.5 9.0 8.5 8.0 7.5 7.0 6.5 6.0 5.5 5.0 4.5 4.0 3.5 3.0 2.5 2.0 1.5 1.0 0.5 0.0 -0.5
f1 (ppm)

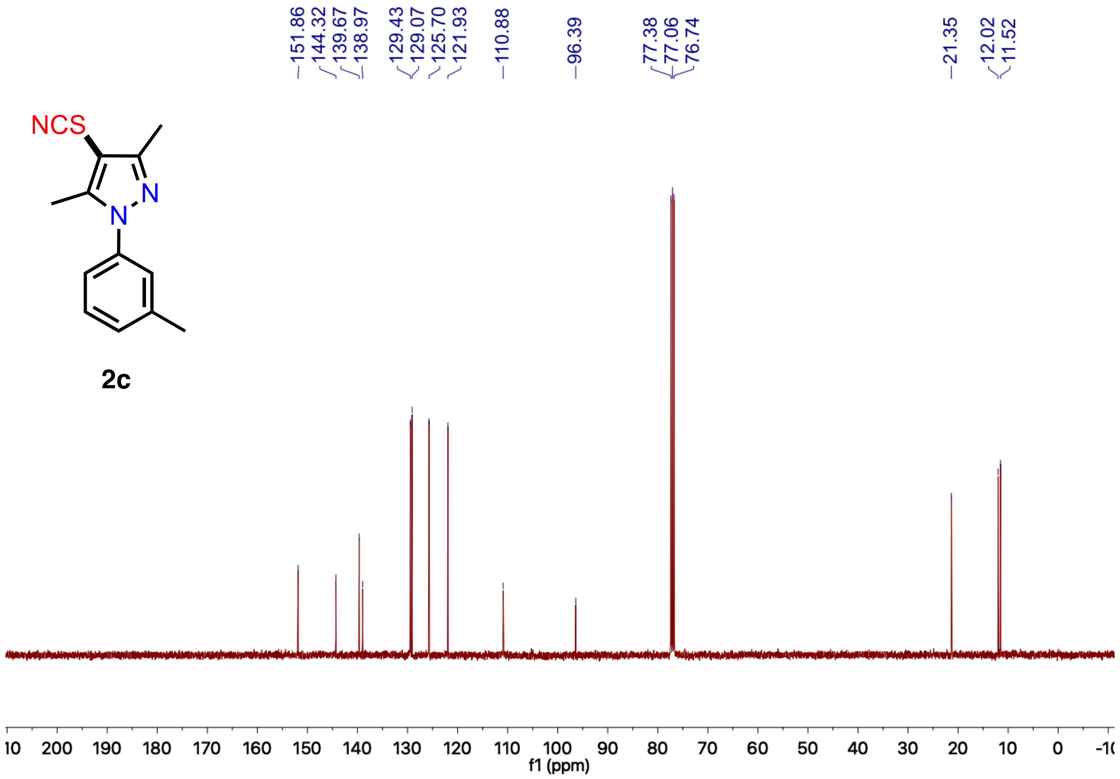
151.86
144.32
139.67
138.97
129.43
129.07
125.70
121.93
110.88
96.39
77.38
77.06
76.74
21.35
12.02
11.52
NCS
N
N
2c
200 190 180 170 160 150 140 130 120 110 100 90 80 70 60 50 40 30 20 10 0
f1 (ppm)

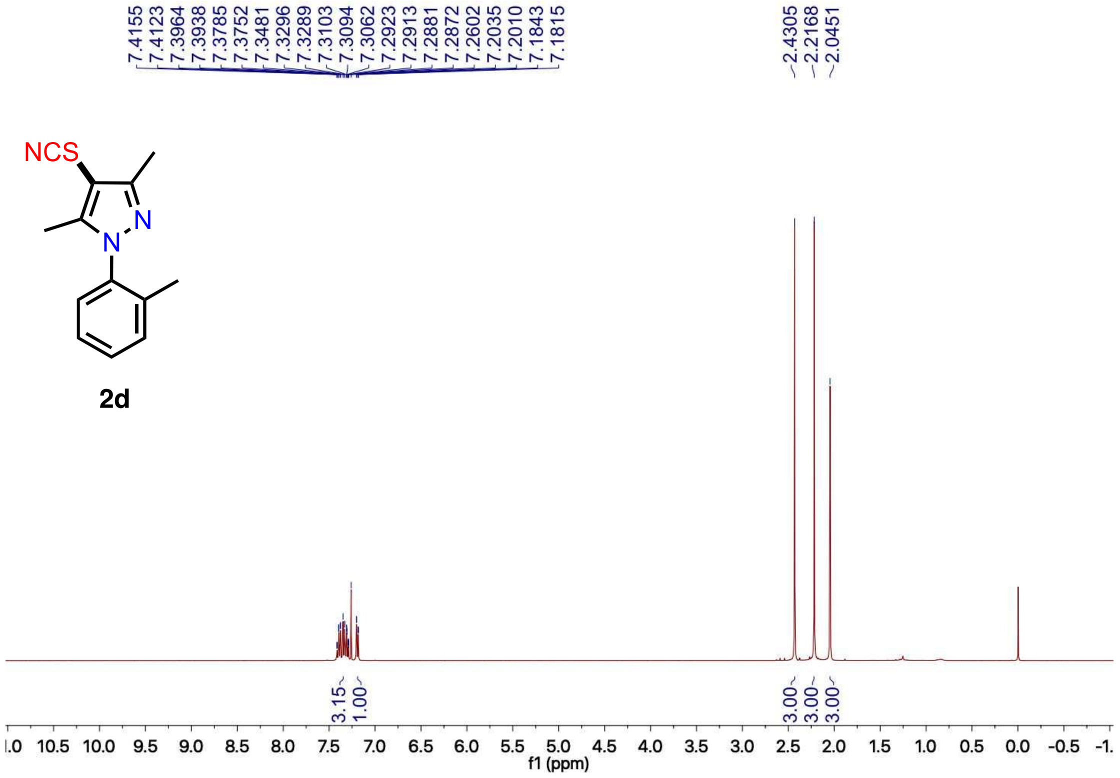
7.4155
7.4123
7.3964
7.3938
7.3785
7.3752
7.3481
7.3296
7.3289
7.3103
7.3094
7.3062
7.2923
7.2913
7.2881
7.2872
7.2602
7.2035
7.2010
7.1843
7.1815
2.4305
2.2168
2.0451
NCS
N
N
2d
3.15
1.00
3.00
3.00
3.00
f1 (ppm)

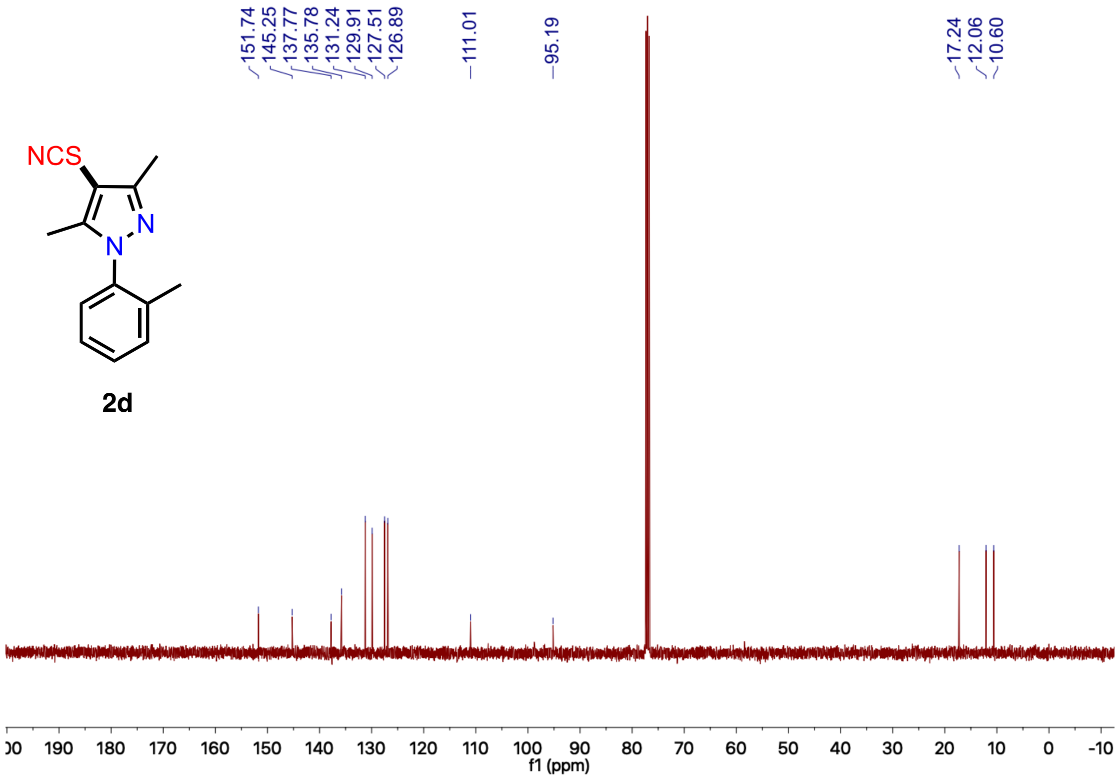
151.74
145.25
137.77
135.78
131.24
129.91
127.51
126.89
111.01
95.19
17.24
12.06
10.60
NCS
N
N
2d
f1 (ppm)

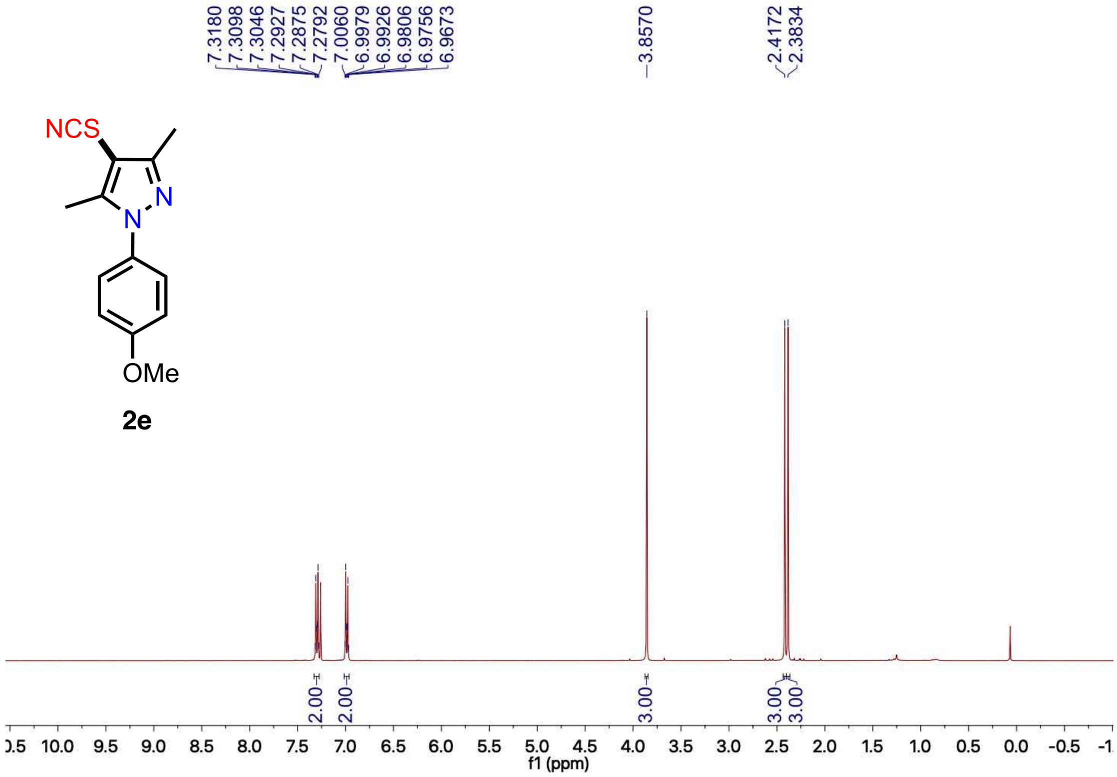
7.3180
7.3098
7.3046
7.2927
7.2875
7.2792
7.0060
6.9979
6.9926
6.9806
6.9756
6.9673
3.8570
2.4172
2.3834
NCS
N
N
OMe
2e
2.00
2.00
3.00
3.00
3.00
f1 (ppm)

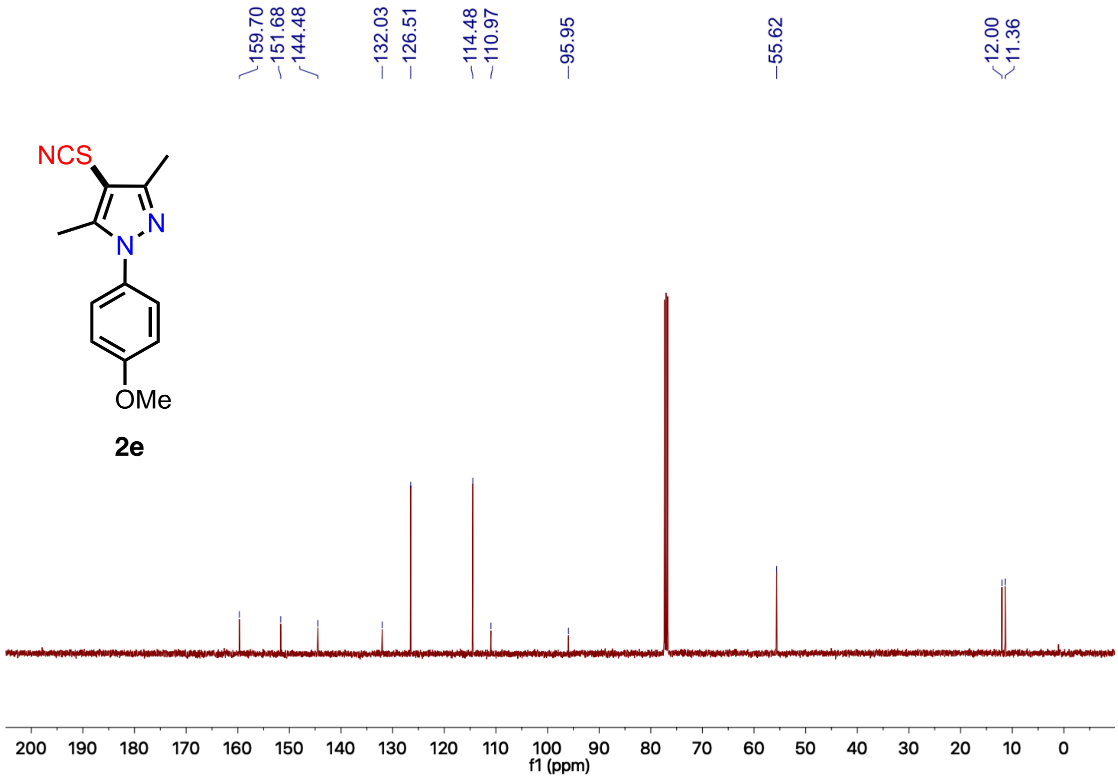
159.70
151.68
144.48
132.03
126.51
114.48
110.97
95.95
55.62
12.00
11.36
NCS
N
N
OMe
2e
f1 (ppm)

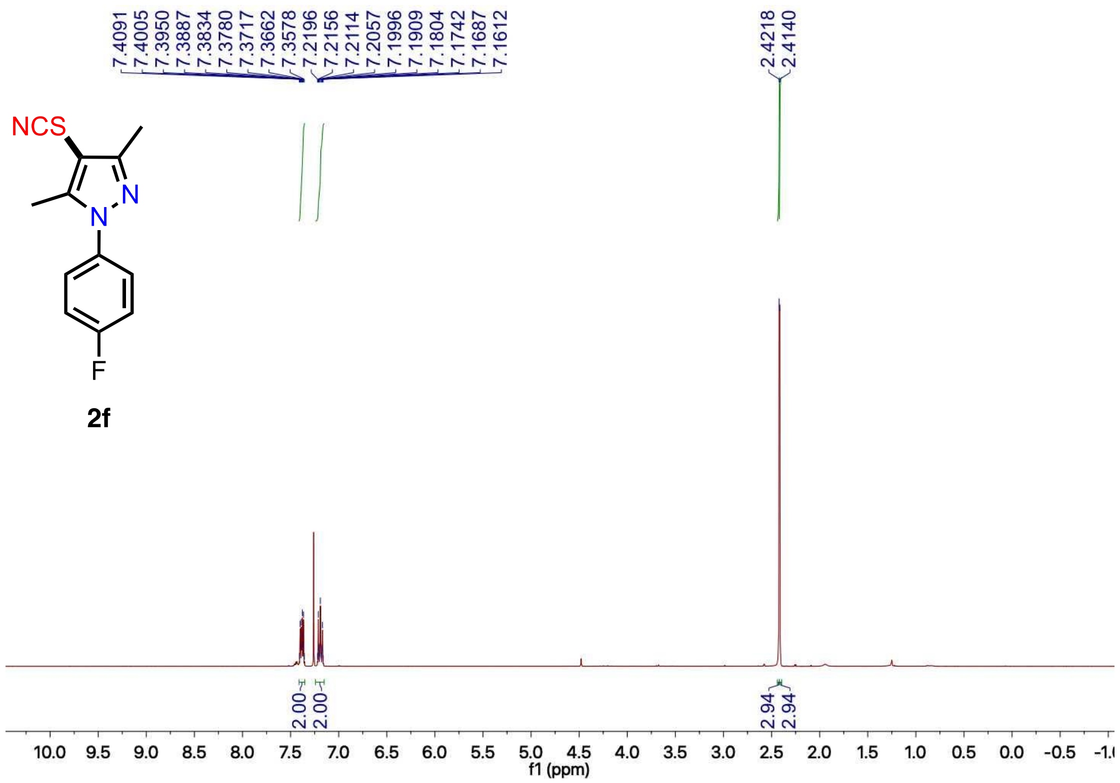
NCS
N
N
F
2f
7.4091
7.4005
7.3950
7.3887
7.3834
7.3780
7.3717
7.3662
7.3578
7.2196
7.2156
7.2114
7.2057
7.1996
7.1909
7.1804
7.1742
7.1687
7.1612
2.4218
2.4140
2.00
2.00
2.94
2.94
f1 (ppm)

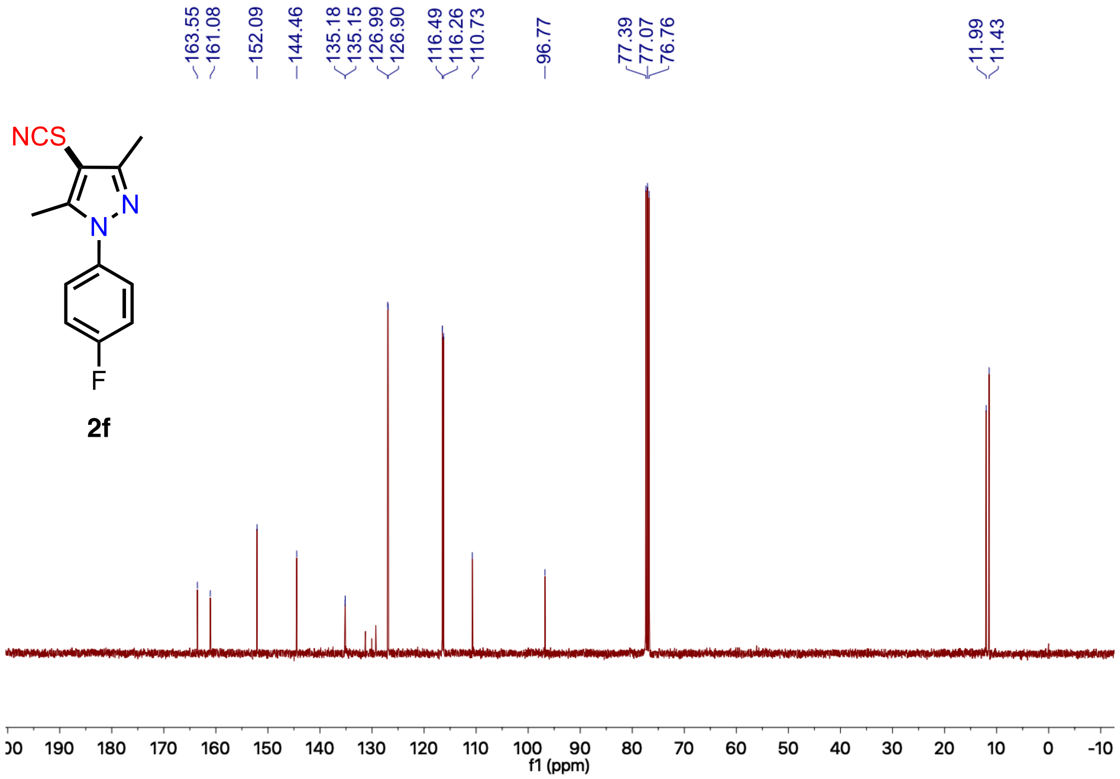
NCS
N
N
F
2f
163.55
161.08
152.09
144.46
135.18
135.15
126.99
126.90
116.49
116.26
110.73
96.77
77.39
77.07
76.76
11.99
11.43
f1 (ppm)

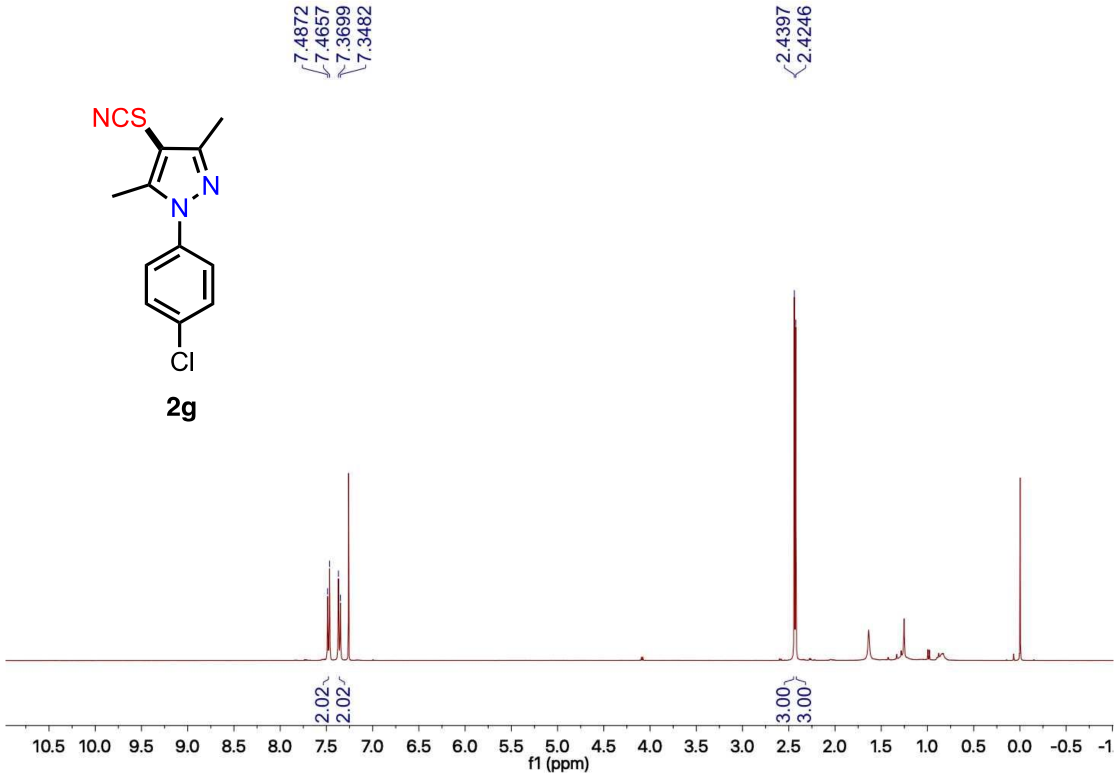
7.4872
7.4657
7.3699
7.3482
2.4397
2.4246
NCS
N
N
Cl
2g
2.02
2.02
3.00
3.00
10.5 10.0 9.5 9.0 8.5 8.0 7.5 7.0 6.5 6.0 5.5 5.0 4.5 4.0 3.5 3.0 2.5 2.0 1.5 1.0 0.5 0.0 -0.5
f1 (ppm)

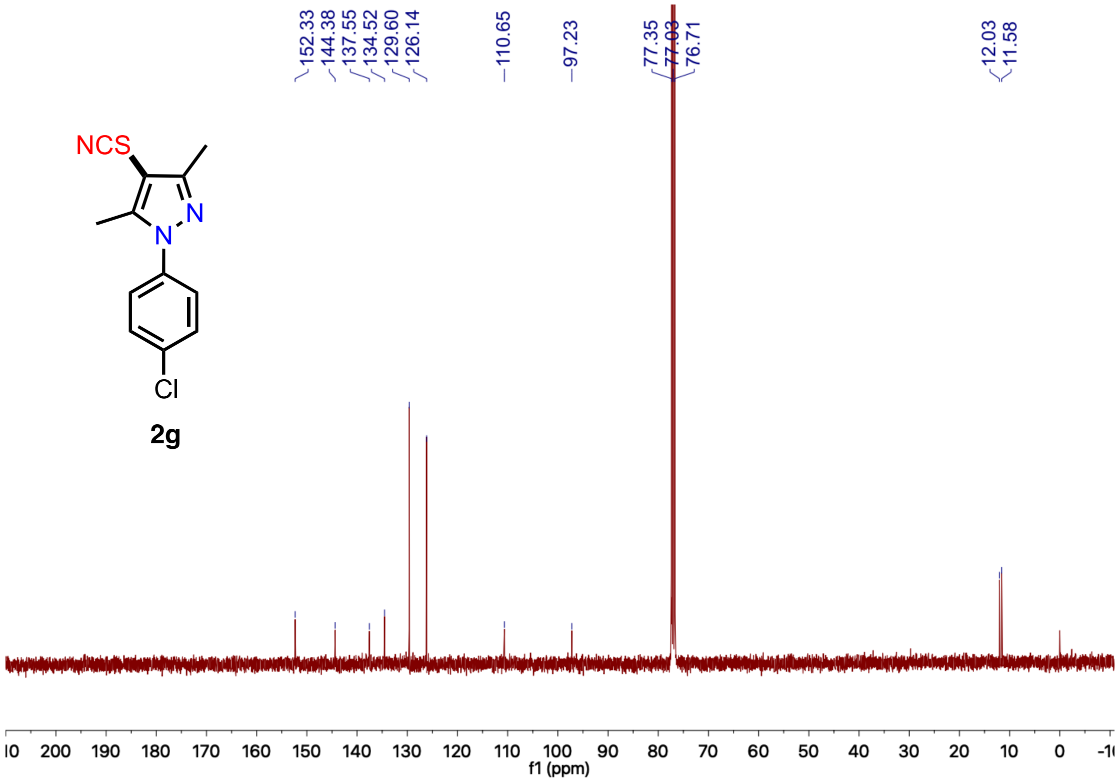
152.33
144.38
137.55
134.52
129.60
126.14
110.65
97.23
77.35
77.03
76.71
12.03
11.58
NCS
N
N
Cl
2g
200 190 180 170 160 150 140 130 120 110 100 90 80 70 60 50 40 30 20 10 0
f1 (ppm)

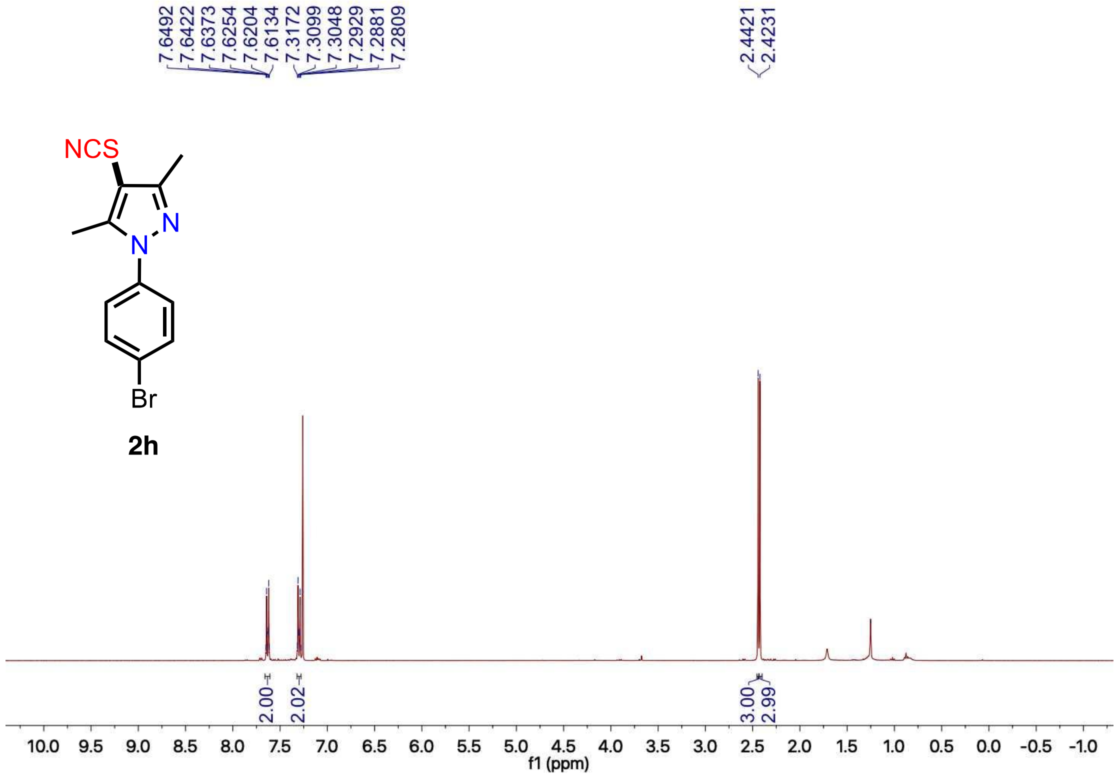
7.6492
7.6422
7.6373
7.6254
7.6204
7.6134
7.3172
7.3099
7.3048
7.2929
7.2881
7.2809
2.4421
2.4231
NCS
N
N
Br
2h
2.00
2.02
3.00
2.99
f1 (ppm)

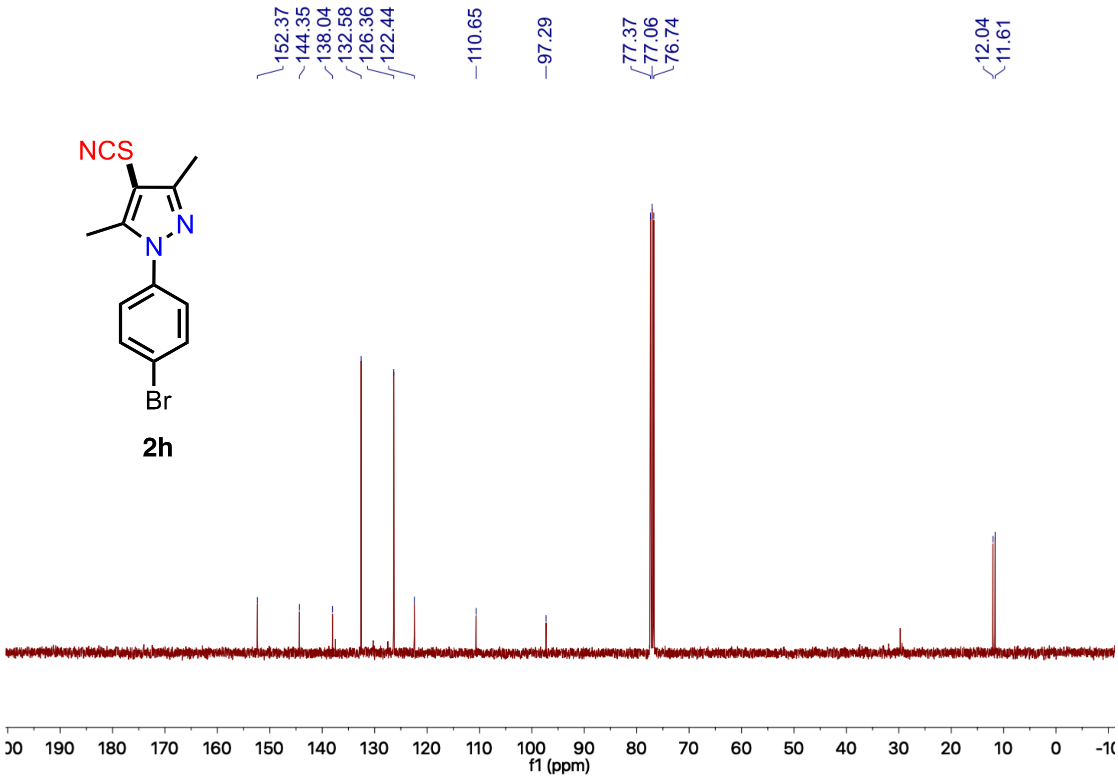
152.37
144.35
138.04
132.58
126.36
122.44
110.65
97.29
77.37
77.06
76.74
12.04
11.61
NCS
N
N
Br
2h
f1 (ppm)

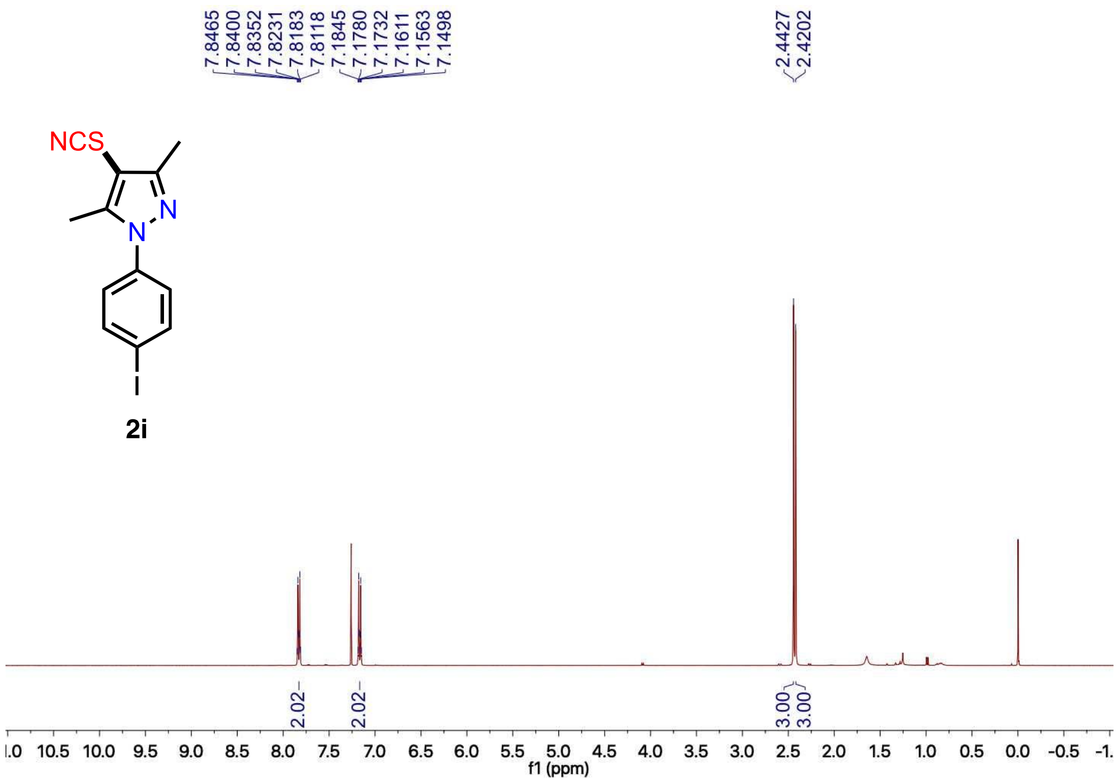
7.8465
7.8400
7.8352
7.8231
7.8183
7.8118
7.1845
7.1780
7.1732
7.1611
7.1563
7.1498
2.4427
2.4202
NCS
N
N
2i
2.02
2.02
3.00
3.00
f1 (ppm)

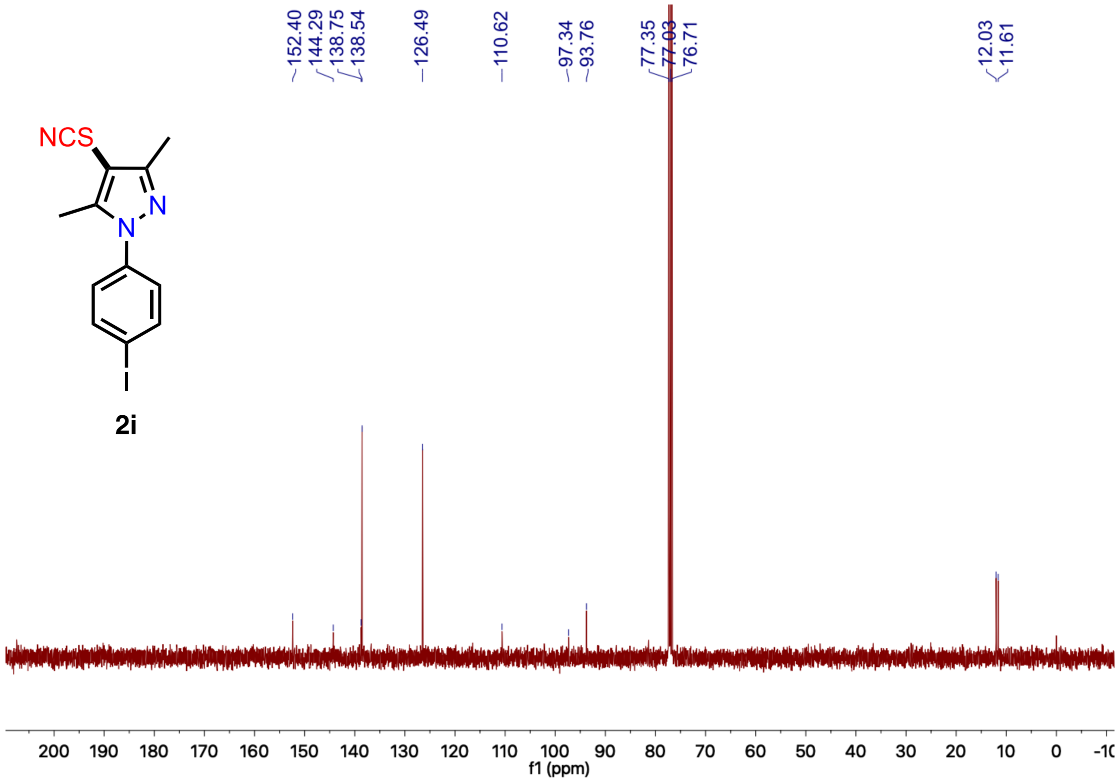
152.40
144.29
138.75
138.54
126.49
110.62
97.34
93.76
77.35
77.03
76.71
12.03
11.61
NCS
N
N
2i
f1 (ppm)

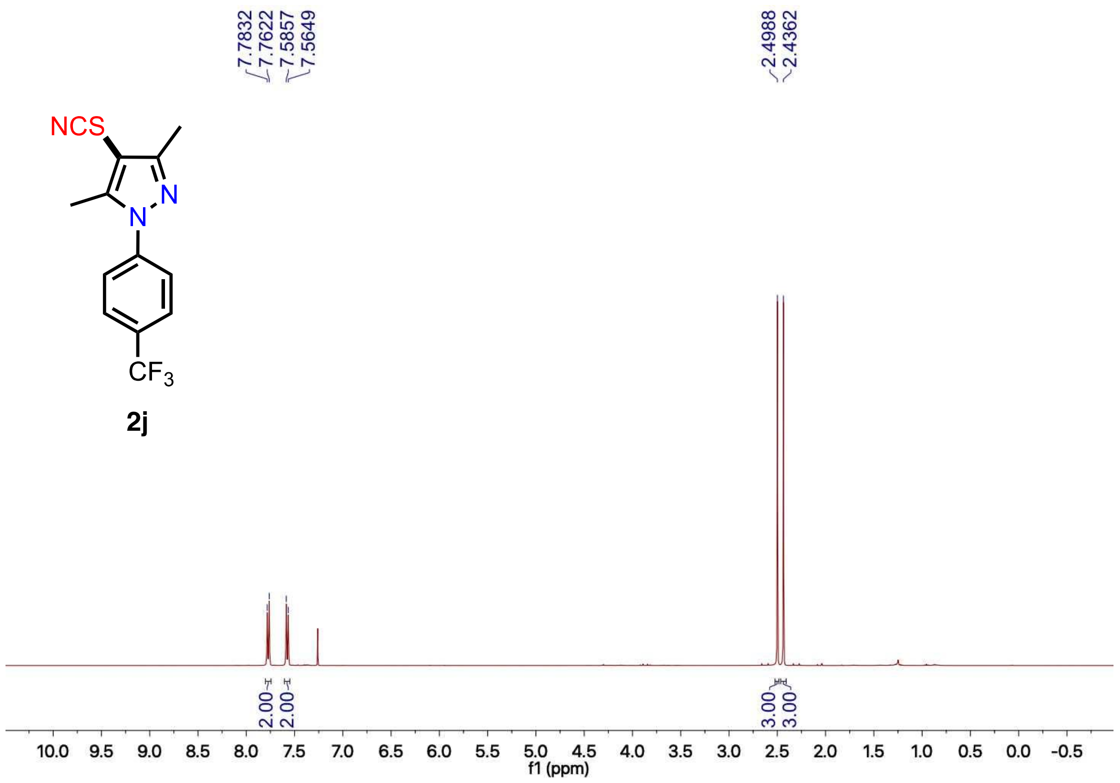
7.7832
7.7622
7.5857
7.5649
2.4988
2.4362
NCS
N
N
CF3
2j
2.00
2.00
3.00
3.00
10.0 9.5 9.0 8.5 8.0 7.5 7.0 6.5 6.0 5.5 5.0 4.5 4.0 3.5 3.0 2.5 2.0 1.5 1.0 0.5 0.0 -0.5
f1 (ppm)

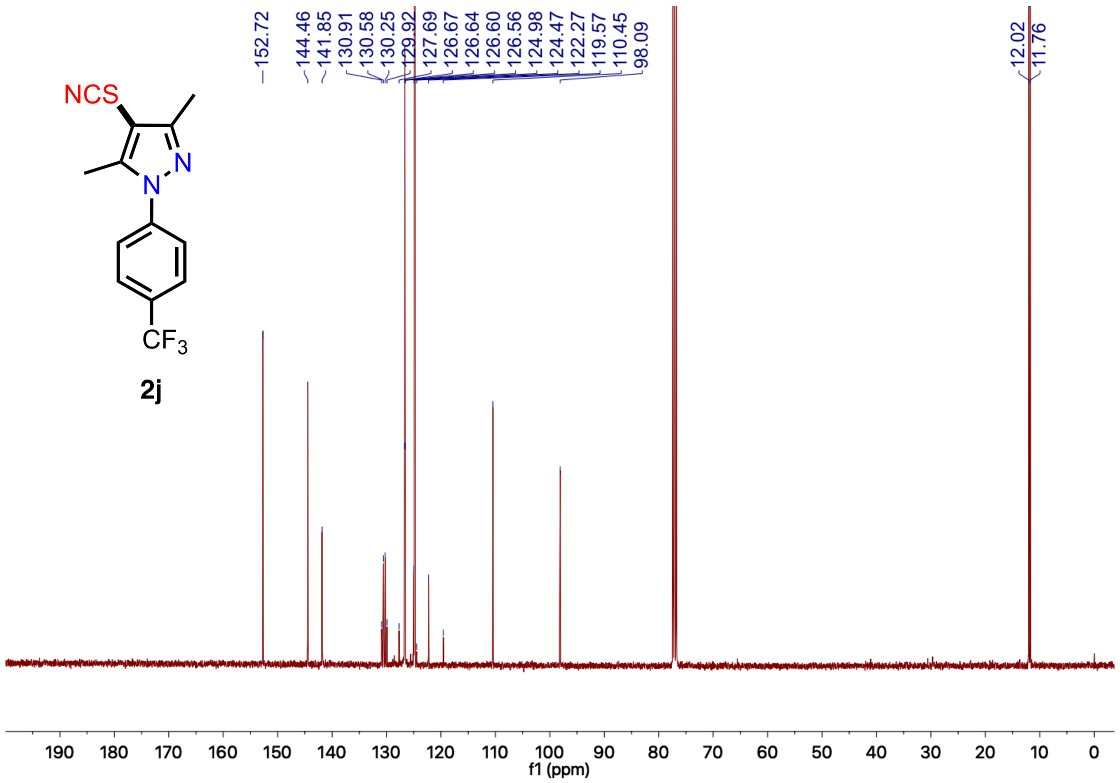
152.72
144.46
141.85
130.91
130.58
130.25
129.92
127.69
126.67
126.64
126.60
126.56
124.98
124.47
122.27
119.57
110.45
98.09
12.02
11.76
NCS
N
N
CF3
2j
190 180 170 160 150 140 130 120 110 100 90 80 70 60 50 40 30 20 10 0
f1 (ppm)

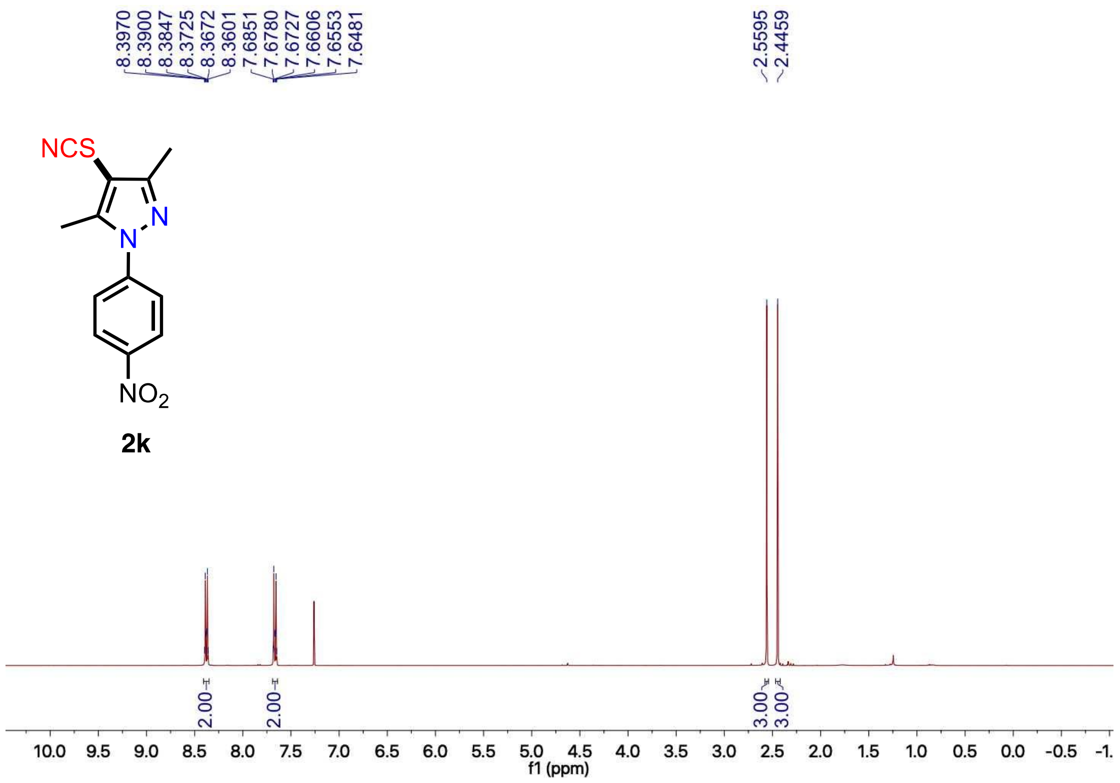

8.3970
8.3900
8.3847
8.3725
8.3672
8.3601
7.6851
7.6780
7.6727
7.6606
7.6553
7.6481
2.5595
2.4459
NCS
N
N
NO2
2k
2.00
2.00
3.00
3.00
10.0 9.5 9.0 8.5 8.0 7.5 7.0 6.5 6.0 5.5 5.0 4.5 4.0 3.5 3.0 2.5 2.0 1.5 1.0 0.5 0.0 -0.5 -1.
f1 (ppm)

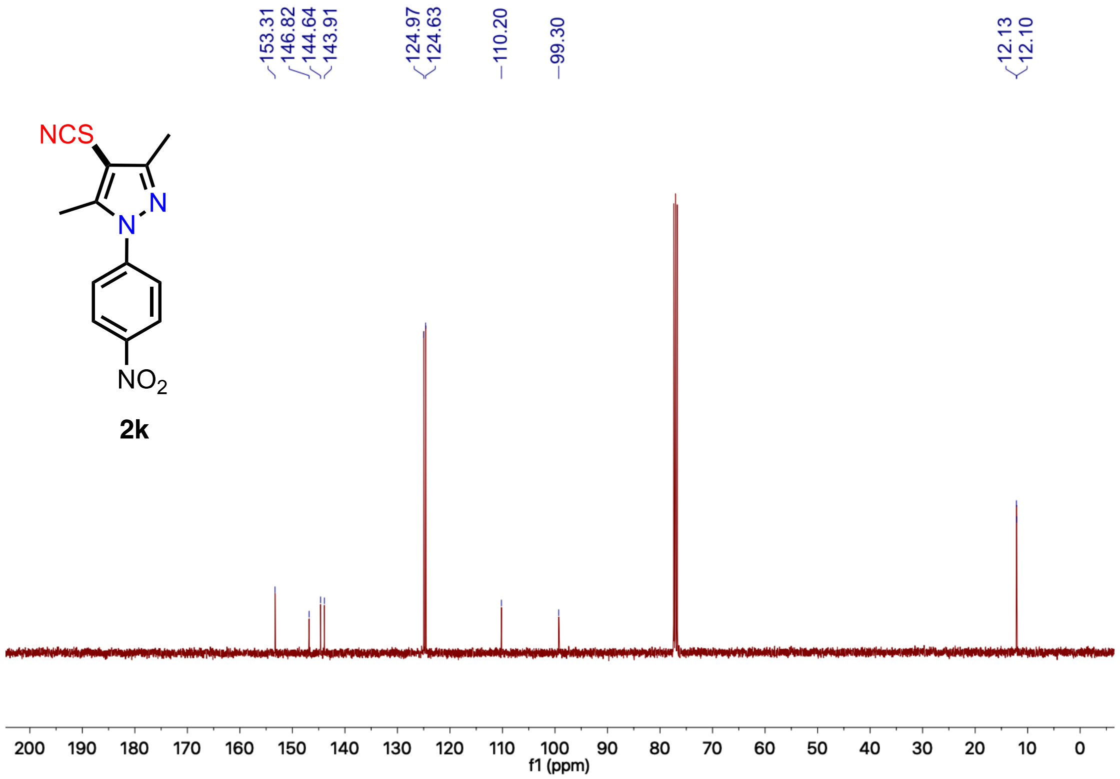

153.31
146.82
144.64
143.91
124.97
124.63
110.20
99.30
12.13
12.10
NCS
N
N
NO2
2k
200 190 180 170 160 150 140 130 120 110 100 90 80 70 60 50 40 30 20 10 0
f1 (ppm)

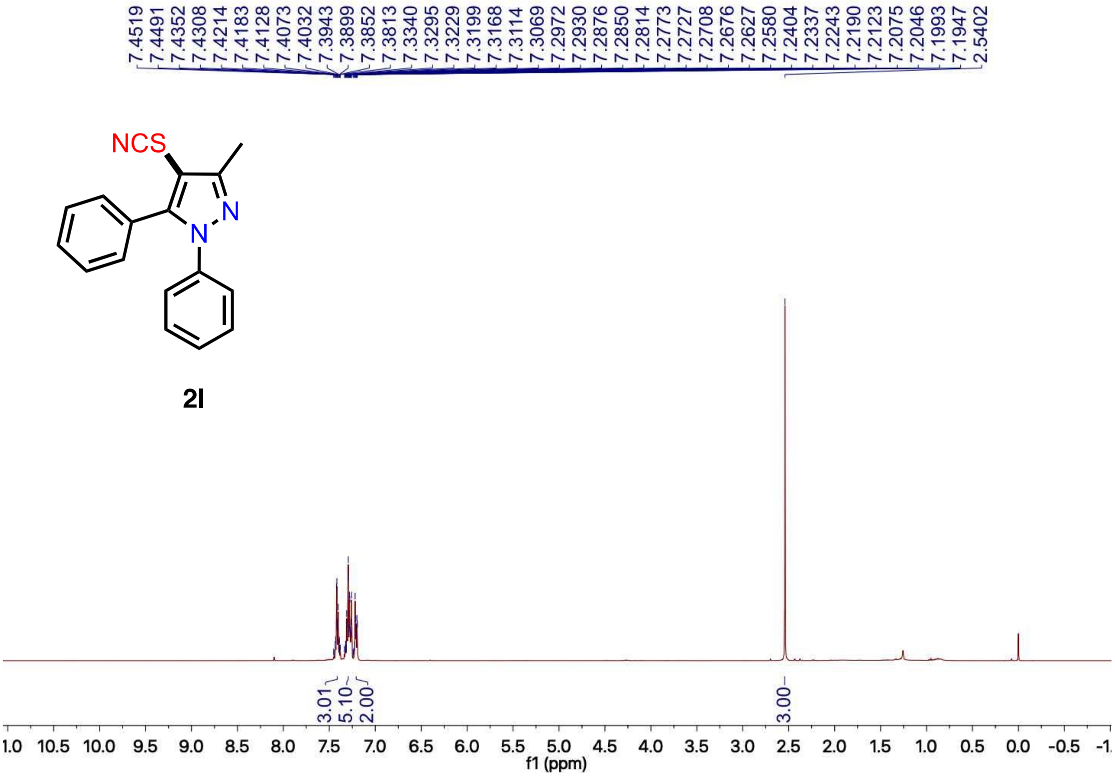
7.4519
7.4491
7.4352
7.4308
7.4214
7.4183
7.4128
7.4073
7.4032
7.3943
7.3899
7.3852
7.3813
7.3340
7.3295
7.3229
7.3199
7.3168
7.3114
7.3069
7.2972
7.2930
7.2876
7.2850
7.2814
7.2773
7.2727
7.2708
7.2676
7.2627
7.2580
7.2404
7.2337
7.2243
7.2190
7.2123
7.2075
7.2046
7.1993
7.1947
2.5402
NCS
N
N
2l
3.01
5.10
2.00
3.00
f1 (ppm)

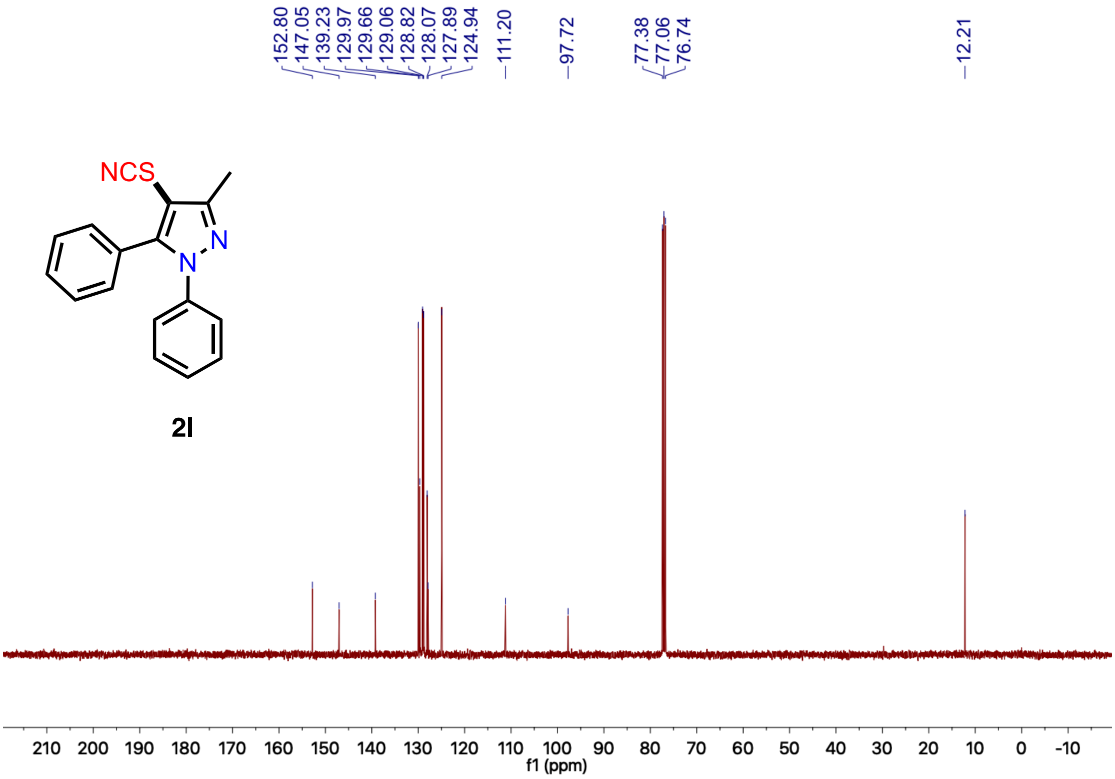
152.80
147.05
139.23
129.97
129.66
129.06
128.82
128.07
127.89
124.94
111.20
97.72
77.38
77.06
76.74
12.21
NCS
N
N
2l
f1 (ppm)

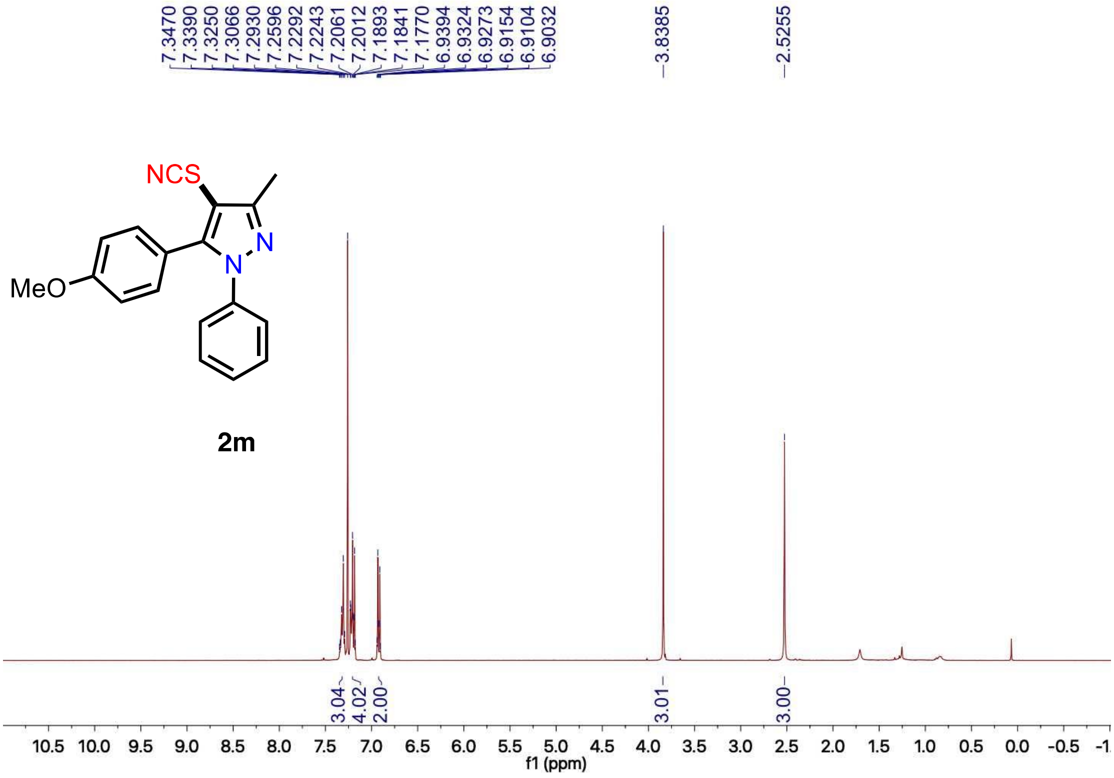
7.3470
7.3390
7.3250
7.3066
7.2930
7.2596
7.2292
7.2243
7.2061
7.2012
7.1893
7.1841
7.1770
6.9394
6.9324
6.9273
6.9154
6.9104
6.9032
3.8385
2.5255
NCS
N
N
MeO
3.04
4.02
2.00
3.01
3.00
f1 (ppm)

2m

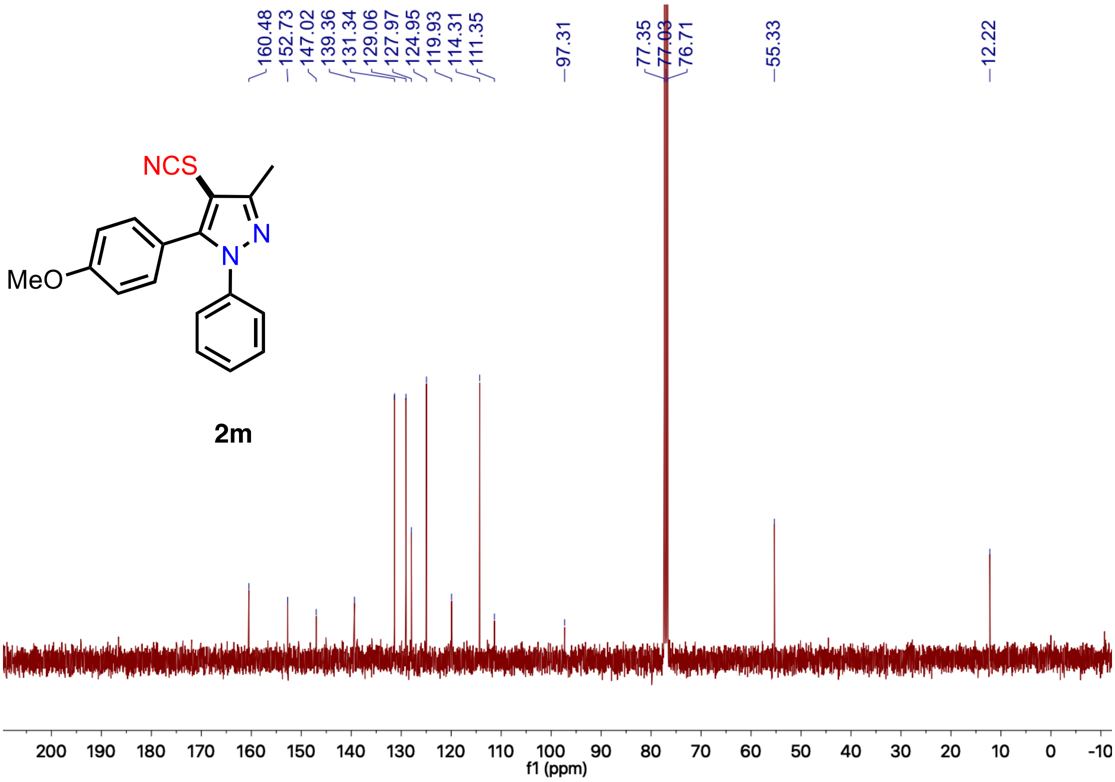
160.48
152.73
147.02
139.36
131.34
129.06
127.97
124.95
119.93
114.31
111.35
97.31
77.35
77.03
76.71
55.33
12.22
NCS
N
N
MeO
f1 (ppm)

2m

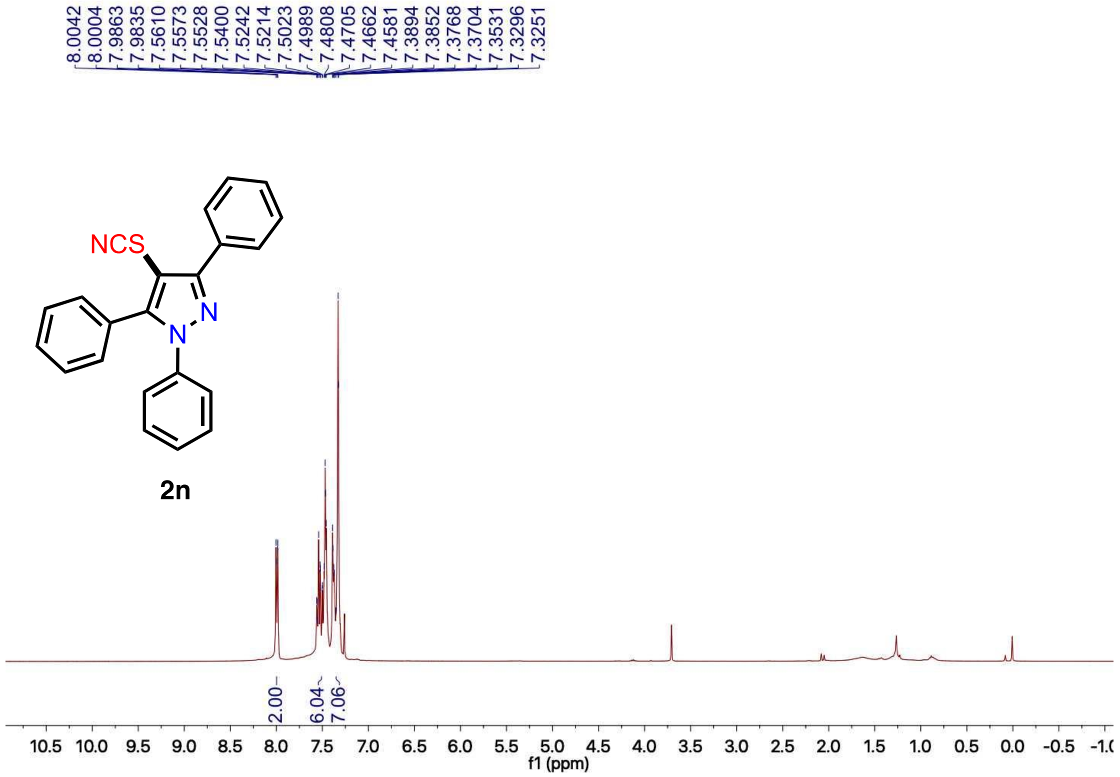
8.0042
8.0004
7.9863
7.9835
7.5610
7.5573
7.5528
7.5400
7.5242
7.5214
7.5023
7.4989
7.4808
7.4705
7.4662
7.4581
7.3894
7.3852
7.3768
7.3704
7.3531
7.3296
7.3251
NCS
N
N
2n
2.00
6.04
7.06
f1 (ppm)

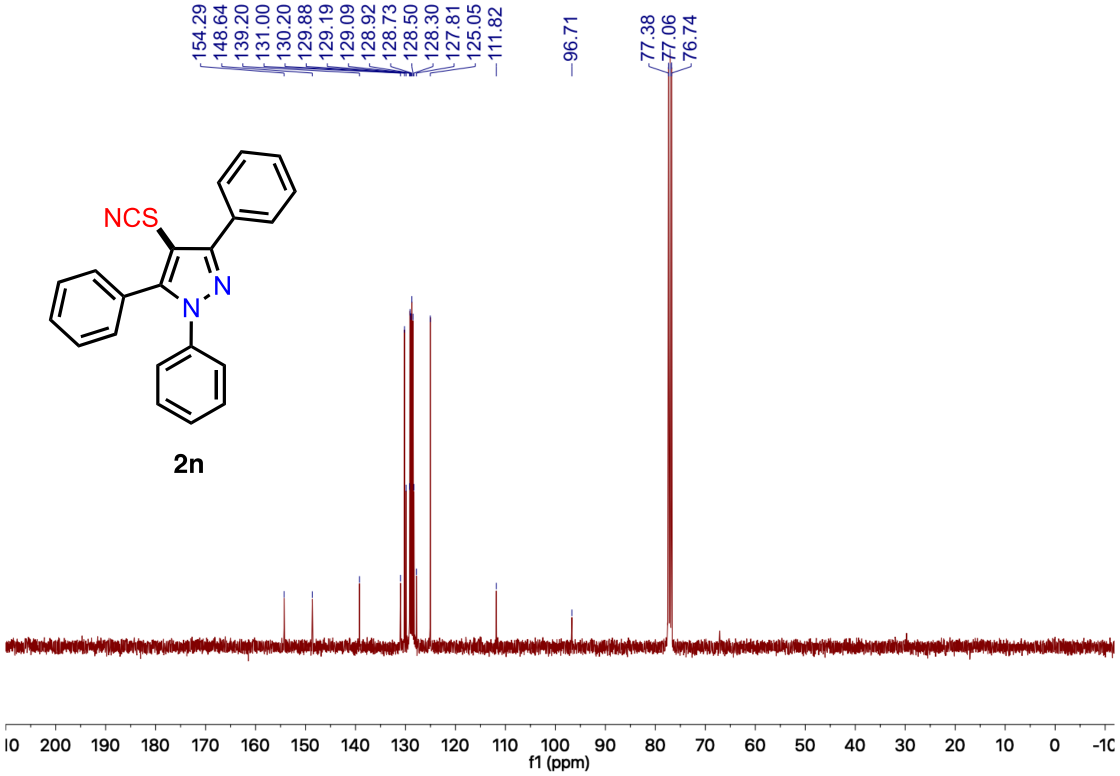
154.29
148.64
139.20
131.00
130.20
129.88
129.19
129.09
128.92
128.73
128.50
128.30
127.81
125.05
111.82
96.71
77.38
77.06
76.74
NCS
N
N
2n
f1 (ppm)

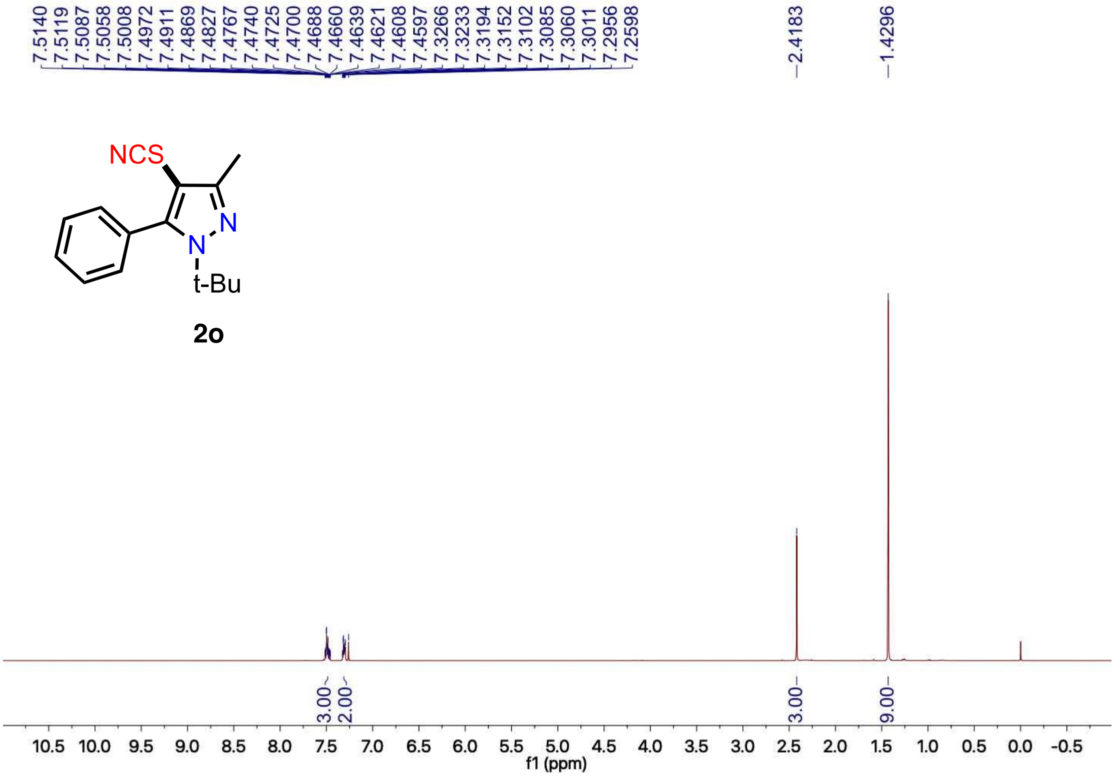

7.5140
7.5119
7.5087
7.5058
7.5008
7.4972
7.4911
7.4869
7.4827
7.4767
7.4740
7.4725
7.4700
7.4688
7.4660
7.4639
7.4621
7.4608
7.4597
7.3266
7.3233
7.3194
7.3152
7.3102
7.3085
7.3060
7.3011
7.2956
7.2598
2.4183
1.4296
NCS
N
N
t-Bu
2o
3.00
2.00
3.00
9.00
10.5 10.0 9.5 9.0 8.5 8.0 7.5 7.0 6.5 6.0 5.5 5.0 4.5 4.0 3.5 3.0 2.5 2.0 1.5 1.0 0.5 0.0 -0.5
f1 (ppm)

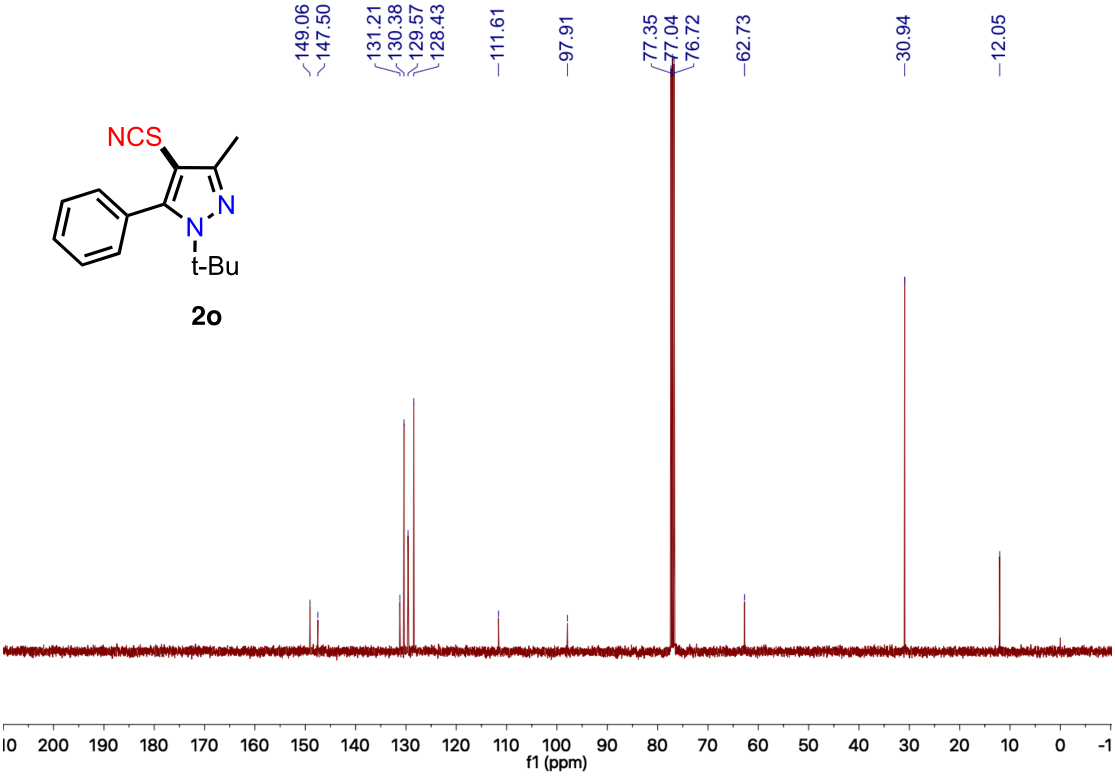

149.06
147.50
131.21
130.38
129.57
128.43
111.61
97.91
77.35
77.04
76.72
62.73
30.94
12.05
NCS
N
N
t-Bu
2o
200 190 180 170 160 150 140 130 120 110 100 90 80 70 60 50 40 30 20 10 0
f1 (ppm)

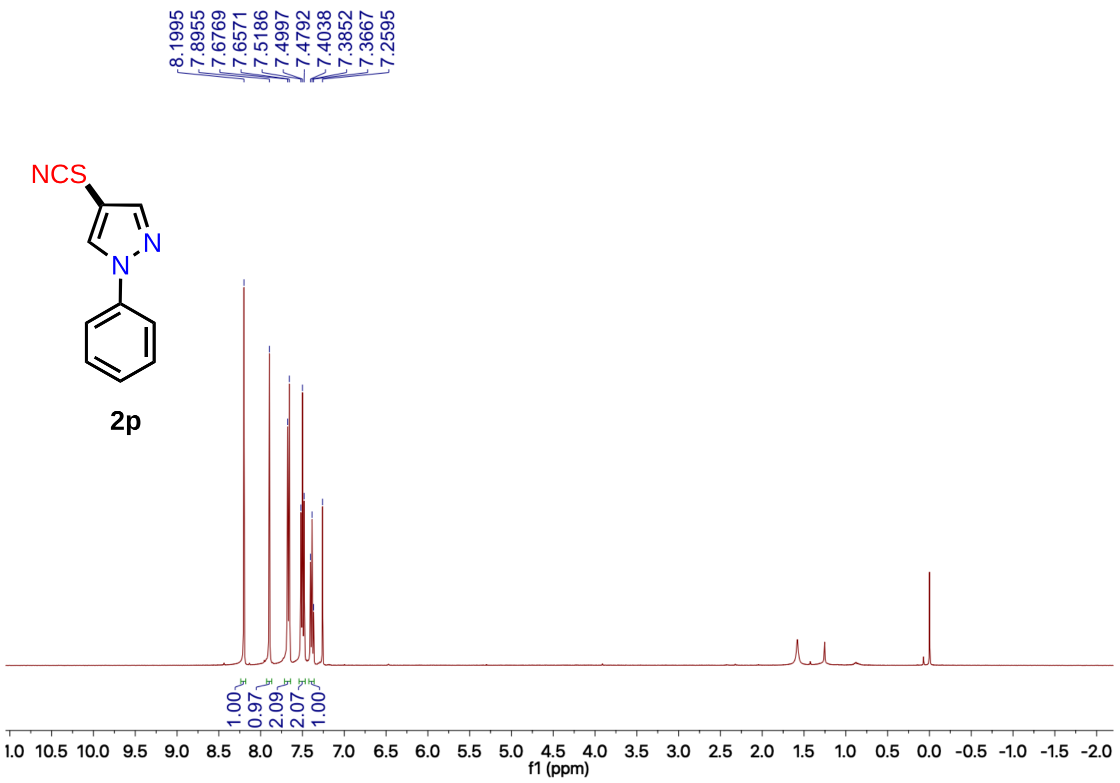
8.1995
7.8955
7.6769
7.6571
7.5186
7.4997
7.4792
7.4038
7.3852
7.3667
7.2595
NCS
N
N
2p
1.00
0.97
2.09
2.07
1.00
f1 (ppm)

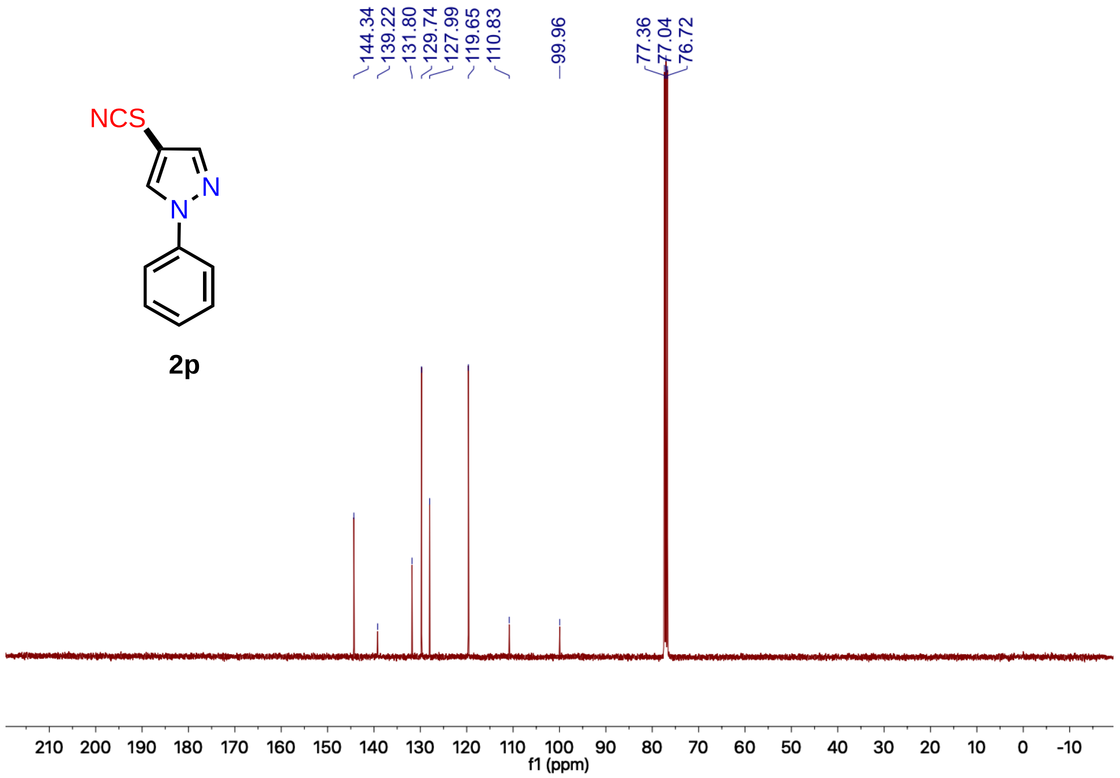
144.34
139.22
131.80
129.74
127.99
119.65
110.83
99.96
77.36
77.04
76.72
NCS
N
N
2p
f1 (ppm)

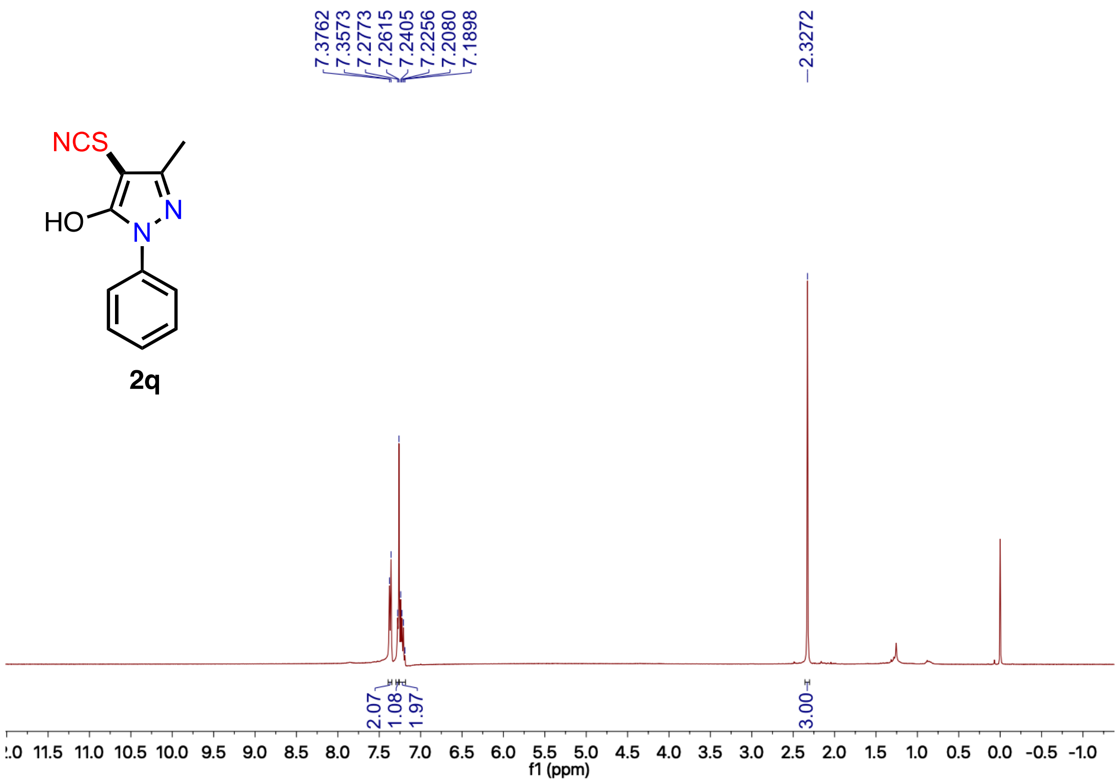
7.3762
7.3573
7.2773
7.2615
7.2405
7.2256
7.2080
7.1898
2.3272
NCS
HO
N
N
2q
2.07
1.08
1.97
3.00
f1 (ppm)

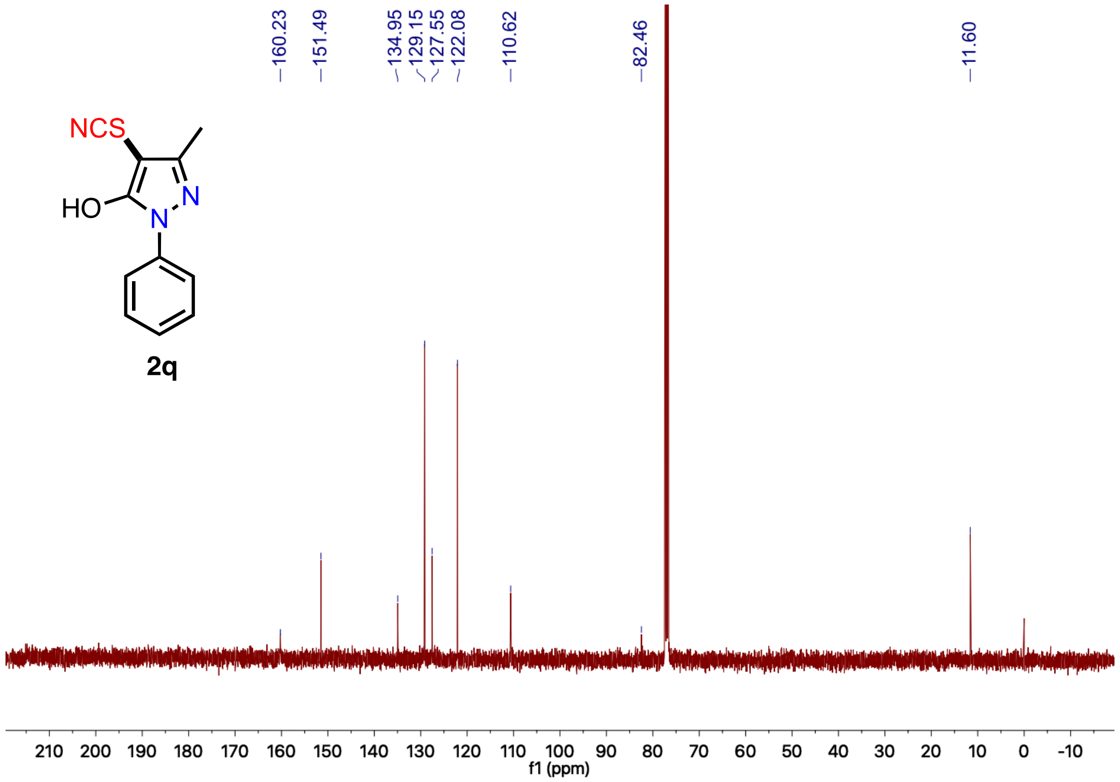
160.23
151.49
134.95
129.15
127.55
122.08
110.62
82.46
11.60
NCS
HO
N
N
2q
f1 (ppm)

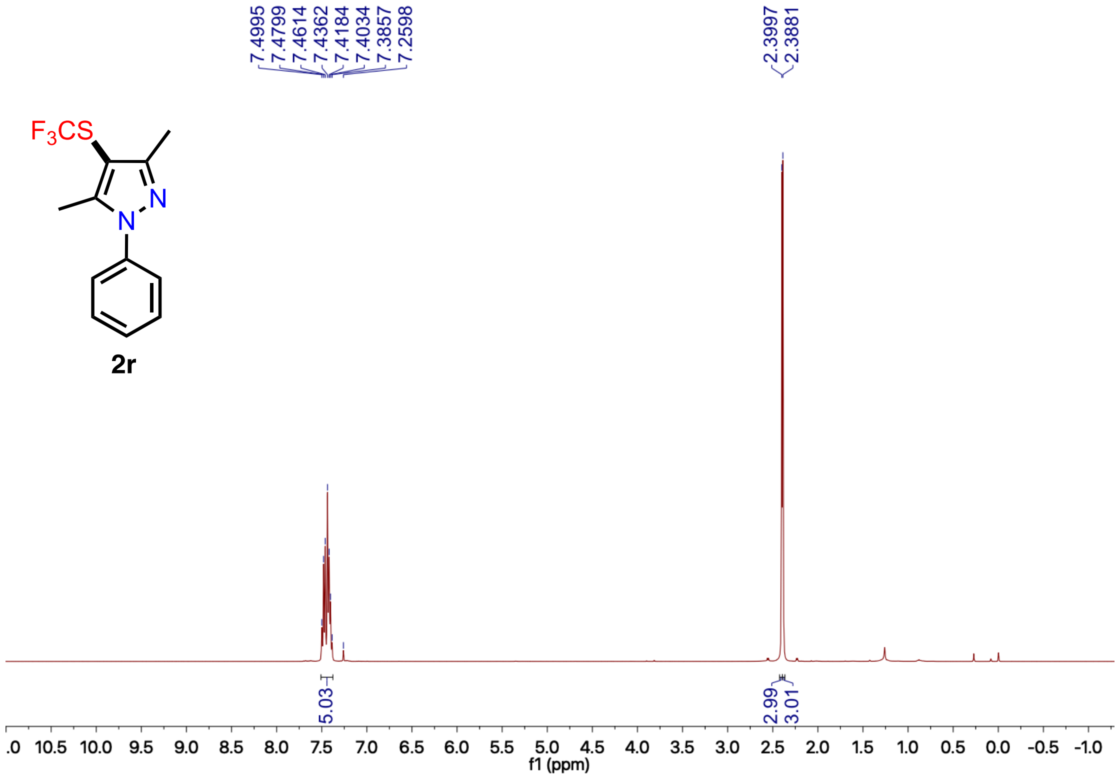
7.4995
7.4799
7.4614
7.4362
7.4184
7.4034
7.3857
7.2598
2.3997
2.3881
F3CS
N
N
2r
5.03
2.99
3.01
.0 10.5 10.0 9.5 9.0 8.5 8.0 7.5 7.0 6.5 6.0 5.5 5.0 4.5 4.0 3.5 3.0 2.5 2.0 1.5 1.0 0.5 0.0 -0.5 -1.0
f1 (ppm)

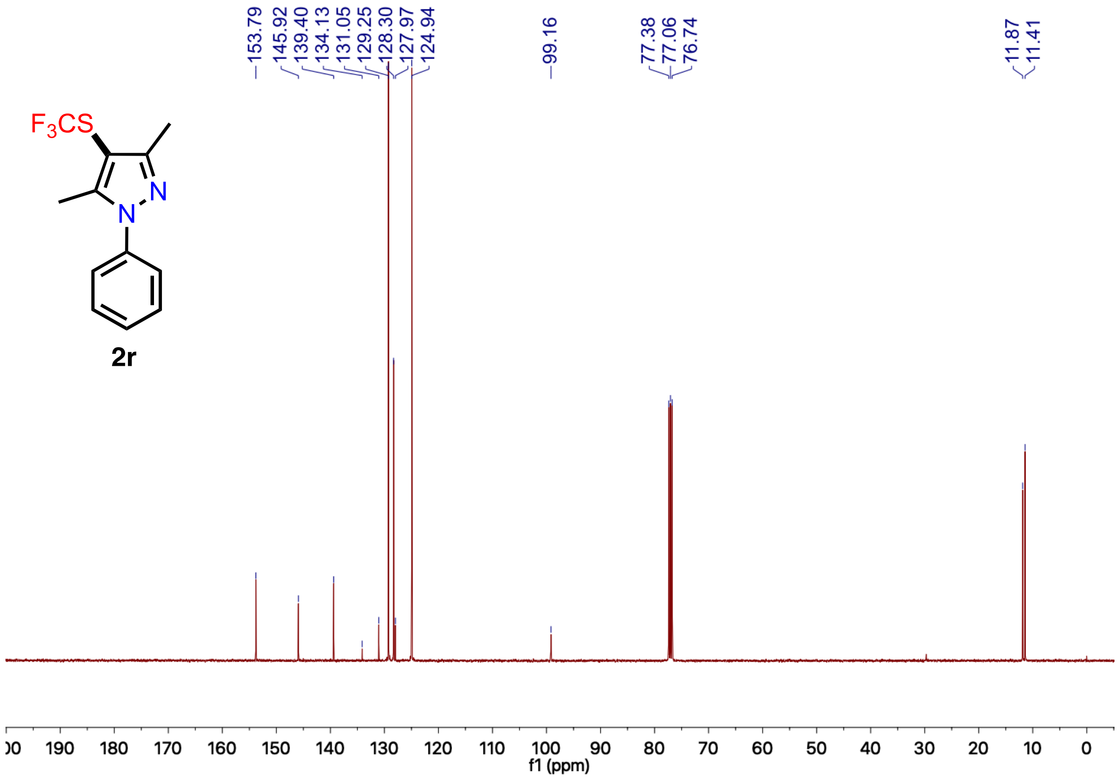
153.79
145.92
139.40
134.13
131.05
129.25
128.30
127.97
124.94
99.16
77.38
77.06
76.74
11.87
11.41
F3CS
N
N
2r
00 190 180 170 160 150 140 130 120 110 100 90 80 70 60 50 40 30 20 10 0
f1 (ppm)

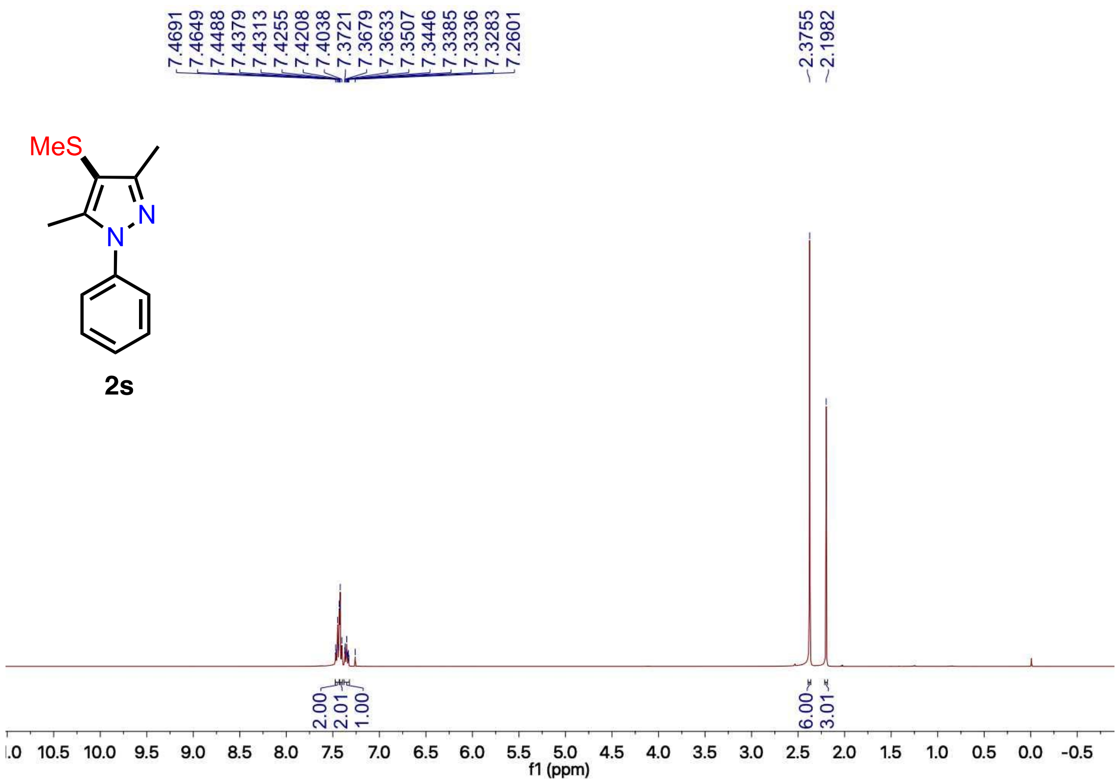
7.4691
7.4649
7.4488
7.4379
7.4313
7.4255
7.4208
7.4038
7.3721
7.3679
7.3633
7.3507
7.3446
7.3385
7.3336
7.3283
7.2601
2.3755
2.1982
MeS
N
N
2s
2.00
2.01
1.00
6.00
3.01
f1 (ppm)

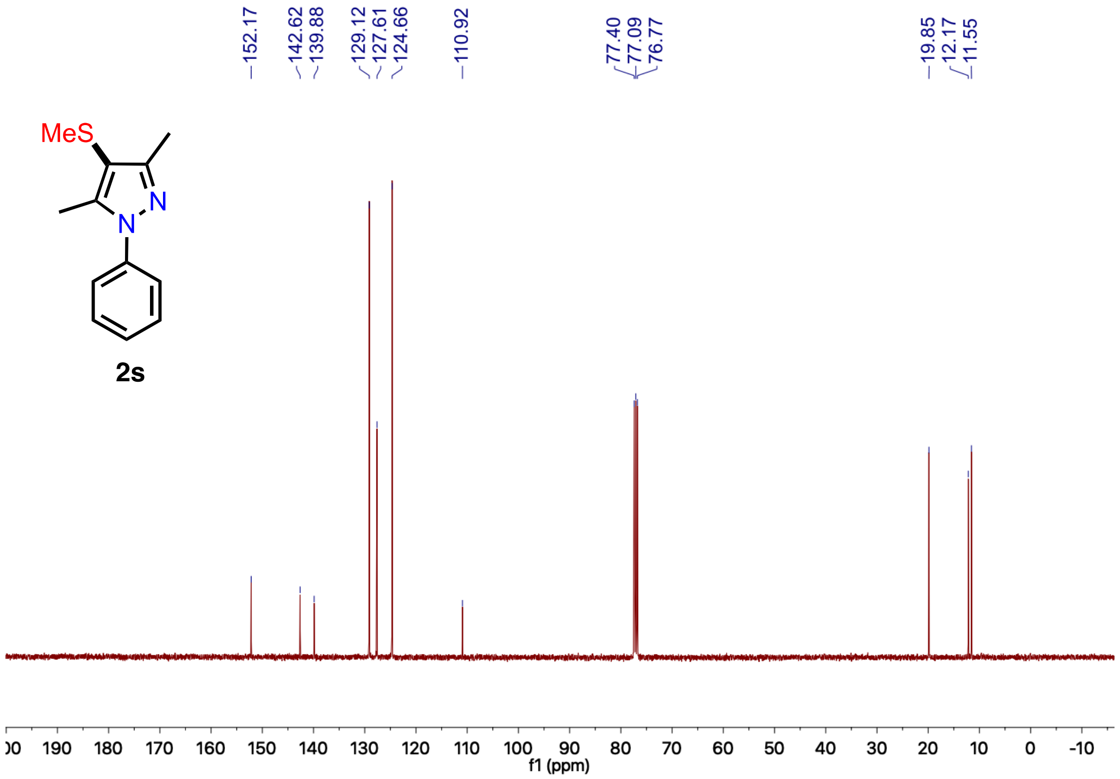
152.17
142.62
139.88
129.12
127.61
124.66
110.92
77.40
77.09
76.77
19.85
12.17
11.55
MeS
N
N
2s
f1 (ppm)

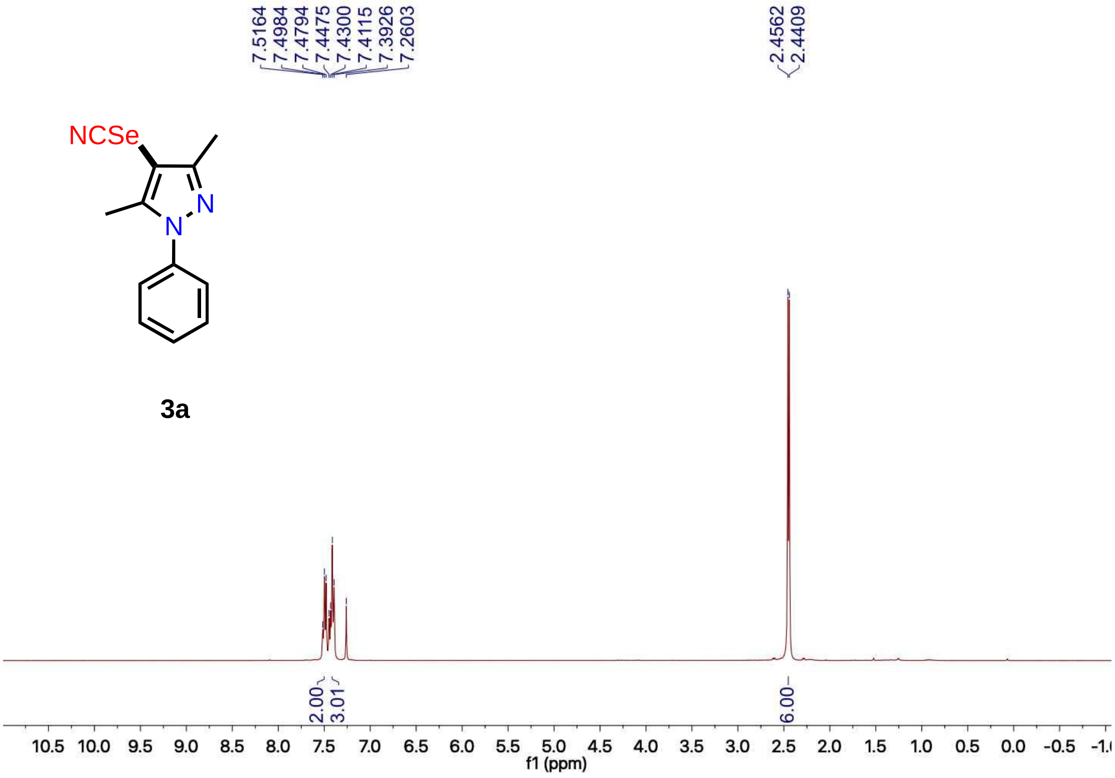
7.5164
7.4984
7.4794
7.4475
7.4300
7.4115
7.3926
7.2603
2.4562
2.4409
NCSe
N
N
3a
2.00
3.01
6.00
10.5 10.0 9.5 9.0 8.5 8.0 7.5 7.0 6.5 6.0 5.5 5.0 4.5 4.0 3.5 3.0 2.5 2.0 1.5 1.0 0.5 0.0 -0.5
f1 (ppm)

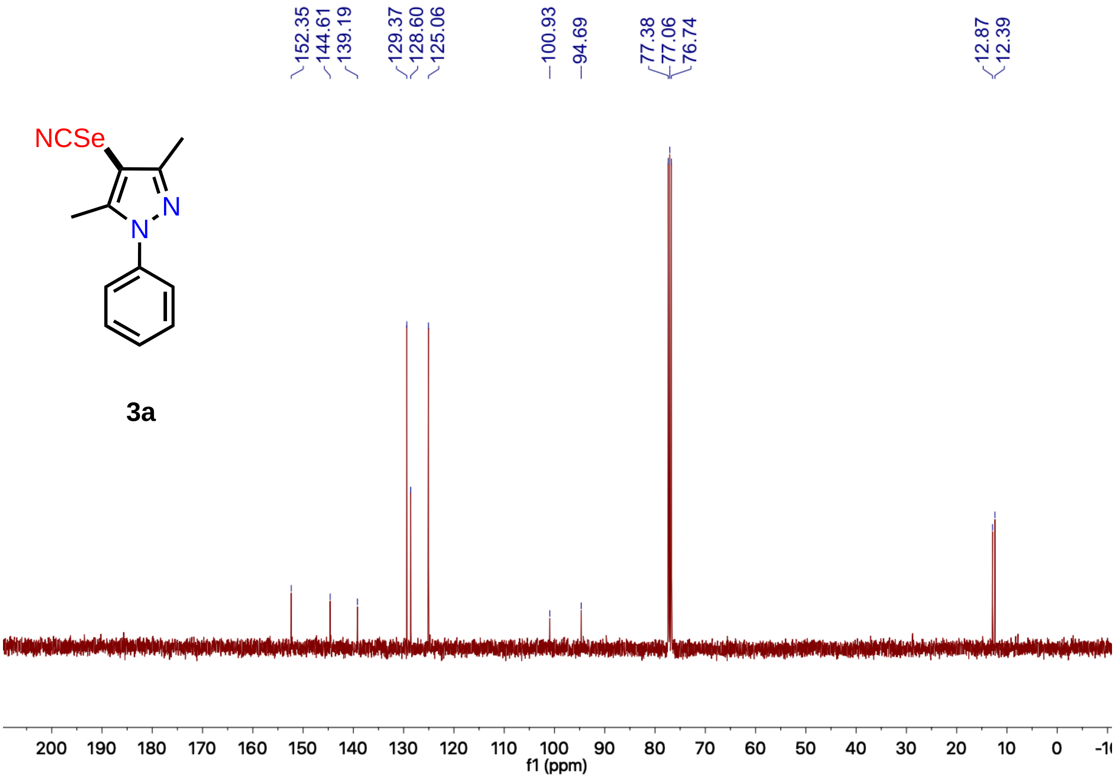
152.35
144.61
139.19
129.37
128.60
125.06
100.93
94.69
77.38
77.06
76.74
12.87
12.39
NCSe
N
N
3a
200 190 180 170 160 150 140 130 120 110 100 90 80 70 60 50 40 30 20 10 0
f1 (ppm)

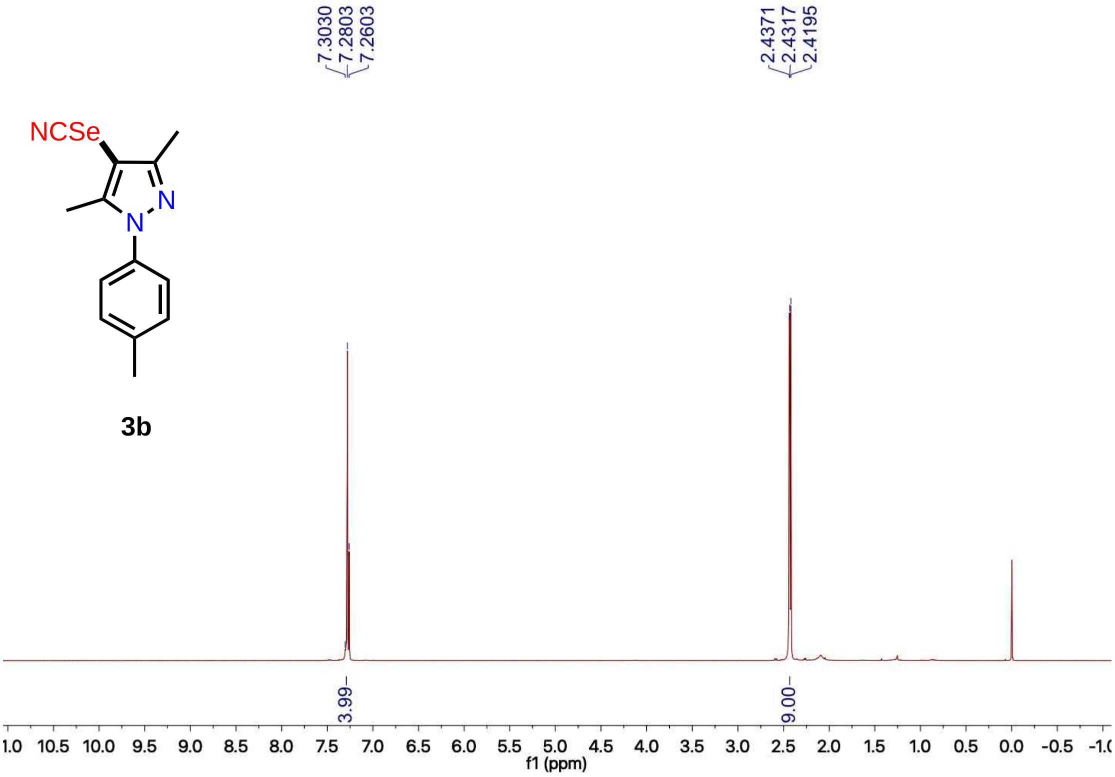
NCSe
N
N
7.3030
7.2803
7.2603
2.4371
2.4317
2.4195
3.99
9.00
f1 (ppm)

3b

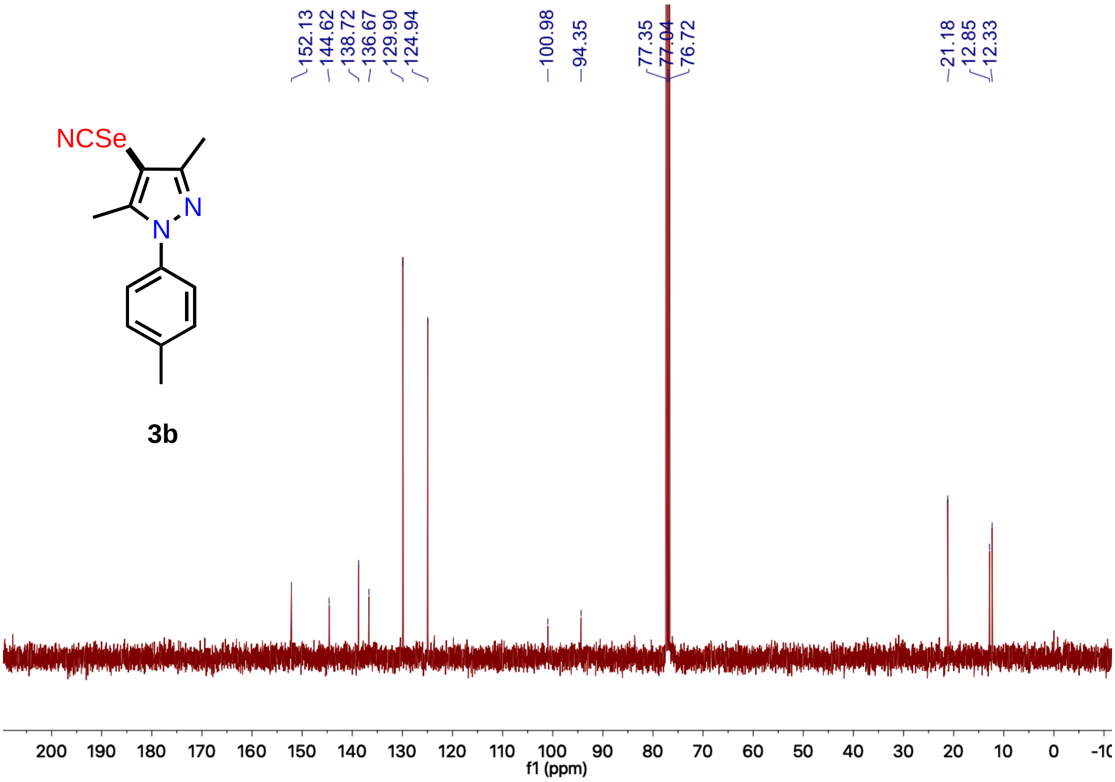
NCSe
N
N
152.13
144.62
138.72
136.67
129.90
124.94
100.98
94.35
77.35
77.04
76.72
21.18
12.85
12.33
f1 (ppm)

3b

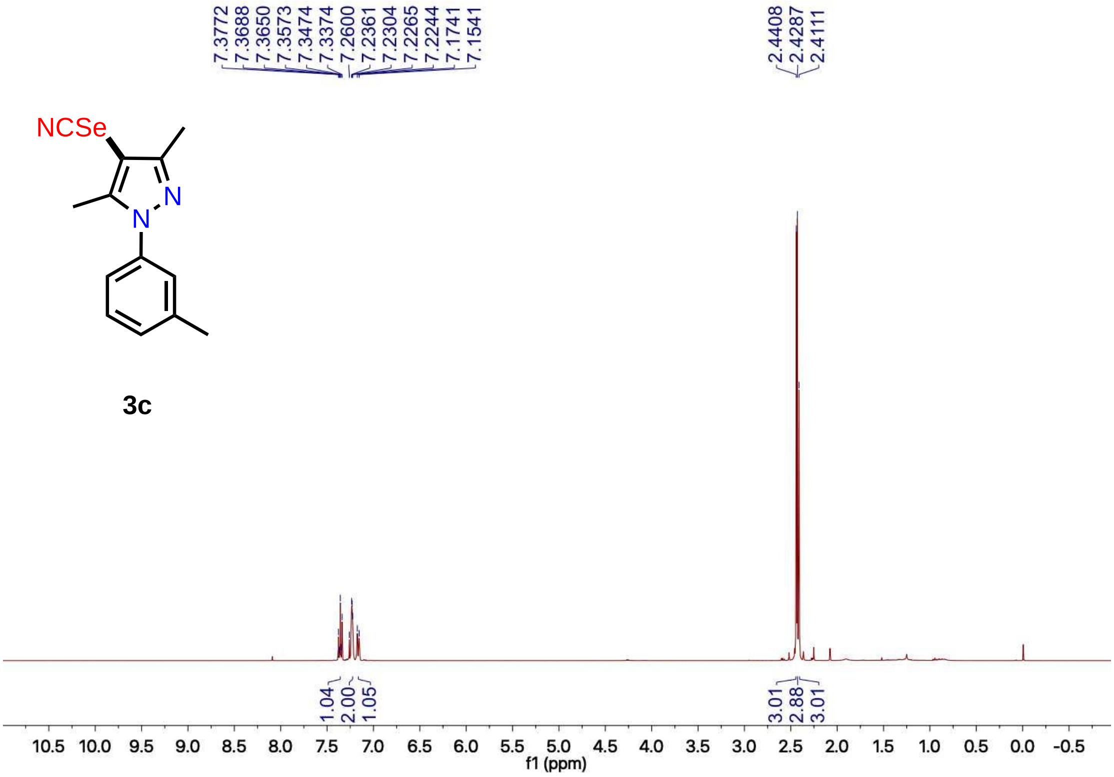
7.3772
7.3688
7.3650
7.3573
7.3474
7.3374
7.2600
7.2361
7.2304
7.2265
7.2244
7.1741
7.1541
2.4408
2.4287
2.4111
NCSe
N
N
3c
1.04
2.00
1.05
3.01
2.88
3.01
10.5 10.0 9.5 9.0 8.5 8.0 7.5 7.0 6.5 6.0 5.5 5.0 4.5 4.0 3.5 3.0 2.5 2.0 1.5 1.0 0.5 0.0 -0.5
f1 (ppm)

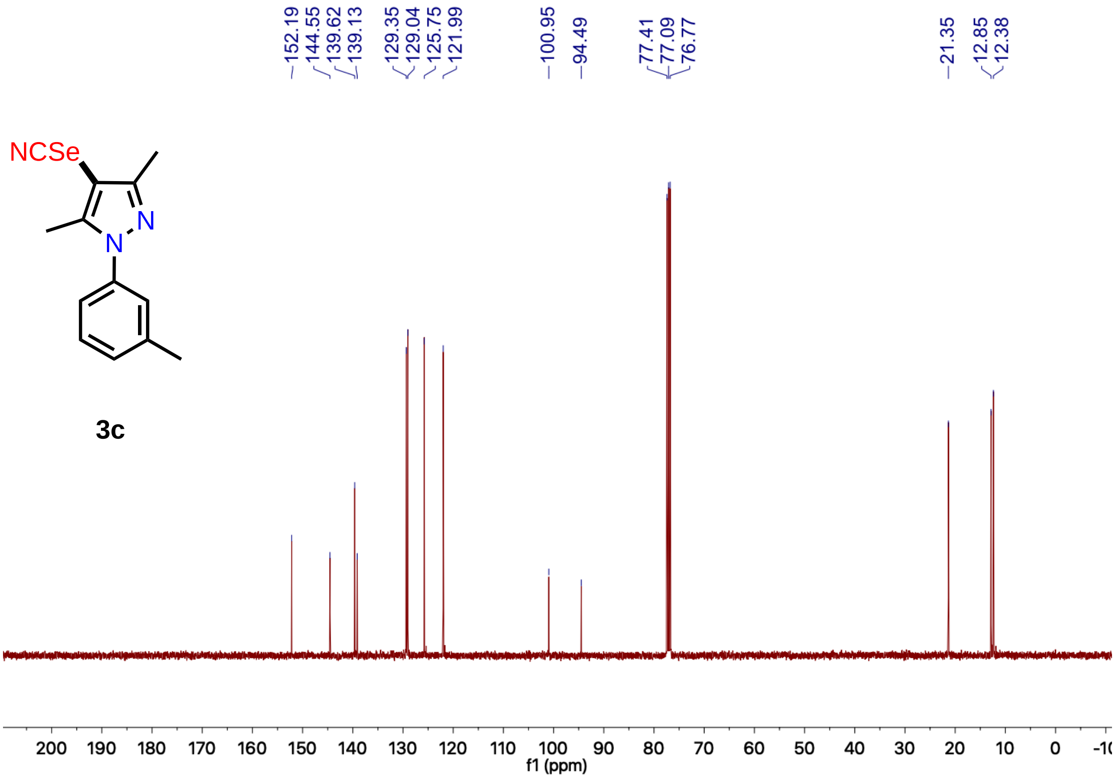
152.19
144.55
139.62
139.13
129.35
129.04
125.75
121.99
100.95
94.49
77.41
77.09
76.77
21.35
12.85
12.38
NCSe
N
N
3c
200 190 180 170 160 150 140 130 120 110 100 90 80 70 60 50 40 30 20 10 0
f1 (ppm)

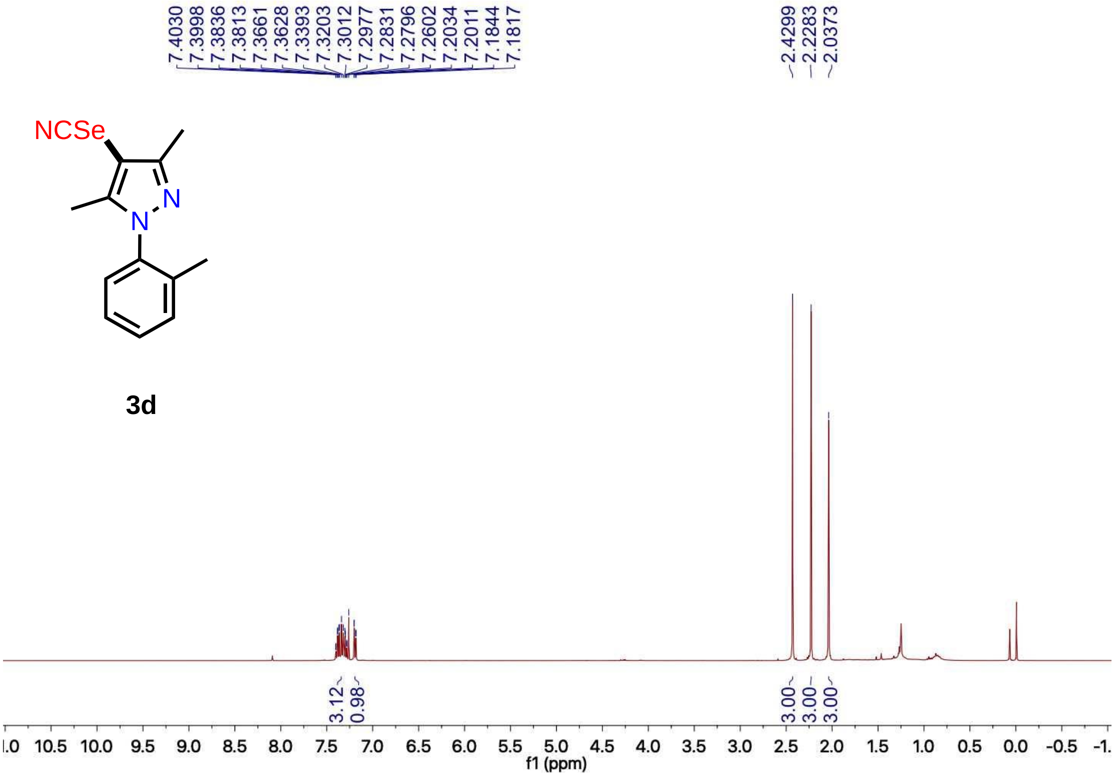
7.4030
7.3998
7.3836
7.3813
7.3661
7.3628
7.3393
7.3203
7.3012
7.2977
7.2831
7.2796
7.2602
7.2034
7.2011
7.1844
7.1817
2.4299
2.2283
2.0373
NCSe
N
N
3d
3.12
0.98
3.00
3.00
3.00
f1 (ppm)

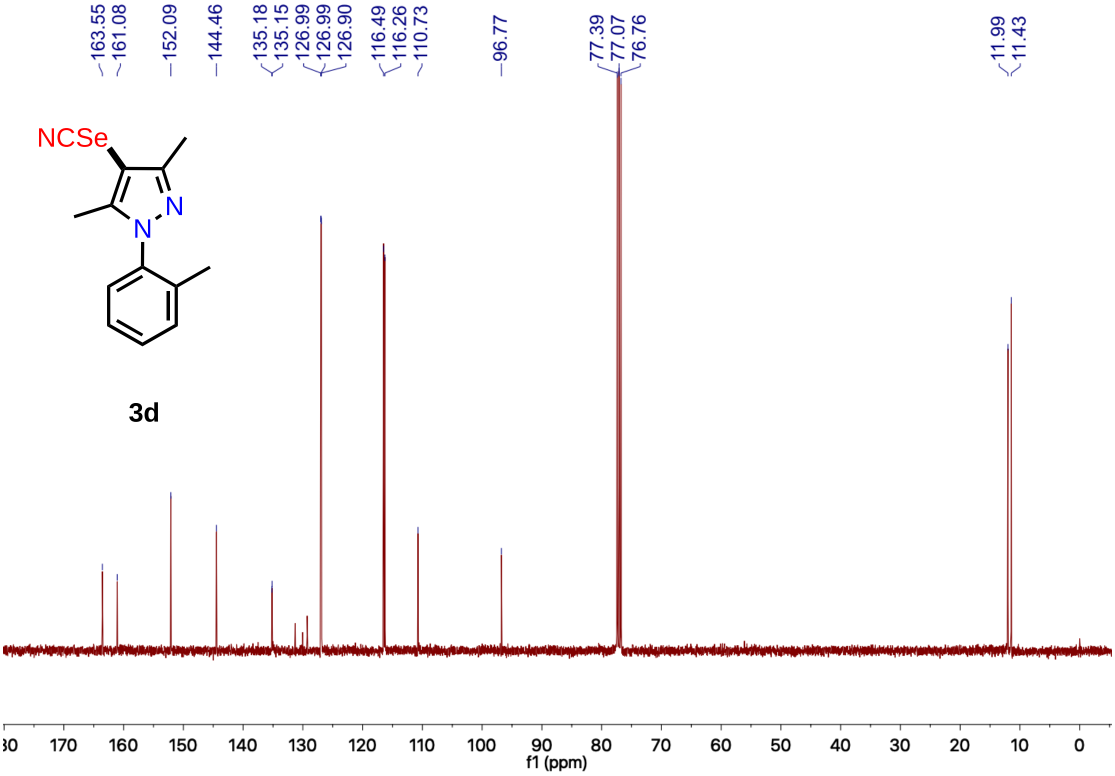
163.55
161.08
152.09
144.46
135.18
135.15
126.99
126.99
126.90
116.49
116.26
110.73
96.77
77.39
77.07
76.76
11.99
11.43
NCSe
N
N
3d
f1 (ppm)

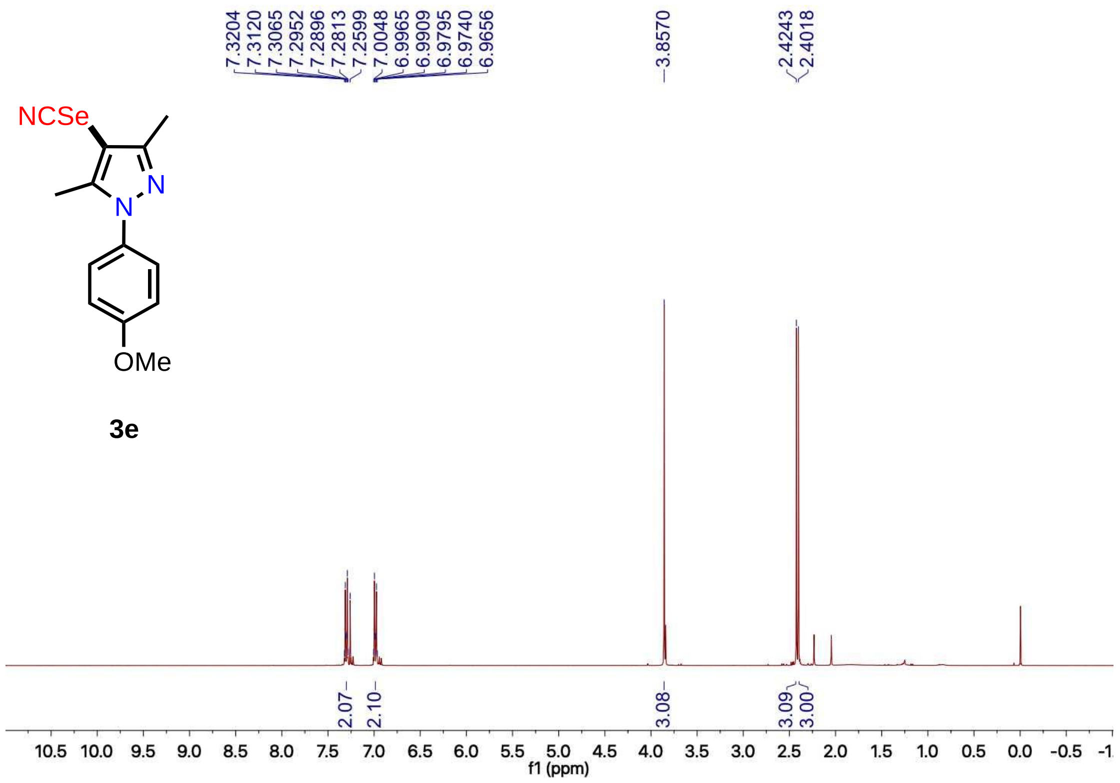

NCSe
N
N
OMe
7.3204
7.3120
7.3065
7.2952
7.2896
7.2813
7.2599
7.0048
6.9965
6.9909
6.9795
6.9740
6.9656
3.8570
2.4243
2.4018
2.07
2.10
3.08
3.09
3.00
10.5 10.0 9.5 9.0 8.5 8.0 7.5 7.0 6.5 6.0 5.5 5.0 4.5 4.0 3.5 3.0 2.5 2.0 1.5 1.0 0.5 0.0 -0.5 -1
f1 (ppm)

3e

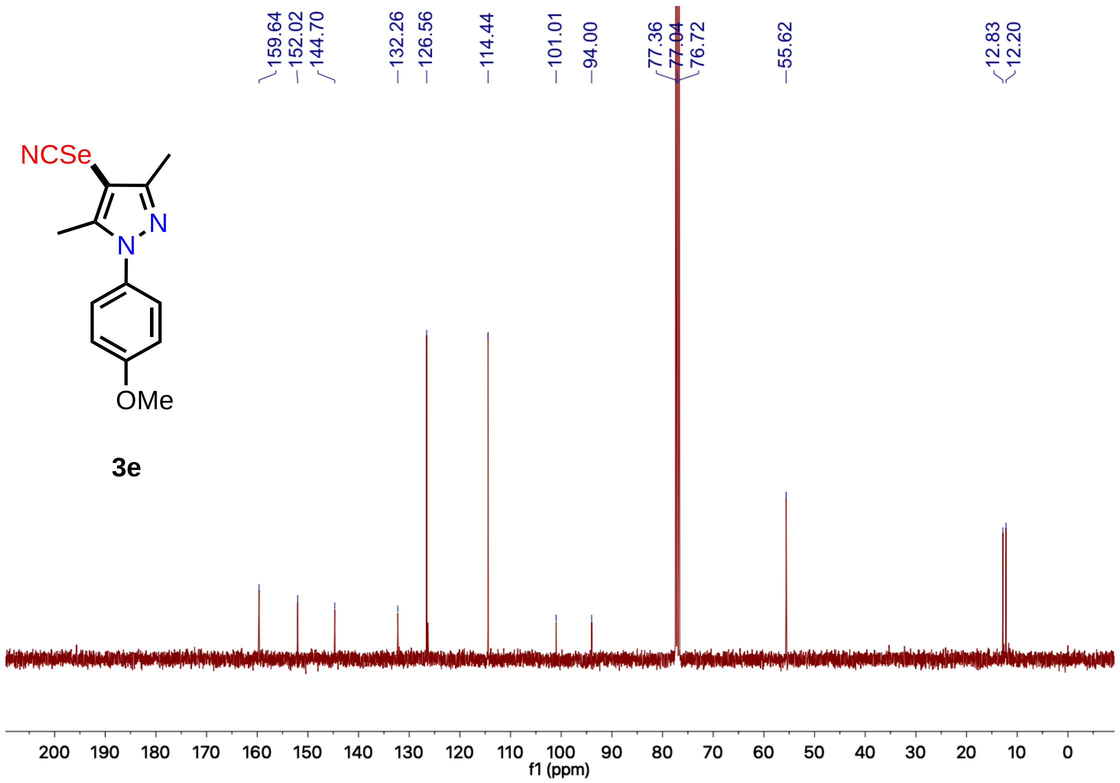

NCSe
N
N
OMe
159.64
152.02
144.70
132.26
126.56
114.44
101.01
94.00
77.36
77.04
76.72
55.62
12.83
12.20
200 190 180 170 160 150 140 130 120 110 100 90 80 70 60 50 40 30 20 10 0
f1 (ppm)

3e

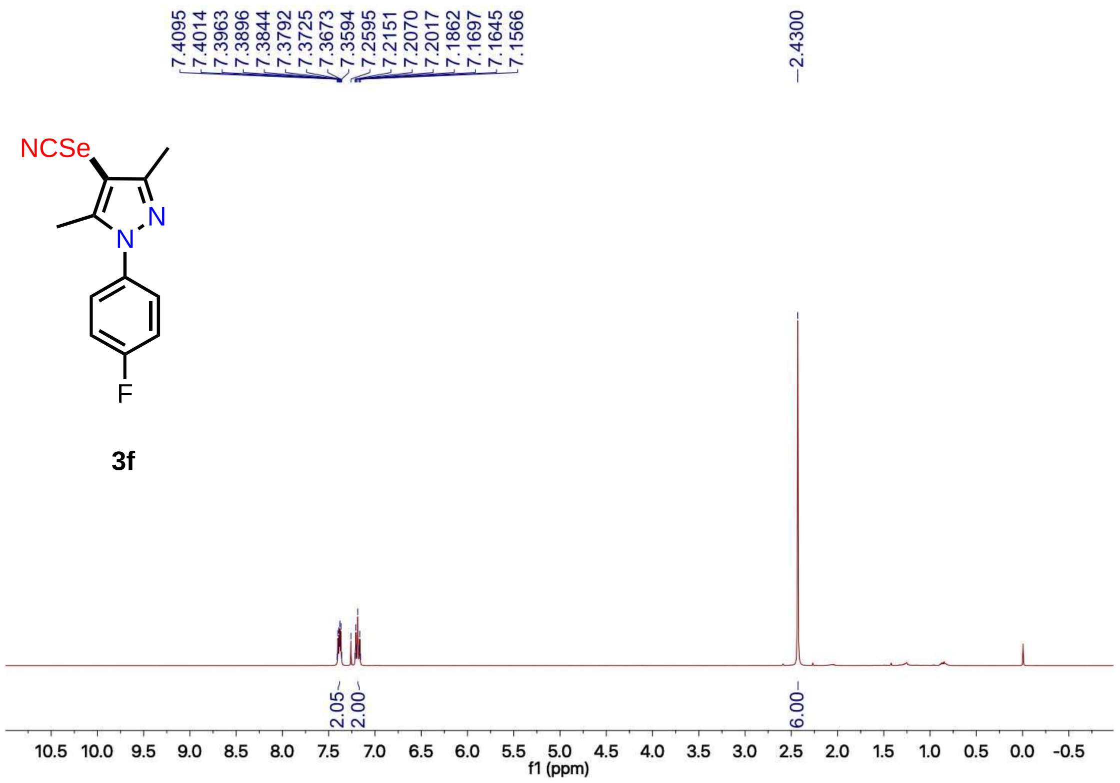
7.4095
7.4014
7.3963
7.3896
7.3844
7.3792
7.3725
7.3673
7.3594
7.2595
7.2151
7.2070
7.2017
7.1862
7.1697
7.1645
7.1566
2.4300
NCSe
N
N
F
3f
2.05
2.00
6.00
10.5 10.0 9.5 9.0 8.5 8.0 7.5 7.0 6.5 6.0 5.5 5.0 4.5 4.0 3.5 3.0 2.5 2.0 1.5 1.0 0.5 0.0 -0.5
f1 (ppm)

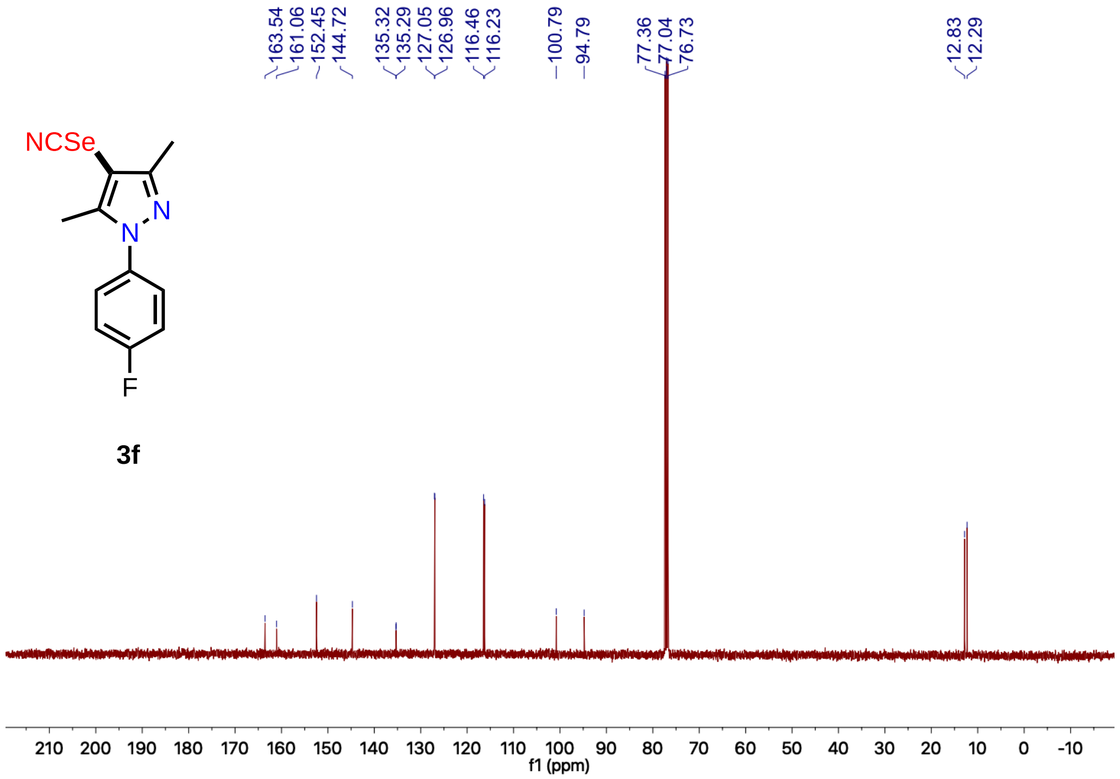
163.54
161.06
152.45
144.72
135.32
135.29
127.05
126.96
116.46
116.23
100.79
94.79
77.36
77.04
76.73
12.83
12.29
NCSe
N
N
F
3f
210 200 190 180 170 160 150 140 130 120 110 100 90 80 70 60 50 40 30 20 10 0 -10
f1 (ppm)

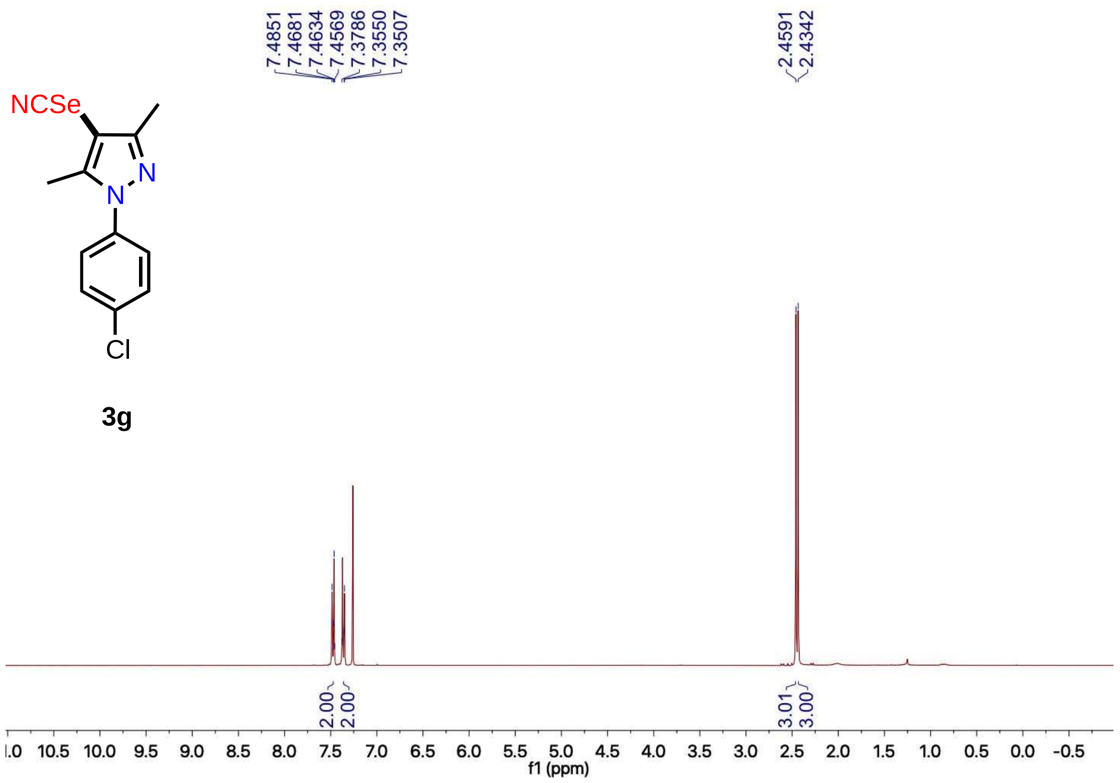
7.4851
7.4681
7.4634
7.4569
7.3786
7.3550
7.3507
2.4591
2.4342
NCSe
N
N
Cl
3g
2.00
2.00
3.01
3.00
f1 (ppm)

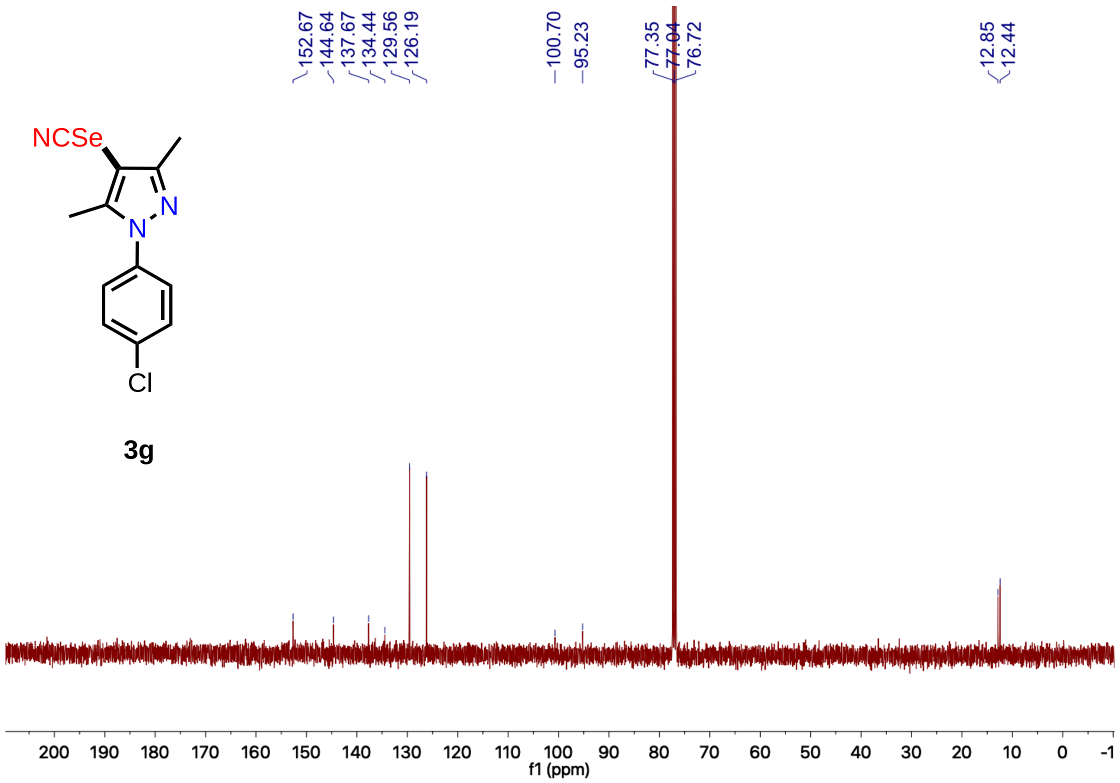
152.67
144.64
137.67
134.44
129.56
126.19
100.70
95.23
77.35
77.04
76.72
12.85
12.44
NCSe
N
N
Cl
3g
f1 (ppm)

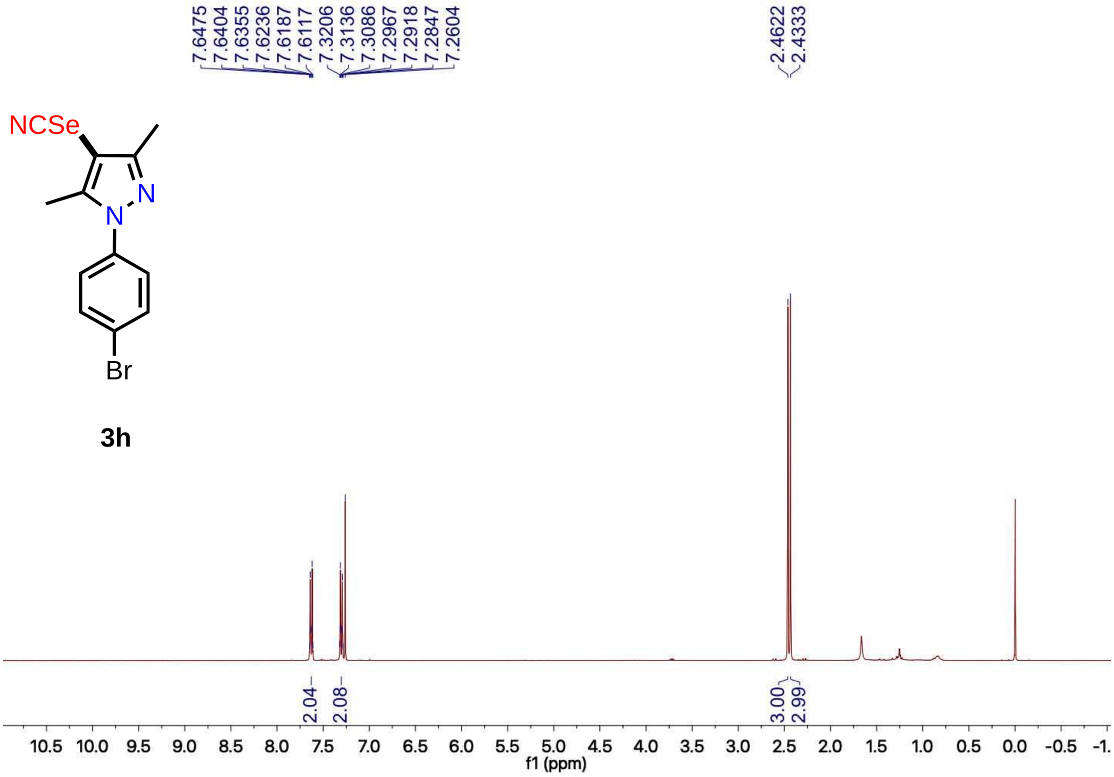
7.6475
7.6404
7.6355
7.6236
7.6187
7.6117
7.3206
7.3136
7.3086
7.2967
7.2918
7.2847
7.2604
2.4622
2.4333
NCSe
N
N
Br
3h
2.04
2.08
3.00
2.99
10.5 10.0 9.5 9.0 8.5 8.0 7.5 7.0 6.5 6.0 5.5 5.0 4.5 4.0 3.5 3.0 2.5 2.0 1.5 1.0 0.5 0.0 -0.5 -1.
f1 (ppm)

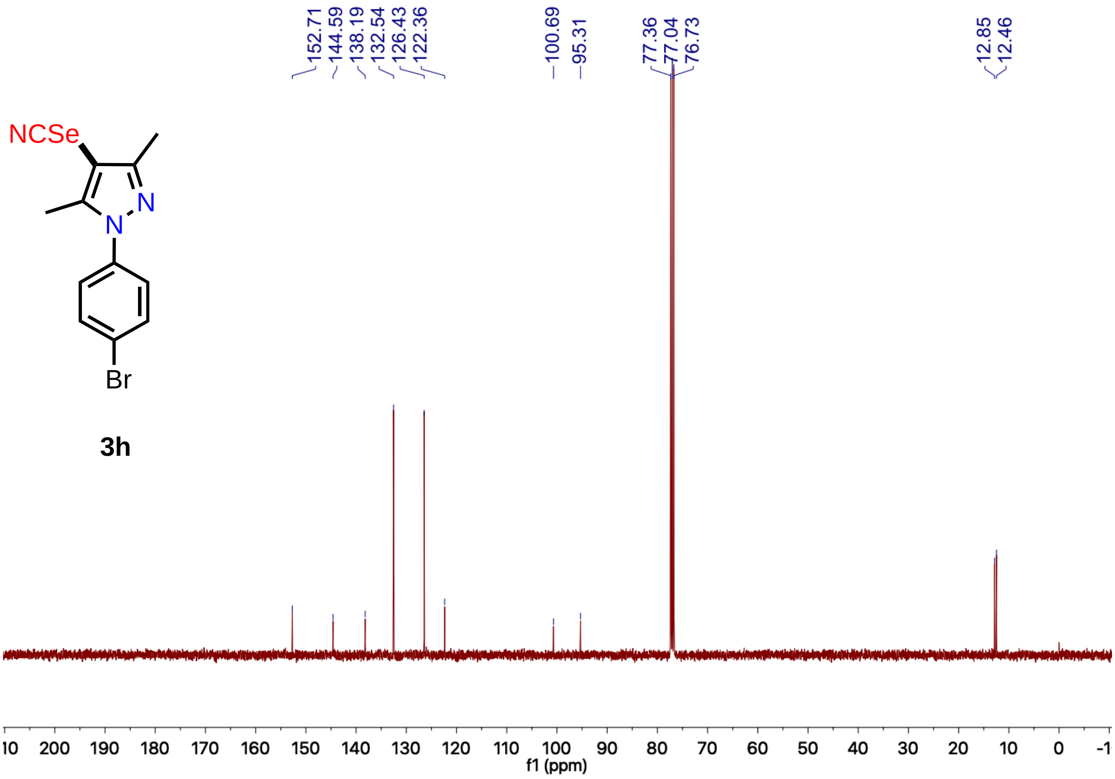
152.71
144.59
138.19
132.54
126.43
122.36
100.69
95.31
77.36
77.04
76.73
12.85
12.46
NCSe
N
N
Br
3h
10 200 190 180 170 160 150 140 130 120 110 100 90 80 70 60 50 40 30 20 10 0 -1
f1 (ppm)

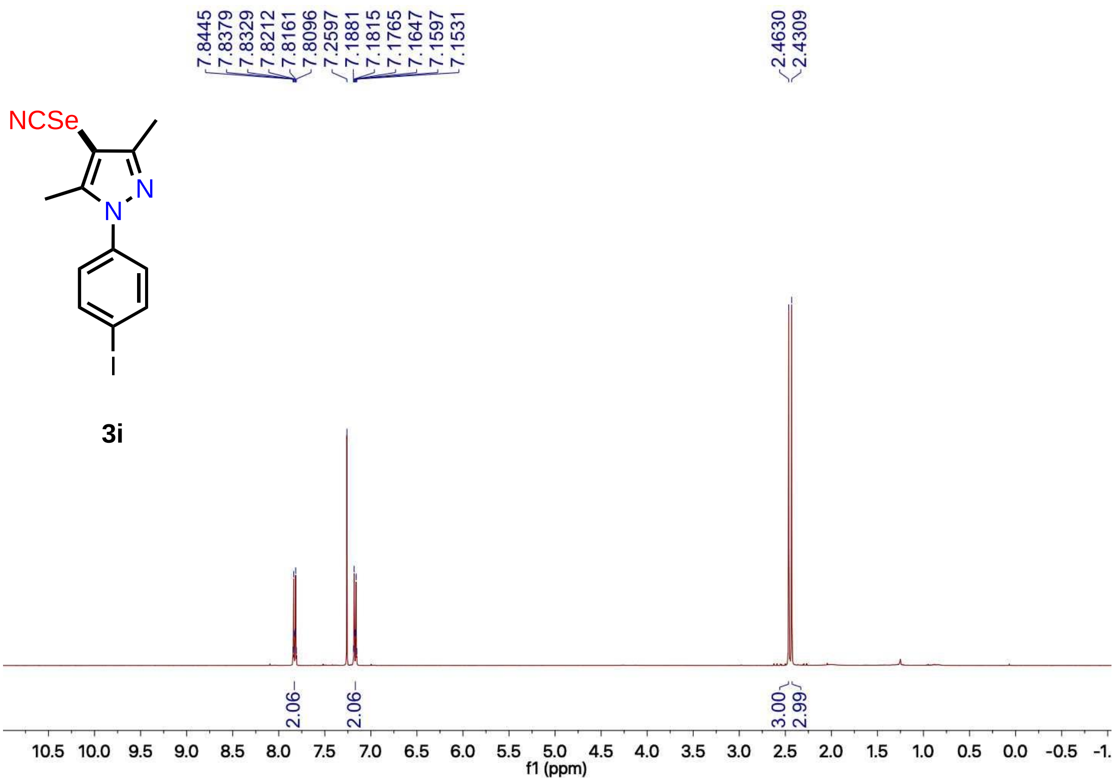
7.8445
7.8379
7.8329
7.8212
7.8161
7.8096
7.2597
7.1881
7.1815
7.1765
7.1647
7.1597
7.1531
2.4630
2.4309
NCSe
N
N
I
2.06
2.06
3.00
2.99
10.5 10.0 9.5 9.0 8.5 8.0 7.5 7.0 6.5 6.0 5.5 5.0 4.5 4.0 3.5 3.0 2.5 2.0 1.5 1.0 0.5 0.0 -0.5 -1.
f1 (ppm)

3i

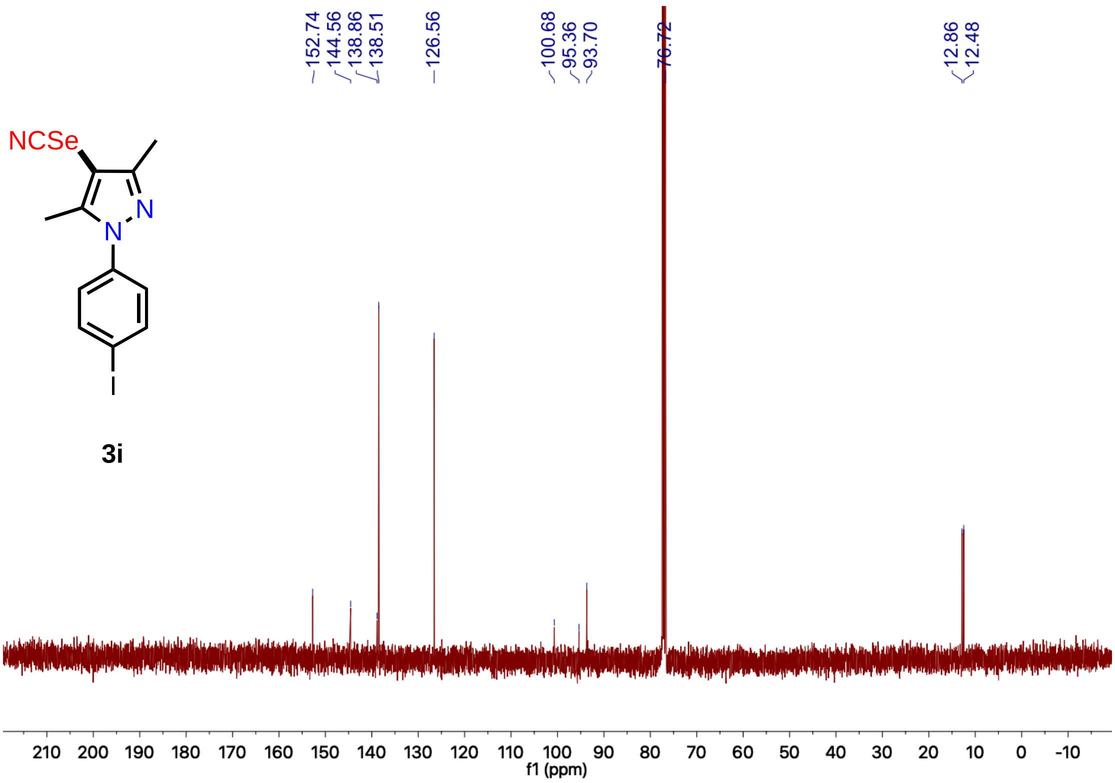
152.74
144.56
138.86
138.51
126.56
100.68
95.36
93.70
76.72
12.86
12.48
NCSe
N
N
I
210 200 190 180 170 160 150 140 130 120 110 100 90 80 70 60 50 40 30 20 10 0 -10
f1 (ppm)

3i

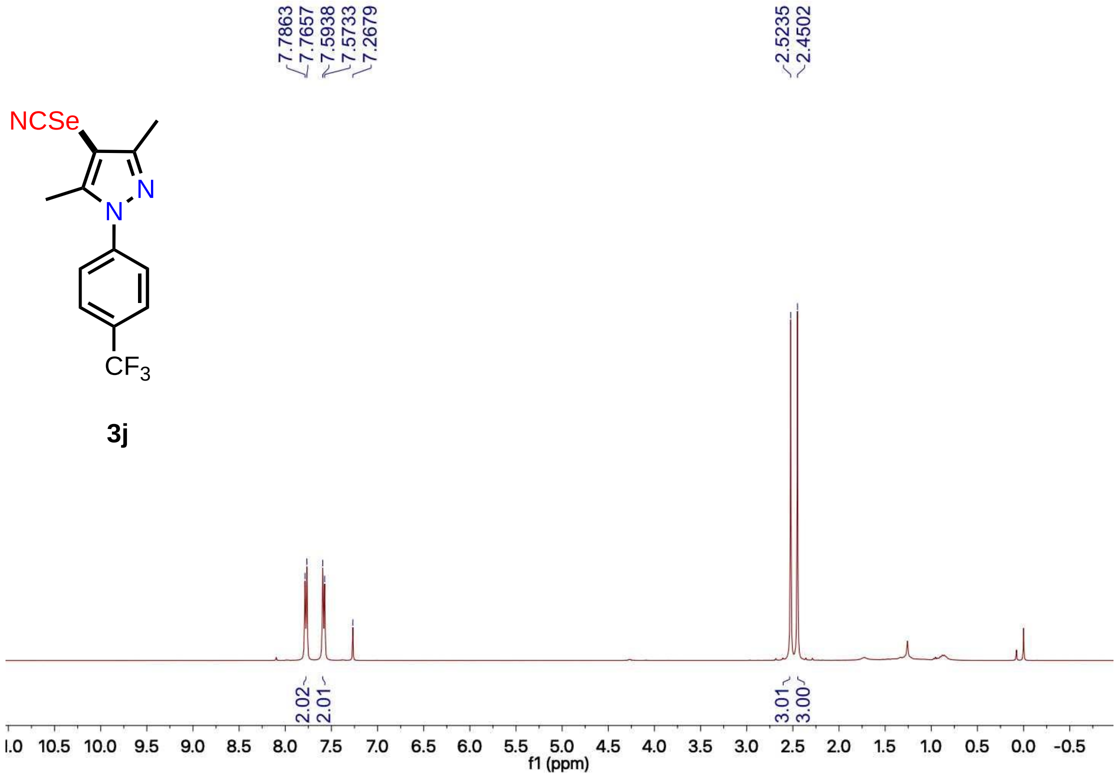
7.7863
7.7657
7.5938
7.5733
7.2679
2.5235
2.4502
NCSe
N
N
CF3
3j
2.02
2.01
3.01
3.00
11.0 10.5 10.0 9.5 9.0 8.5 8.0 7.5 7.0 6.5 6.0 5.5 5.0 4.5 4.0 3.5 3.0 2.5 2.0 1.5 1.0 0.5 0.0 -0.5
f1 (ppm)

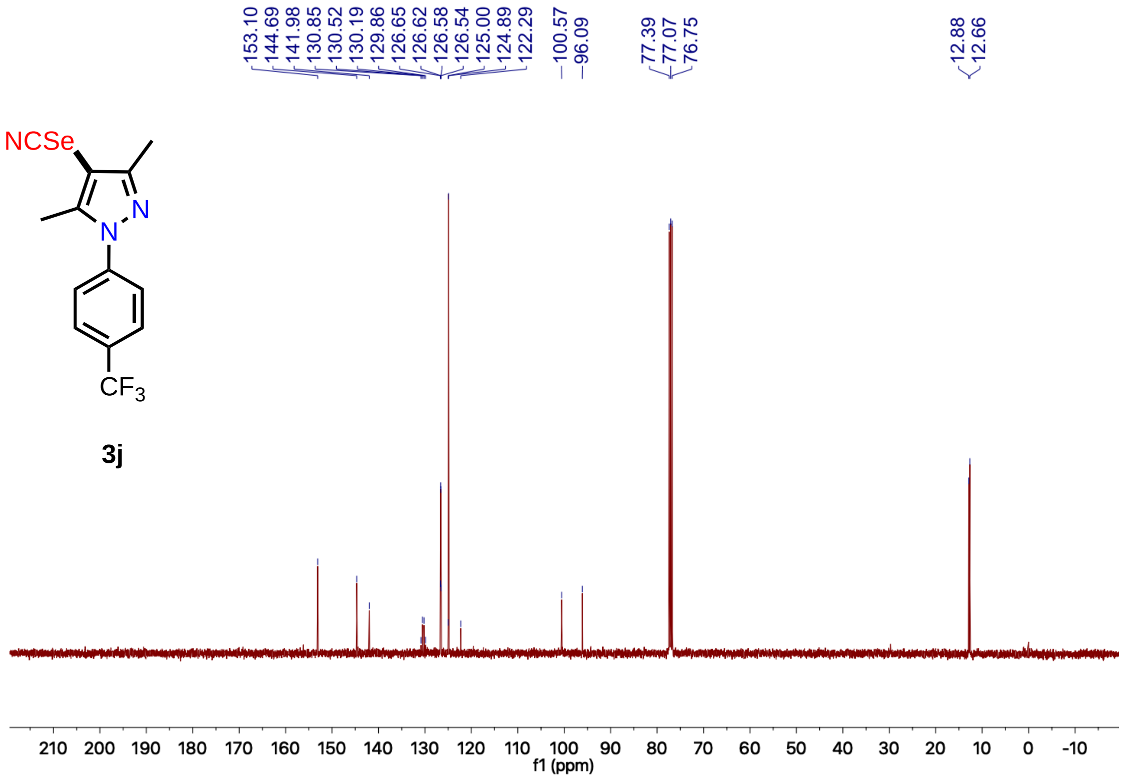
153.10
144.69
141.98
130.85
130.52
130.19
129.86
126.65
126.62
126.58
126.54
125.00
124.89
122.29
100.57
96.09
77.39
77.07
76.75
12.88
12.66
NCSe
N
N
CF3
3j
210 200 190 180 170 160 150 140 130 120 110 100 90 80 70 60 50 40 30 20 10 0 -10
f1 (ppm)

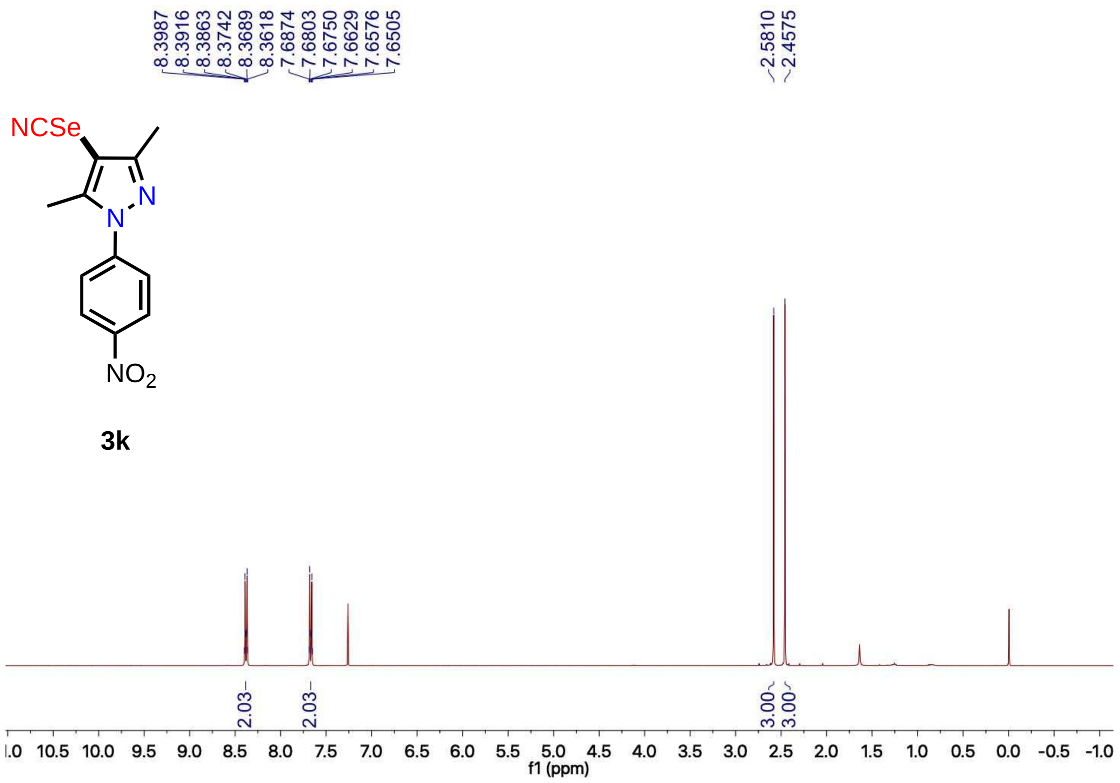
8.3987
8.3916
8.3863
8.3742
8.3689
8.3618
7.6874
7.6803
7.6750
7.6629
7.6576
7.6505
2.5810
2.4575
NCSe
N
N
NO_2
3k
2.03
2.03
3.00
3.00
f1 (ppm)

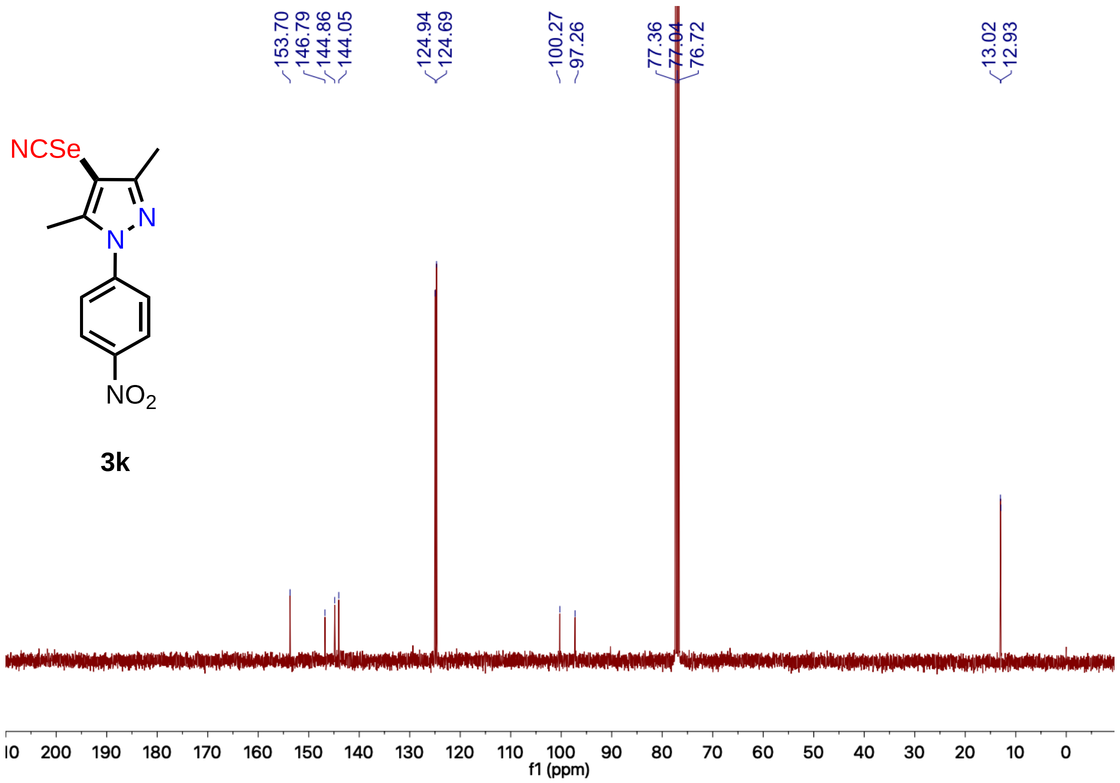
153.70
146.79
144.86
144.05
124.94
124.69
100.27
97.26
77.36
77.04
76.72
13.02
12.93
NCSe
N
N
NO_2
3k
f1 (ppm)

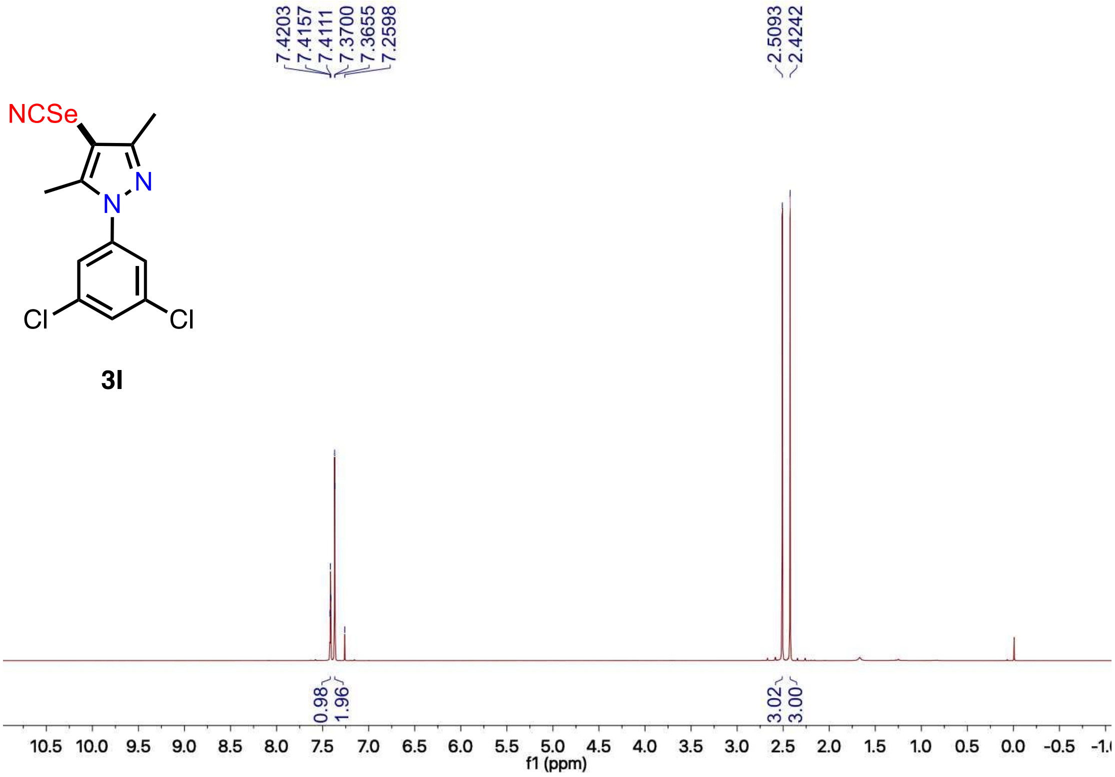
7.4203
7.4157
7.4111
7.3700
7.3655
7.2598
2.5093
2.4242
NCSe
N
N
Cl
Cl
0.98
1.96
3.02
3.00
f1 (ppm)

3l

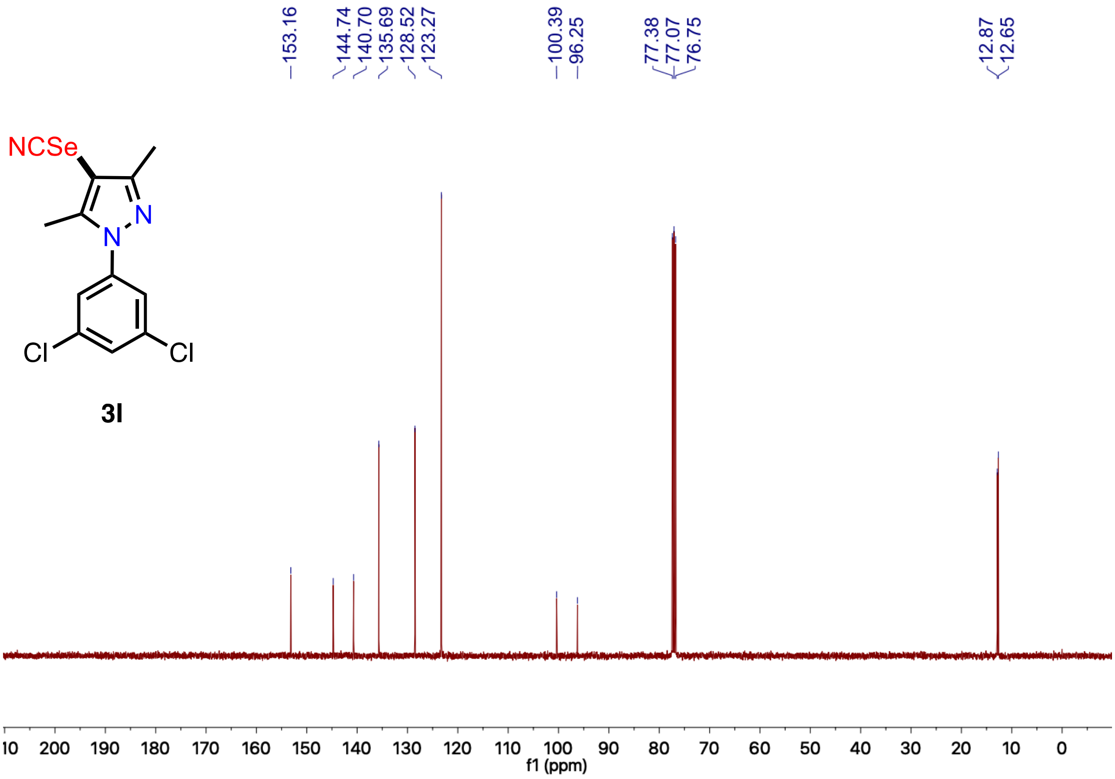
153.16
144.74
140.70
135.69
128.52
123.27
100.39
96.25
77.38
77.07
76.75
12.87
12.65
NCSe
N
N
Cl
Cl
f1 (ppm)

3l

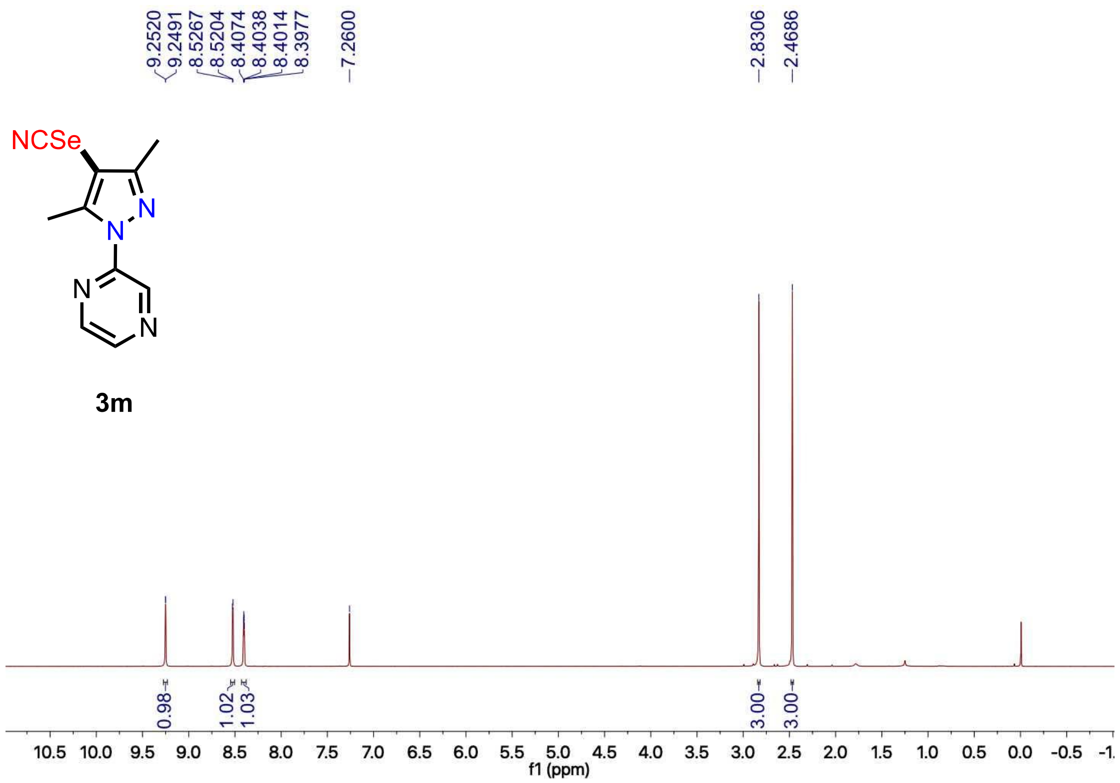
9.2520
9.2491
8.5267
8.5204
8.4074
8.4038
8.4014
8.3977
7.2600
2.8306
2.4686
NCSe
N
N
N
N
3m
0.98
1.02
1.03
3.00
3.00
10.5 10.0 9.5 9.0 8.5 8.0 7.5 7.0 6.5 6.0 5.5 5.0 4.5 4.0 3.5 3.0 2.5 2.0 1.5 1.0 0.5 0.0 -0.5
f1 (ppm)

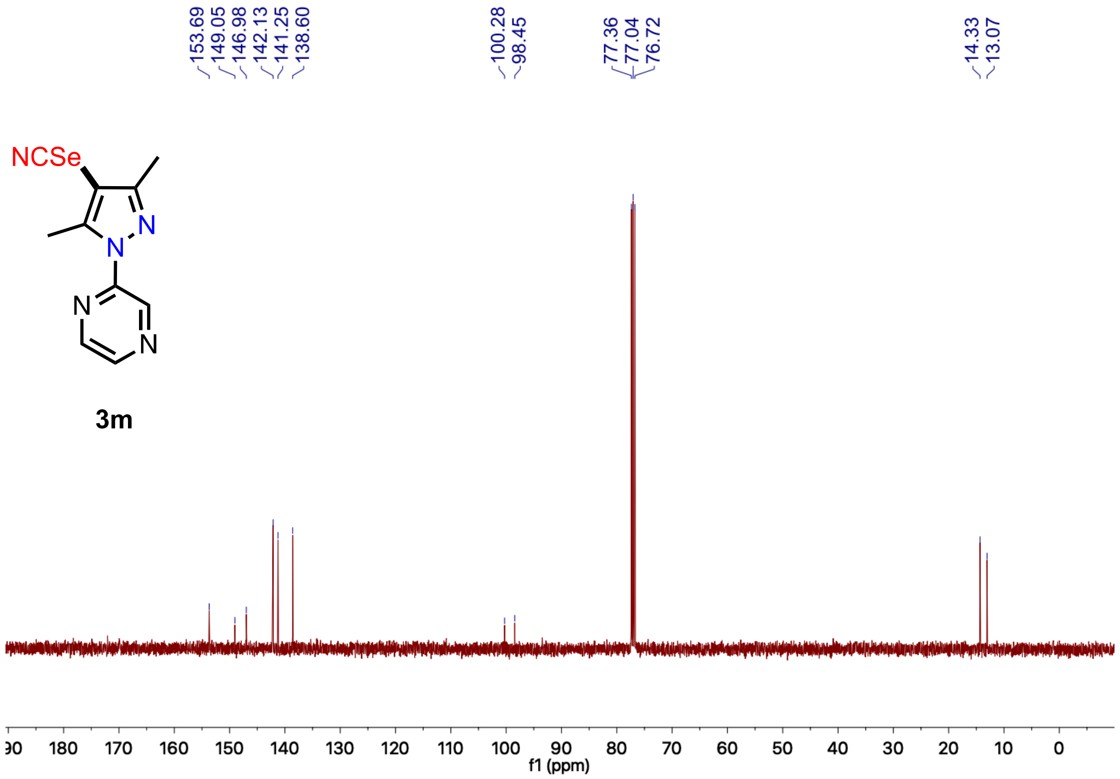
153.69
149.05
146.98
142.13
141.25
138.60
100.28
98.45
77.36
77.04
76.72
14.33
13.07
NCSe
N
N
N
N
3m
180 170 160 150 140 130 120 110 100 90 80 70 60 50 40 30 20 10 0
f1 (ppm)

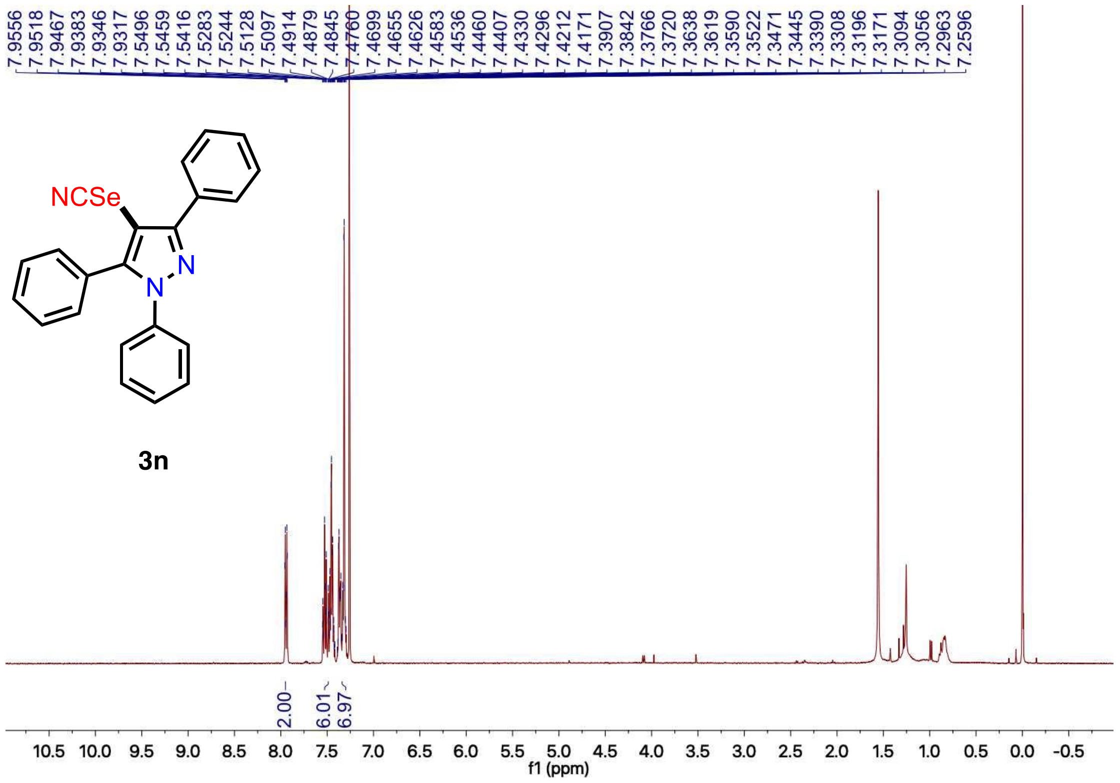
7.9556 7.9518 7.9467 7.9383 7.9346 7.9317 7.5496 7.5459 7.5416 7.5283 7.5244 7.5128 7.5097 7.4914 7.4879 7.4845 7.4760 7.4699 7.4655 7.4626 7.4583 7.4536 7.4460 7.4407 7.4330 7.4296 7.4212 7.4171 7.3907 7.3842 7.3766 7.3720 7.3638 7.3619 7.3590 7.3522 7.3471 7.3445 7.3390 7.3308 7.3196 7.3171 7.3094 7.3056 7.2963 7.2596
NCSe
N
N
3n
2.00
6.01
6.97
f1 (ppm)

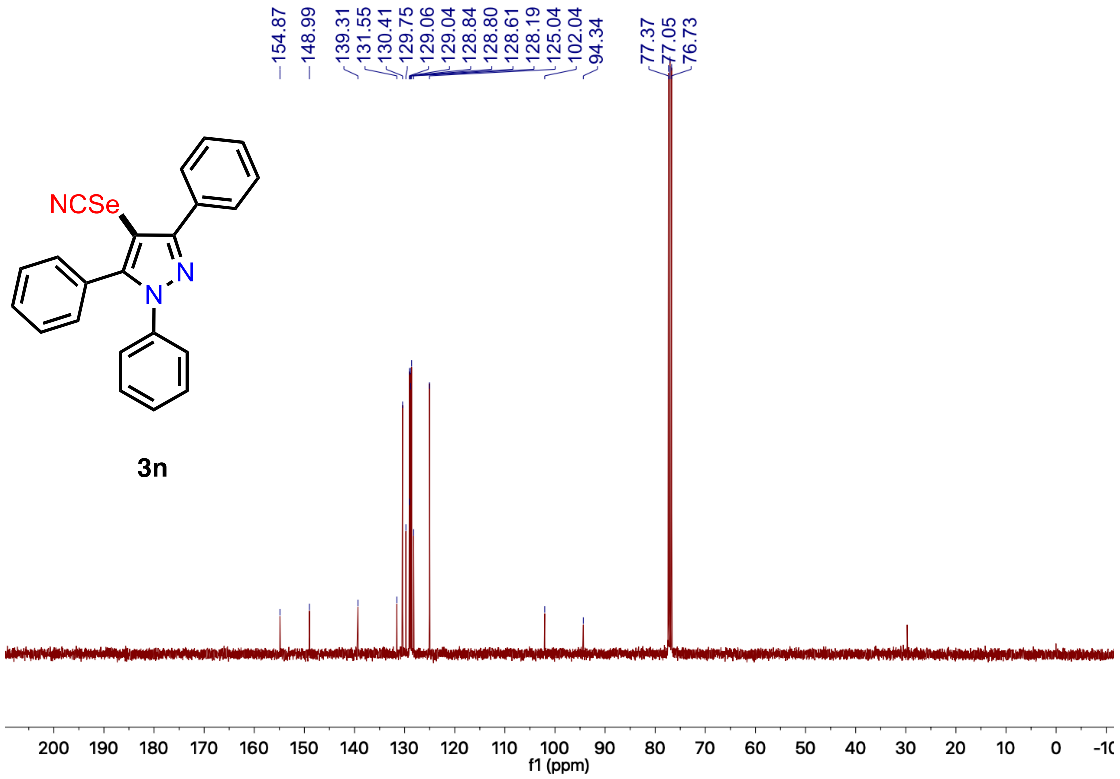
154.87
148.99
139.31 131.55 130.41 129.75 129.06 129.04 128.84 128.80 128.61 128.19 125.04 102.04 94.34
77.37 77.05 76.73
NCSe
N
N
3n
f1 (ppm)

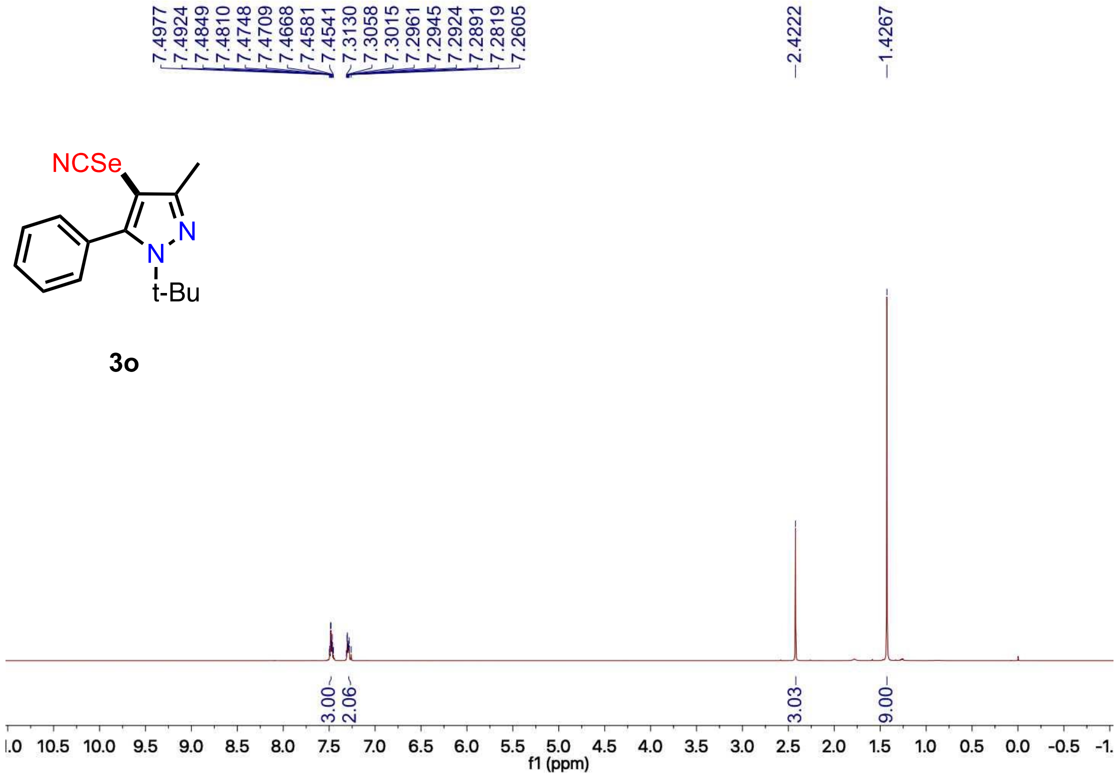
7.4977
7.4924
7.4849
7.4810
7.4748
7.4709
7.4668
7.4581
7.4541
7.3130
7.3058
7.3015
7.2961
7.2945
7.2924
7.2891
7.2819
7.2605
2.4222
1.4267
NCSe
N
N
t-Bu
3o
3.00
2.06
3.03
9.00
f1 (ppm)

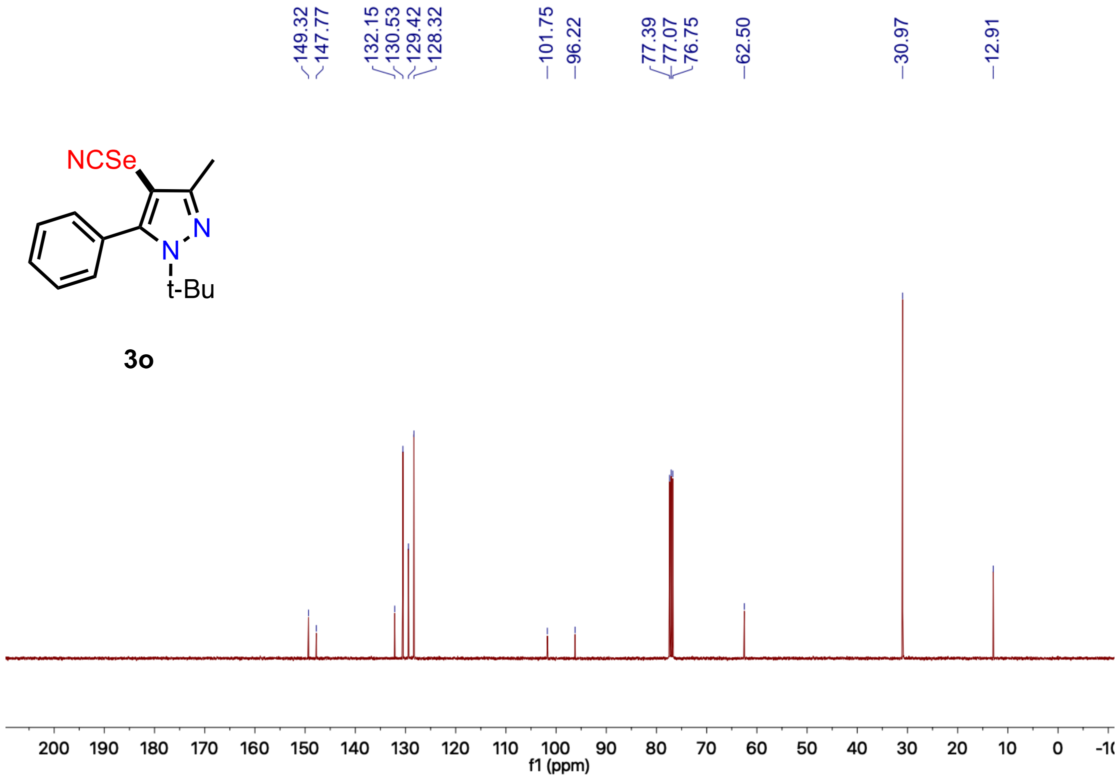
149.32
147.77
132.15
130.53
129.42
128.32
101.75
96.22
77.39
77.07
76.75
62.50
30.97
12.91
NCSe
N
N
t-Bu
3o
f1 (ppm)

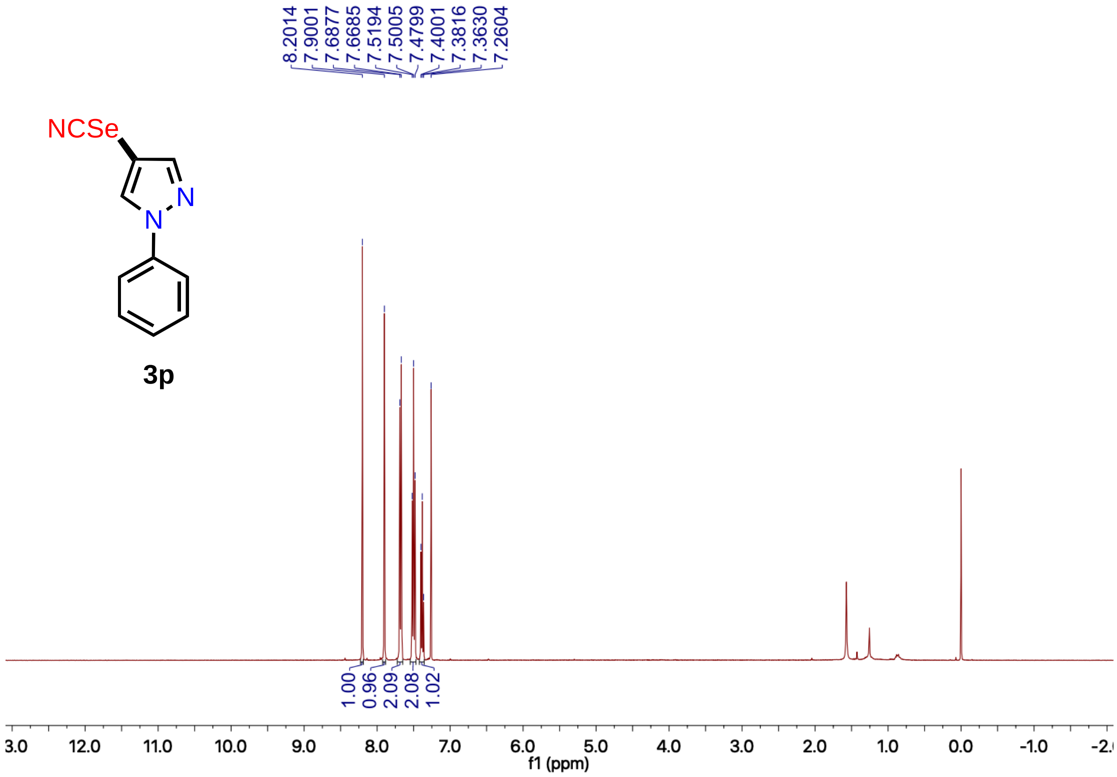
8.2014
7.9001
7.6877
7.6685
7.5194
7.5005
7.4799
7.4001
7.3816
7.3630
7.2604
NCSe
N
N
3p
1.00
0.96
2.09
2.08
1.02
f1 (ppm)

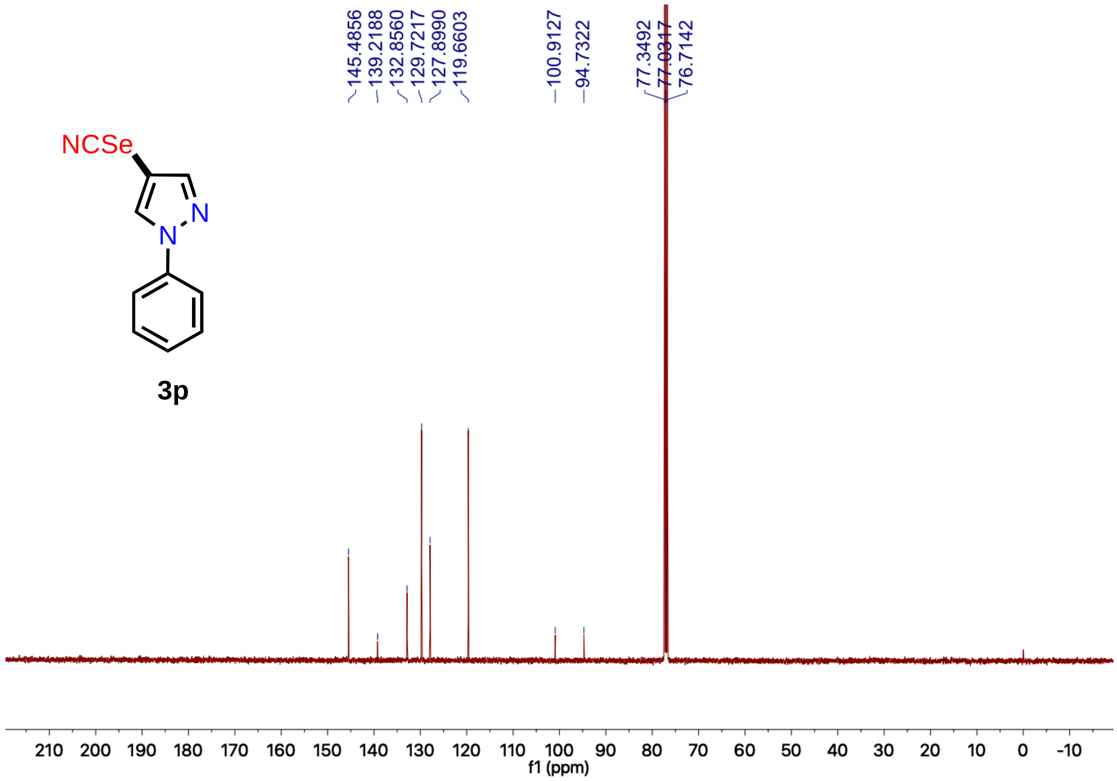
145.4856
139.2188
132.8560
129.7217
127.8990
119.6603
100.9127
94.7322
77.3492
77.0317
76.7142
NCSe
N
N
3p
f1 (ppm)

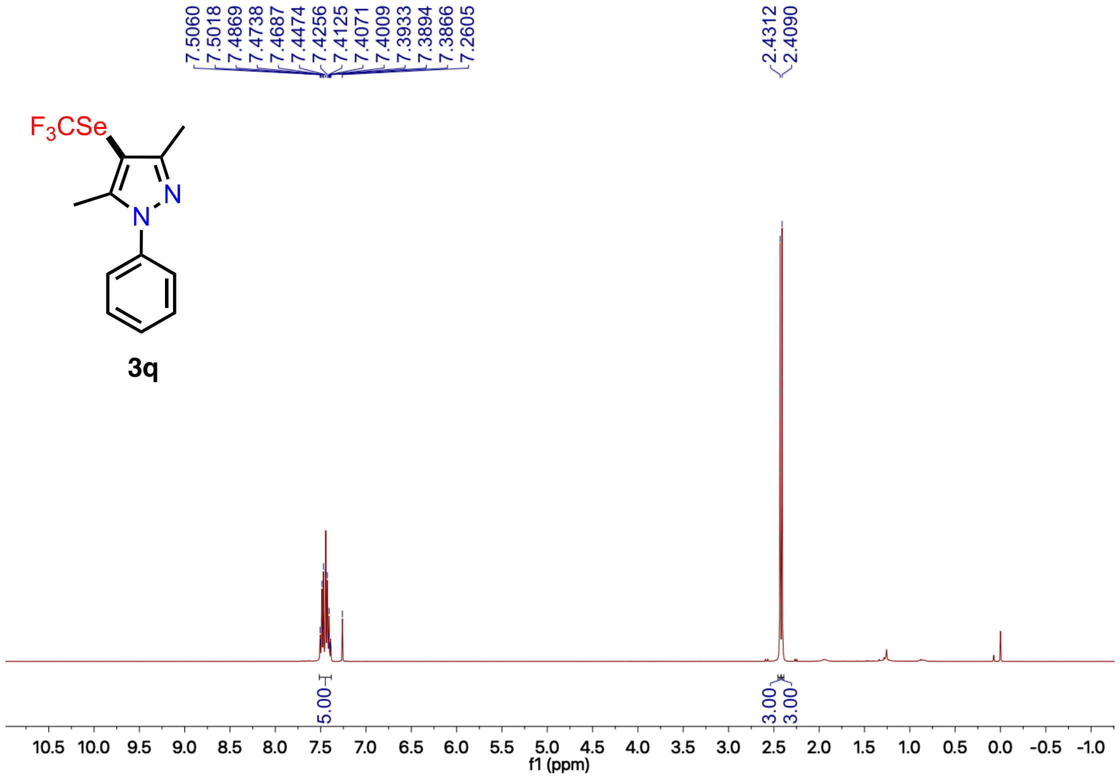
F3CSe
N
N
7.5060
7.5018
7.4869
7.4738
7.4687
7.4474
7.4256
7.4125
7.4071
7.4009
7.3933
7.3894
7.3866
7.2605
2.4312
2.4090
5.00
3.00
3.00
10.5 10.0 9.5 9.0 8.5 8.0 7.5 7.0 6.5 6.0 5.5 5.0 4.5 4.0 3.5 3.0 2.5 2.0 1.5 1.0 0.5 0.0 -0.5 -1.0
f1 (ppm)

3q

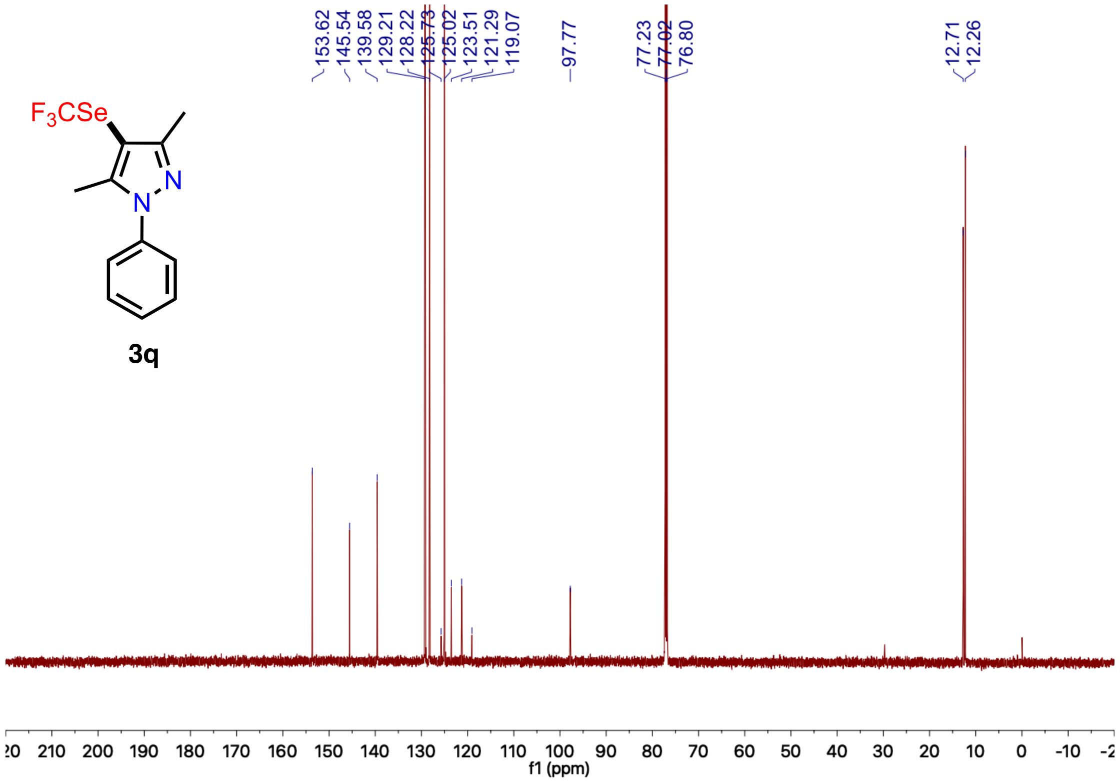
F3CSe
N
N
153.62
145.54
139.58
129.21
128.22
125.73
125.02
123.51
121.29
119.07
97.77
77.23
77.02
76.80
12.71
12.26
210 200 190 180 170 160 150 140 130 120 110 100 90 80 70 60 50 40 30 20 10 0 -10
f1 (ppm)

3q

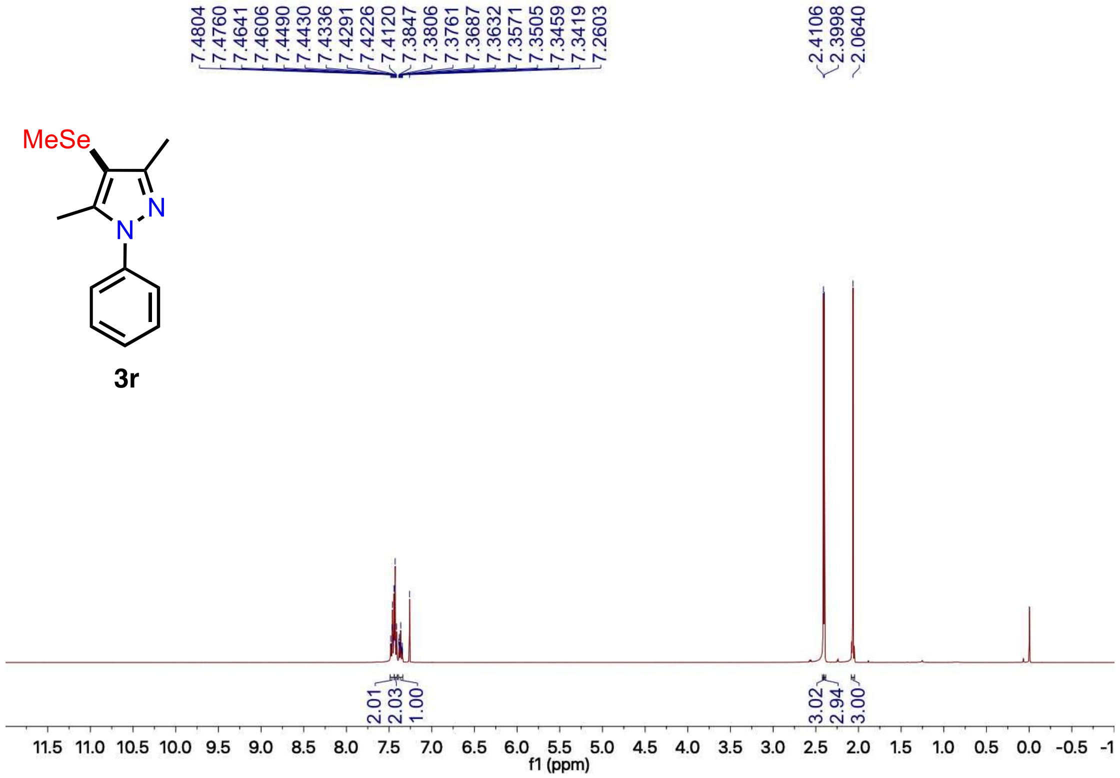
7.4804
7.4760
7.4641
7.4606
7.4490
7.4430
7.4336
7.4291
7.4226
7.4120
7.3847
7.3806
7.3761
7.3687
7.3632
7.3571
7.3505
7.3459
7.3419
7.2603
2.4106
2.3998
2.0640
MeSe
N
N
3r
2.01
2.03
1.00
3.02
2.94
3.00
11.5 11.0 10.5 10.0 9.5 9.0 8.5 8.0 7.5 7.0 6.5 6.0 5.5 5.0 4.5 4.0 3.5 3.0 2.5 2.0 1.5 1.0 0.5 0.0 -0.5 -1
f1 (ppm)

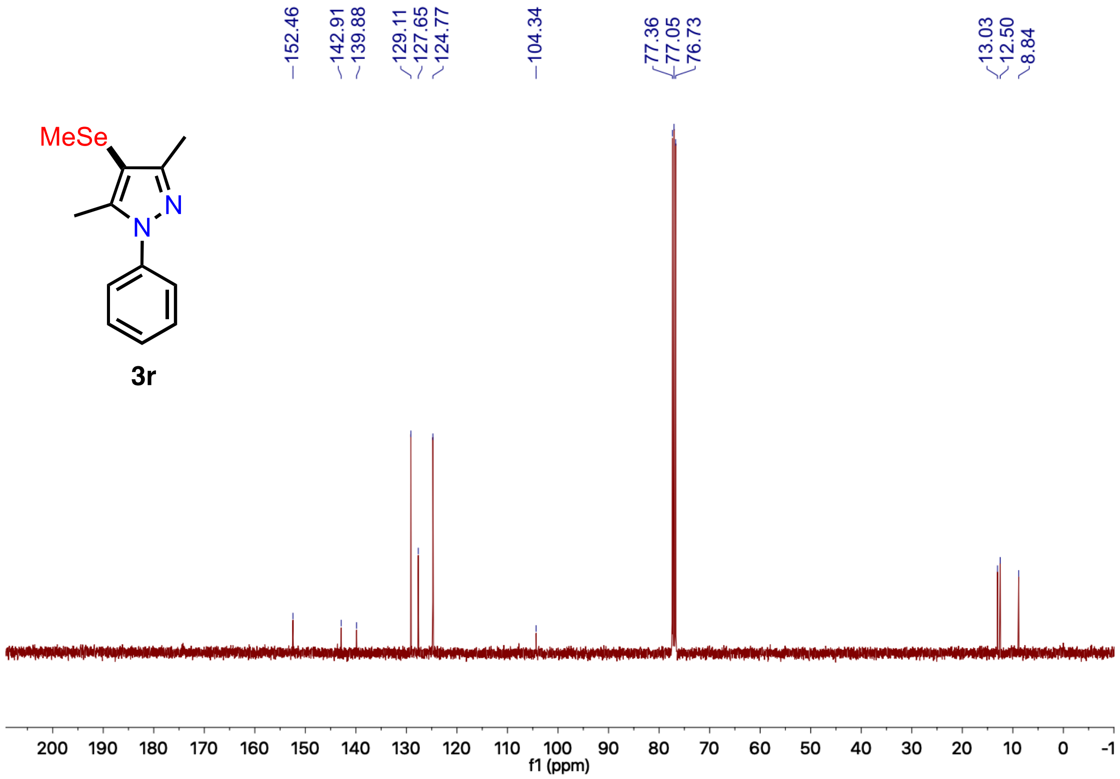
152.46
142.91
139.88
129.11
127.65
124.77
104.34
77.36
77.05
76.73
13.03
12.50
8.84
MeSe
N
N
3r
200 190 180 170 160 150 140 130 120 110 100 90 80 70 60 50 40 30 20 10 0 -1
f1 (ppm)
